# Association Mapping between Candidate Gene SNP and Production and Oil Quality Traits in Interspecific Oil Palm Hybrids

**DOI:** 10.3390/plants8100377

**Published:** 2019-09-26

**Authors:** Maider Astorkia, Mónica Hernandez, Stéphanie Bocs, Emma Lopez de Armentia, Ana Herran, Kevin Ponce, Olga León, Shone Morales, Nathalie Quezada, Francisco Orellana, Fahmi Wendra, Zulhermana Sembiring, Dwi Asmono, Enrique Ritter

**Affiliations:** 1 NEIKER Tecnalia, Campus Agroalimentario de Arkaute, Apdo 46. 01080 Vitoria-Gasteiz, Spain; mhernandez@neiker.eus (M.H.); eguzkisorgi@gmail.com (E.L.d.A.); aherran@neiker.eus (A.H.); eritter@neiker.eus (E.R.); 2 CIRAD, UMR AGAP, F-34398 Montpellier, France; stephanie.sidibe-bocs@cirad.fr; 3 AGAP, CIRAD, Univ Montpellier, INRA, Montpellier SupAgro, F-34398 Montpellier, France; 4 South Green Bioinformatics Platform, Bioversity, CIRAD, INRA, IRD, F-34398 Montpellier, France; 5 La Fabril SA, km 5.5 via Manta–Montecristi, Avenida 113, 130902 Manta, Ecuador; kponce@lafabril.com.ec (K.P.); smorales@lafabril.com.ec (S.M.); nquezada@lafabril.com.ec (N.Q.); 6 Energy & Palma SA, Av. Atahualpa E3-49 y Juan Gonzales, Ed. Fundación Pérez Pallarez, Officina 4ª, Quito 170507, Ecuador; oleon@energypalma.com (O.L.); forellana@energypalma.com (F.O.); 7 Department of Research & Development, PT Sampoerna Agro Tbk., Jl. Basuki Rahmat No. 788 Palembang 30127, Indonesia; fahmi.wendra@SampoernaAgro.com (F.W.); zulhermana.sembiring@SampoernaAgro.com (Z.S.); dwi.asmono@SampoernaAgro.com (D.A.)

**Keywords:** *Elaeis guineensis*, *Elaeis oleifera*, snakemake, lipids, tocols, carotene

## Abstract

Oil palm production is gaining importance in Central and South America. However, the main species *Elaeis guineensis* (Eg) is suffering severely from bud rod disease, restricting the potential cultivation areas. Therefore, breeding companies have started to work with interspecific *Elaeis oleifera* × Eg (Eo × Eg) hybrids which are tolerant to this disease. We performed association studies between candidate gene (CG) single nucleotide polymorphisms (SNP) and six production and 19 oil quality traits in 198 accessions of interspecific oil palm hybrids from five different origins. For this purpose, barcoded amplicons of initially 167 CG were produced from each genotype and sequenced with Ion Torrent. After sequence cleaning 115 SNP remained targeting 62 CG. The influence of the origins on the different traits was analyzed and a genetic diversity study was performed. Two generalized linear models (GLM) with principle component analysis (PCA) or structure (Q) matrixes as covariates and two mixed linear models (MLM) which included in addition a Kinship (K) matrix were applied for association mapping using GAPIT. False discovery rate (FDR) multiple testing corrections were applied in order to avoid Type I errors. However, with FDR adjusted *p* values no significant associations between SNP and traits were detected. If using unadjusted *p* values below 0.05, seven of the studied CG showed potential associations with production traits, while 23 CG may influence different quality traits. Under these conditions the current approach and the detected candidate genes could be exploited for selecting genotypes with superior CG alleles in Marker Assisted Selection systems.

## 1. Introduction

East-Asian countries address most of the oil palm production. Actually, Indonesia, Malaysia, and Thailand together produce almost 90% of the palm oil worldwide. Latin-American countries have started climbing positions in production few years ago, since Asian countries suffer lack of space due to increased oil demand and restricted cultivation areas [1]. Colombia, for example, has produced 1.68 million metric tons in 2019 [2] and ranks now fourth in the list of most productive countries. Moreover, two other Latin-American countries can be found among the top 10 palm oil producing countries in 2017; Ecuador and Honduras which produced 273,364 and 201,665 tons of oil, respectively [3]. 

However, the main oil palm species *Elaeis guineensis* (Eg) is suffering from bud rot disease “Pudrición de Cogollo” in these countries [4,5] leading to important economic losses, since most of the infected palms die. In order to face this situation, seed companies work now with interspecific hybrids between *Elaeis oleifera* and Eg (Eo × Eg) [6]. These hybrids combine desirable characteristics of both species; high oil production inherited from Eg and higher amounts of oleic and linoleic acids, vitamins, sterols, and iodine values, as well as resistance to different diseases descending from Eo [7,8]. Cadena et al. [9] reported an average of 71.5% oil in dry mesocarp of Eo × Eg interspecific hybrids, for commercial varieties of Eg var. tenera an average of 78% oil content and an average of 26.3% oil for Eo palms. They also reported the measured iodine values for these materials. Eo × Eg hybrid palms revealed an average iodine value of 66.3 g I_2_ 100 g^−1^, Eg palms showed 52 g I_2_ 100 g^–1^ and Eo palms an average of 77.4 g I_2_ 100 g^−1^. 

Many breeding and seed companies have started breeding programs to get elite hybrid palms. Marker-assisted selection has emerged as a useful technology for this purpose, particularly for traits controlled by multiple genes, such as those related to oil quality and oil quantity. However, until now only a few studies have been published on this topic. Montoya et al. [10] identified 19 quantitative trait loci (QTL) associated with fatty acid composition in an interspecific pseudo-backcross (Eo × Eg) × Eg. Singh et al. [11] constructed a linkage map using AFLP, RFLP, and SSR markers in an interspecific cross of a Colombian Eo and a Nigerian Eg accession and detected 11 QTL for iodine value and for six components of the fatty acid composition. Since these two studies were performed in specific mapping populations, the results may not be valid for other genetic backgrounds. Association Mapping (AM) based on linkage disequilibrium (LD) represents a way to avoid this problem, since a random population with unobserved ancestry can be studied [12,13]. While this technique is widely used in other crops, only a few articles have been published in Eg (The et al. [14], Kwong et al. [15], or Xia et al. [16]) and none in interspecific crosses of *Elaeis* species. Therefore, in the current study a broader collection of Eo × Eg hybrids was analyzed for different traits, divided in two big groups; production and quality traits. Production traits cover agronomic performance in terms of bunch number, bunch weight, and bunch yield and the oil contents in mesocarp and bunch. The analyzed oil quality traits considered different components of lipids and tocols, as well as carotenoids. Even though these last two represent only minor components, they are of nutritional importance [17]. The quality traits are described in detail under Material and Methods. The aim of this study was to determine via amplicon sequencing the allelic variation of potential candidate genes (CG) influencing these traits and to determine the effects of their particular single nucleotide polymorphisms (SNP) on trait expression, in order to exploit promising CG SNP for downstream applications in molecular breeding.

## 2. Results

### 2.1. Phenotype Analysis

Saphiro–Wilk tests revealed 16 traits which were not normally distributed. They are marked with “*” in Table 1. The ANOVA results for testing the influence of origins on the traits are presented in Supplementary Material, Table S1. Transformed data were used for non-normal distributed traits. Observed mean values, standard deviations (SD), minimum and maximum values, and the significance levels of the F tests are shown for each analyzed trait in Table S1. All production traits showed significant differences at significance level *p* < 0.001 as well as 16 quality traits. The SSS triglyceride (SSS), Delta compound (Delta), and Gamma compound (Gamma) traits did not reveal significant differences between origins.

The results of the Tukey post hoc tests are presented in Table 1. Production traits oil % in fresh mesocarp (OilfM), oil % in dry mesocarp (OildM), and oil % in bunch (OilB) revealed large values for the Coari × La Mé origin, while the Taisha × Ekona genotypes showed the lowest values for all production traits. On the other hand, Taisha × Avros (Oleoflores) revealed the highest values for bunch number (BN), bunch yield (BY), and bunch weight (BW) traits. For quality traits also a large difference was detected between Coari × La Mé and the other four origins. The Coari × La Mé origin showed statistically significant higher values for mono-unsaturated fatty acids % (Mono-Un), oleic acid % (OA), iodine value (IV), SUU triglyceride (SUU), or UUU triglyceride (UUU), but significant lower values than the other origins for saturated fatty acids % (Sat), poly-unsaturated fatty acids % (Poly-Un), SUS triglycerides (SUS), and tocopherol (Tocph) and tocotrienols (Toc3) compounds. 

### 2.2. Genotype Analysis

Three separate amplicon libraries were constructed with a total of 167 candidate genes. The first library was constructed from 56 candidate genes and yielded over 13.9 million raw reads. The second library from 55 CG produced around 9.2 million raw reads and the third library from 56 CG generated around 9.6 million raw reads. This total number of 32.7 million reads was reduced to 9.8 million clean reads after the filtering steps. Approximately 83% of the reads mapped to the Eg reference genome. The Snakemake-capture workflow identified initially 12,200 potential SNP. However, after the mentioned filtering steps, only 115 potential SNP remained for the following analyses. The average observed (Ho) and expected heterozygosity (He) were 0.61 and 0.37, respectively. Bartlett’s test revealed a significant difference between expected and observed heterozygosity. The fixation indices (Fst) values revealed no discriminant differentiation between populations as can be seen in Table 2, since all values were close to zero. With respect to the Fst values, the largest distances between origins were observed between Coari × La Mé and Taisha × Avros (Oleoflores) or Taisha × Yangambi, while the closest distances were observed between Coarí × La Mé and Taisha × Avros (RGS) and between Taisha × Ekona and Taisha × Yangambi. The inbreeding coefficients (Fis) values revealed no relatedness between individuals of the same origin since all obtained values were negative, suggesting a high diversity within origins. The Chi square tests indicated that only 38 of the markers were in Hardy–Weinberg equilibrium (HWE), while the other 77 showed significant deviations. 

### 2.3. Association Analysis

The remaining 115 SNP belong to 62 of the 167 initial CG used in the study and four of them showed multi locus mapping at two loci. SNP numbers for each candidate gene varied between one and four. The remaining CG are shown in Table S2 in Supplementary Material. Internal names for these 62 CG, the NCBI Gene ID, the CG position on the Malaysian Palm Oil Board (MPOB)’s reference genome, as well as the putative function of the CG are indicated in that Table.

**Figure 1:**
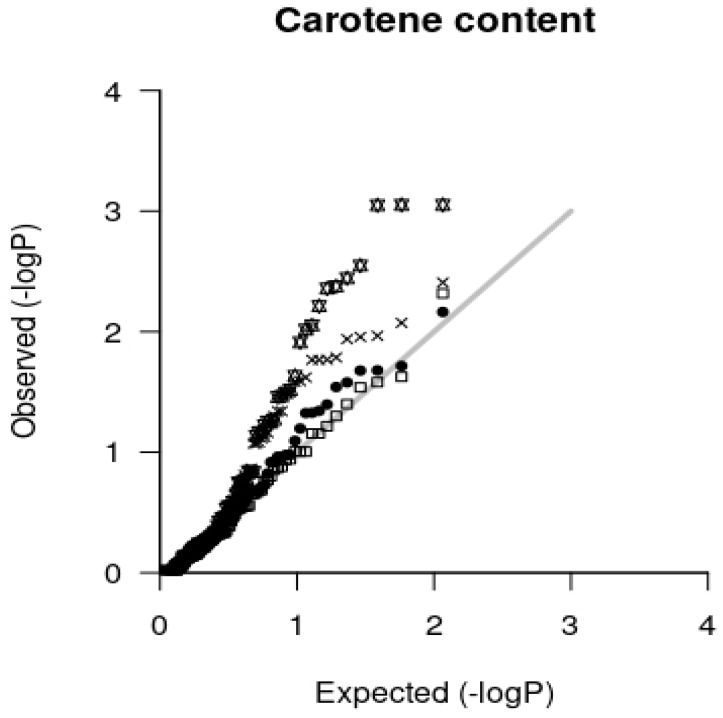
Example for a Quantile-Quantile (QQ) plot for Carotene contents (Car). Candidate gene (CG) data points of alternative generalized linear model (GLM) with structure matrix (Q) or principle component analysis matrix (PCA) as covariates: GLM_Q, GLM_PCA, respectively, and mixed linear models (MLM) incorporating in addition the IBS Kinship matrix (K) into the models: MLM_Q+K, MLM_PCA+K. They are represented by different symbols. (black circles: MLM_PCA+K; white squares: MLM_Q+K; stars: GLM_Q; crosses: GLM_PCA).

After running Association Mapping using GAPIT, expected and observed *p* values of each model were drawn as a Quantile-Quantile (QQ) plot for each trait. Figure 1 shows an example of a QQ plot for carotene contents (Car), reflecting the fitting of different alternative fixed generalized linear model (GLM) and fixed and random mixed linear models (MLM). The QQ plots for each trait are shown in Supplementary Material, Figure S2 The below described formula for calculating the average square distance (d^2^) of the CG data points from the diagonal of the QQ plot was applied for determining the best fitting model for each trait, even though in several cases the differences in the values for alternative models are very small. The results are shown in Table 3. For all production traits except OilfM MLM gave the best results. The OilfM trait fitted best with the GLM taking into account the structure matrix (Q) model; GLM_Q. OildM and OilB traits fitted best with the MLM using principle component analysis matrix (PCA) and IBS Kinship matrix (K); MLM_PCA+K and the three bunch related traits with MLM_Q+K models. Additionally, for most quality traits, mixed models were found to be the best fitting models, but some traits such as Alpha3 compound (Alpha3), Gamma, tocols (Toc), and Toc3 revealed better results with fixed effect models. Eight of the quality traits fitted better with MLM_PCA+K models and the other seven with MLM_Q+K models. 

Table 4 presents the results of association mapping. The detected associations based on observed unadjusted *p* values < 0.05 between CG SNP and traits are displayed, as well as the genome location of the significant SNP, the applied model, the significance level of the association, the explained variance, and the effect of the marker. The significant SNP which belong to a particular CG were grouped.

SNP belonging to a total of seven CG influenced significantly six production traits. Three CG revealed significant effects on two different production traits, while the other four CG influenced only one trait each, leading to a total of 10 significant associations for production traits. The BW trait was influenced by three different CG, OildM and OilB by two CG and BN, BY, and OilfM by only one CG. The explained variances by the model ranged from 8.9% to over 26% for the different CG. 

For quality traits SNP belonging to a total of 23 CG showed potential significant associations with 18 out of the 19 quality traits using unadjusted *p* values. Alph3 did not show any association with any of the studied CG SNP. The explained variances by the models ranged from 6.1% to over 28% of the total variance. For nine CG more than one SNP showed associations with different traits. Poly-Un showed associations with six of the studied CG and the Car trait revealed associations with five CG. Four potential associations were observed for the Delta, Delta3 compound (Delta3), Mono-Un, OA, SSS, and Toc traits and three associations for Alpha compound (Alpha), Gamma3 compound (Gamma3), IV, Toc3, Tocph, and UUU. SUU revealed two potential associations and Gamma, Sat and SUS showed only one potential association. It is also worth to notice, that five of the CG—EgNAC, PKP-ALPHA, SEQUI, LIPOIC, and TO1—showed also potential effects on different production traits. However, considering FDR adjusted *p* values, all detected associations are not significant anymore.

## 3. Discussion

### 3.1. Phenotypic Data Analysis

The analyses of production traits revealed larger differences between Coarí × La Mé genotypes and the other four origins where Taisha was involved. The Coarí × La Mé origin presented on average a higher oil to bunch (OilB) percentage and higher oil percentages in fresh and dry mesocarp (OilfM, OildM). Peláez et al. [5] observed that Coarí palms as well as their hybrids with *Eg* had higher CO_2_ fixation capacities, which are positively correlated with an increase in oil contents [17]. On the other hand, Taisha palms have been described by Barba [18] as “Oleifera Guineensis palms”, since they have similar morphological characteristics as *guineensis* palms. In our study we also found higher bunch weights (BW) in all origins involving Taisha and a higher bunch yield (BY) in the Taisha × Avros (Oleoflores) accessions. However, Arias et al. [19] studied different Eo origins and detected the highest total oil-per-bunch ratios [%] for Taisha accessions followed by Coarí accessions, indicating that there may be considerable variation between the particular accessions of the origins. From a commercial point of view (CPO, crude palm oil yield), also the industrial extraction rates have to be considered, which according to Soh et al. [20] are lower for hybrids involving Taisha. 

Considering analyses of quality traits, some studies are available from Montoya et al. [10], Singh et al. [11], and Cadena et al. [9]. These authors analyzed beside iodine value particularly the fatty acid composition in interspecific hybrids from controlled crosses and established linkage maps with integrated QTL for these traits. Cadena et al. studied the lipase activity, oil contents in fresh mesocarp, and iodine values in a collection of Eg, Eo, and Eo × Eg genotypes. However, we present here the first detailed oil quality analyses for oil palm involving 19 different quality traits. These include traits related to lipids where the saturation level of fatty acids was measured, considering the percentages of saturated (Sat), mono-unsaturated (Mono-un), and poly-unsaturated (poly-un) fatty acids. The mono-unsaturated fatty acids are considered as the healthiest [21,22]. We also analyzed the percentage of oleic acid in the oil (OA) which was classified as mono-unsaturated omega-9 fatty acid, the iodine value (IV) indicating the global degree of unsaturated fatty acids, and particularly the different types of triglycerides which can be formed from three fatty acids (SSS > SUS > SUU > UUU). We found large differences between Coari × La Mé and the other origins. Coari × La Mé accessions showed desirable characteristics such as high contents of mono-unsaturated acids, oleic acid, high iodine values, and UUU and SUU triglycerides, while the saturated acid levels were significantly below those of the other origins. Pelaez et al. [5] also determined higher oleic acid contents and iodine values in Coari palms. 

We performed also a detailed study for tocols contents which are composed of tocotrienols and tocopherols. These components represent different forms of vitamin E and can be found in oil palm as beneficial phytonutrients [23]. Both, tocotrienols and tocopherols have four isomers each (α-, β-, γ-, δ-) and have unique benefits [24]. Here we studied three of them (α-, β-, γ-). In contrary to what has been observed above, Coari × La Mé accessions showed significantly less contents of tocols. The α isomers from tocopherols and tocotrienols revealed lower quantities compared to the other four origins. Finally, carotenoids contents were measured in the five origins. These pigments are responsible for the orange-red brilliant color of the oil and are precursors of vitamin A [24]. For this trait the Taisha × Yangambi origin revealed the highest content. 

### 3.2. SNP Detection and Genetic Diversity Analysis

We used the Ion Torrent Personal Genome Machine (PGM) sequencing platform for convenience, based on previous experiences in other studies and ease of access. Similar studies using the PGM platform were also performed by other authors [25,26].

Mapping of the sequenced reads were performed using the published *Eg* var. pisifera genome sequence as reference. The decision to use this genome relied on the fact that actually no reference genome exists for Eo even though Singh et al. [27] published a draft. Nevertheless, Camillo et al. [28] analyzed genome sizes of Eg, Eo, and interspecific hybrids with the intention to reveal in the near future the genome sequence of Eo. When available, the genome sequences of both *Elaeis* species could be used as reference for mapping the sequence reads. 

In our analysis 83% of the reads could be mapped onto the reference genome and 12,200 SNP were identified initially. According to Singh et al. [27] 73% of the transposable element contents differ between Eg and Eo and could decrease the SNP numbers, since the reads in the hybrids descend from both *Elaeis* species. The high number of SNP was reduced drastically after the filtering steps and only 115 potential markers remained. The 62 targeted CG included two CG with multi-locus characteristics (PAT_2, ATAGB1), since they mapped to different chromosomes on the genome. These results suggest that the corresponding CG primers were specific for gene families rather than for individual CG. 

Random seed samples were received descending from multiple crosses made by Oleflores and RGS. However, nothing was known about the population structure a priori. Therefore, we performed some global genetic analyses. The Ho = 0.561 was significantly higher than the He = 0.37 in the accessions of all five origins. This high Ho value is in accordance with Arias et al. [19] who evaluated phenotypic and genetic diversity in two assays using of 13 and 19 SSR markers to characterize different Eo origins, including two Eo × Eg accessions and calculated Ho values of even 0.70 and 0.77 in the two assays, respectively. They also observed that 27% and 32% of the detected alleles in the study represented specific alleles of the different Eo origins and that one of the Eo × Eg accessions had the largest number of specific alleles. Arias et al. [29] found also for Eg accessions higher observed heterozygosity levels than the expected ones in most of the 23 analyzed origins. This can explain also the findings in our study since Eo origins from Brazil (Coarí) and from Ecuador (Taisha), as well as Eg origins from La Mé, Ekona, Yangambi, and Avros are incorporated into our hybrids. Furthermore, due to the nature of our F1 hybrids, it is expected to observe a higher Ho value.

According to Johnson and Shaw [30] the high Ho value is also coherent with the observed negative values of the computed Fis values in each of the five origins indicating high levels of genetic variability [30]. The observed high Ho value leads consequently also to high deviations from HWE (77 markers out of 115).

### 3.3. Association Mapping Results

Many studies have been published for the important oil palm crop Eg with the objective of crop improvement. However, the hybrids between the *Elaeis* species, which are so important in Latin-American regions, have been studied far less so far. Actually, only some QTL studies have been performed in order to improve the crop [10,11,31,32]. However, these studies consider structured (mapping) populations Here we performed a genotype-phenotype association study where the germplasm represents a random population with unobserved ancestry. 

In total four different models were used for association mapping. Two GLM models with population structure (GLM_Q) and principal component analysis (GLM_PCA) as covariates and two MLM models where in addition a K matrix between individuals was included (MLM_Q+K, MLM_PCA+K). After the analysis, the coincidence of observed and expected *p* values was visualized in a QQ plot for each trait. Several authors have used these QQ plots to determine the best fitting models visually [33,34,35]. 

When looking to the example of a QQ plot for carotenes contents in Figure 1, it can be seen clearly that the GLM_Q model represented by “stars” is the worst for fitting our data, while deciding visually between the other three models is impossible. Therefore, we developed an equation to calculate the average square distance (d^2^) of the CG data points from the diagonal of the QQ plot which represents an objective method for determining the best fitting model for each trait. 

In our study the mixed effects models fitted better for most of our traits, while only a few traits were found to have better associations with GLM models where the K matrix was not taken into account. These findings are in accordance with those of Wang et al. [36], Nigro et al. [37], or Lin et al. [35], who reported that MLM models were more appropriate for association studies in maize and wheat. 

As pointed out by Gao et al. [38] the output of FDR adjusted *p* values from GAPIT is highly stringent, leading to the loss of the detected significant associations using unadjusted *p* values. *A p* value of 0.05 was set as threshold for identifying potential CG with potential significant influence on a trait as also in other studies with similar approaches [38,39,40,41]. In total, seven CG were found to be related to six production traits and 23 CG to 18 quality traits (Table 4). With respect to the CG with significant effects, special attention has to be paid to eight of them (LIPOIC, SEQUI, TO1, EgNAC) with a potential relevant biological meaning. 

If not considering FDR adjusted *p* values, LIPOIC revealed potential associations with one production traits (OilfM,) and six quality traits (Gamma, OA, Mono-Un, Poly-Un, Toc, Toc3) It represents a lipoyl synthase gene, responsible for the synthesis of lipoic acid a universal antioxidant under oxidative stress conditions. This gene is required for cell growth, mitochondrial activity, and coordination of fuel metabolism and uses multiple mitochondrial 2-ketoacid dehydrogenase complexes [42] for the catalysis. Together with LIP2 it is essential for mitochondrial protein lipoylation during seed development [43]. It is known to be of high importance for obtaining high yielding plants [44]. 

Using unadjusted *p* values, also TO1 may influence the production traits BN and BW and one quality trait Gamma3. This CG represents a gamma-tocopherol methyltransferase which catalyzes the conversion of gamma-tocopherol into alpha-tocopherol. In *Arabidopsis* the overexpression of this enzyme resulted in more than 80-fold increase of α-tocopherol at the expense of γ-tocopherol without changing the total tocopherol contents [45]. 

The candidate gene SEQUI showed potential influence with one production trait, BW, and six quality traits; Toc, Toc3, Gamma3, SSS, IV, and Poly-Un. It is an alpha-humulene synthase transcript related to zerumbone biosynthesis. This compound is known as an essential oil of *C. verbenacea* and *Cannabis sativa* L. [46,47] and has healing effects as a multi-anticancer agent [48] and anti-inflamatory effects [47]. This compound also mediates the formation of beta-caryophyllene, another oil compound related to reduce systemic inflammation and oxidative stress [49]. 

Finally, EgNAC showed that it could be associated with seven quality traits and one production trait. NAC transcription factors have been studied widely in different crops. They are known to regulate different plant functions in plants, such as fruit ripening in tomato [50], variations in the protein content of wheat [51], increase in seed yield [52], and regulative functions for biotic and abiotic stress responses [53]. 

These findings indicate that many significant candidate genes could be involved in complex biological pathways, but there is still a lot of information missing. Fully understanding these metabolic pathways can help to discover the precise role of these genes influencing particular characters and can be a good starting point to obtain higher yielding oil palm varieties with increased oil contents. Association mapping results could be exploited in potential downstream applications by selecting genotypes with superior alleles of different significant candidate genes in Marker Assisted Selection systems.

Production traits are the most interesting characters from a commercial point of view. However, quality traits are becoming more and more important in recent years. Breeding Companies look for high quality oil properties in order to satisfy customer’s preferences. Components such as high levels of unsaturated acids, high carotene contents, or high amount of tocols are becoming more and more important traits for taking into account. Our association mapping approach and whole understanding of the function of these detected candidate genes could help to obtain improved palms with these desired qualities. 

In our study we only considered partial amplicons from a reduced number of candidate genes, limiting the scope of our approach. Further studies should be conducted in the future to improve the results, considering other molecular tools such as whole genome resequencing, transcriptome sequencing, or bait sequencing in order to increase the number of targets.

## 4. Material and Methods

### 4.1. Plant Material

A broader collection of 198 Eo × Eg F1 genotypes from five different origins were evaluated in the Energy and Palma plantation in San Lorenzo (Ecuador; 1.122980, −78.763190 GPS coordinates). These consisted of 40 hybrid genotypes from Coari × La Mé origin (Hacienda La Cabaña, Bogotá, Colombia), 75 accessions from Taisha × Avros (Oleoflores, Barranquilla, Colombia), 37 genotypes from Taisha × Avros (RGS, Quito, Ecuador), 21 genotypes from Taisha × Yangambi (RGS, Ecuador), and 25 genotypes from Taisha × Ekona (RGS, Ecuador).

### 4.2. Candidate Gene (CG) Selection

Partial amplicons from 167 CG related to oil production and oil quality were used for the analysis. These CG were identified randomly by in silico mining using different sources: (i) literature searches related to known genes from oil palm or other species with proven influence on the trait of interest, (ii) relevant patent sequences in oil palm and other species, (iii) exploration of relevant metabolic pathways such as palm oil biosynthesis for potentially useful enzymes, and (iv) analyses of published QTL and co-located transcripts with a relevant biological meaning. Amplicon primers for these CG were designed only in exons, but not in adjacent regulatory regions [54]. The CG name, the Gene ID from NCBI, the CG position according to the MPOB reference genome obtained by BLAST searches, the putative function of the CG and the forward and reverse primers used to obtain the partial amplicons can be found in Supplementary Material, Table S3.

### 4.3. Trait Recording

Eo × Eg genotypes were planted in 2010 and phenotypic data recording started in 2014. In total, six production traits and 19 quality traits were studied. The phenotypic raw data are shown in Supplementary Material, Table S4. 

The evaluated production traits were bunch number (BN; (nº)), bunch weight (BW; (kg)), bunch yield (BY = BN*BW; (kg)), oil percentage in fresh mesocarp (OilfM; (%)), oil percentage in dry mesocarp (OildM; (%)), and oil percentage in the bunch (OilB; (%)). BN and BW data were collected over four years and cumulative data were used for the analysis. OildM data was determined by Soxhlet extractions. OilfM and OilB were calculated according to García and Nañez [55] as modified by Arias et al. [19].

The analyzed oil quality traits considered different components of lipids and tocols, as well as carotenoids. Lipid components included percentages of oleic acid (OA), of saturated acids (Sat), mono-unsaturated acids (Mono-Un), and poly-unsaturated acids (Poly-Un) and were measured using the AOCS Official Ce-1h-05 [56] method. The iodine value (IV) in cg_iodine_/g was measured using the AOCS Official Da 15-48 method [57] and the percentages of the different types of triglycerides (SSS, SUS, SUU, UUU) were analyzed using the AOCS Official Ce-5C-93 method [58]. The nomenclature of the triglycerides indicate the saturation level of fatty acids at each of the three positions (S = saturated, U = unsaturated). Tocols (Toc) considered the sum of individual alpha, beta, gamma tocopherol´s (Tocph, Alpha, Beta, Gamma), and the sum of alpha3, beta3, gamma3 tocotrienols (Toc3, Alpha3, Beta3, Gamma3). All compounds were determined using the AOCS Official Ce 8-89 method [59] and are expressed in ppm. The carotene contents (Car; (ppm)) were measured using the PORIM p2.6 method [60]. 

Saphiro–Wilk tests were applied in order to check for non-normal distributed data. The traits that showed a significant deviation were normalized by z-score correction and the normalized data were further used for ANOVA analyses. ANOVA analyses of the different traits and origins were performed in order to see how the origin of the different accessions affects oil production and quality. Separation of means for traits with significant differences was performed using a Tukey post hoc test. All analyses were performed using R language. 

### 4.4. DNA Extraction and Library Construction

DNA extractions were performed from young leaflet tissue samples using the Analytik JenaLife extraction kit (Science Products, Jena, Germany) according to the manufacturer instructions. 

All PCR primers were designed in exons of the CG by blasting the CG against the oil palm genome sequence from MPOB [27] and using Primer3 software [61]. All amplification products were visualized via gel-electrophoresis in 1.5% TAE agarose gel stained with GelRed^®^ (Biotium, Fremont, CA, USA).

**Figure 2:**
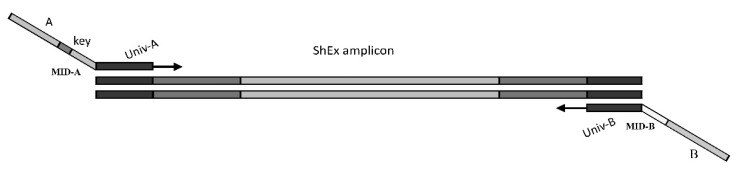
Scheme of the procedure for generating barcoded CG amplicons in oil palm hybrids. See text and Table S5 in Supplementary Materials for details.

Three amplicon libraries were constructed with a total of 167 CG in the mentioned plant materials. First and second libraries were constructed with 55 CG each, while the third had 57 CG. The CG for each library were chosen randomly. The library number in which a particular CG was included is indicated in Supplementary Materials Table S3. Amplicons for each CG were generated in a two-step PCR reaction as shown schematically in Figure 2, separately for each genotype.

For the first multiplex PCR reaction fusion primers were used which were composed of a universal part (UniA, UniB) and a part common to the CG of interest. These primers produced 120–300 bp amplicons. For each library several multiplex reactions were performed. For selecting appropriate primers for these multiplex reactions, each primer pair was tested with all others for Self-Dimers and Cross Primer Dimers formation using Thermo Fisher Multiple Primer Analyzer [62]. Sets of primers without dimer formation were used for each multiplex reaction.

A total of 20 ng of each genomic DNA, Invitrogen™ Platinum™ SuperFi™ PCR Master Mix (Life Technologies, Carlsbad, CA, USA), and 0.16 µM primer-mix were used per 25 µL amplification reaction. The PCR conditions were as follows: 98 °C denaturation for 30 s, followed by 30 cycles of 98 °C for 10 s, 58 °C for 30 s, 72 °C for 60 s, and a final elongation step of 72 °C for 5 min. PCR reactions were performed in a Thermal Cycler ABI 2720 (Applied Biosystems, Foster, USA). Amplification products were visualized as described and the PCR products were purified using Agencourt AMPure XP (Beckman Coulter, Indianapolis, IN, USA).

All purified multiplex PCR products of a specific genotype were combined in one pool and used in a second PCR reaction to barcode each genotype. For this purpose, fusion primers were designed which were composed of one part complementary to the universal part of UniA and UniB, a genotype specific MID part, the key part (ACGT) to calibrate the sequencing machine and the specific key sequences A and B used by the sequencing platform. All primers as well as the forward and reverse MID sequences are shown in Supplementary Materials, Table S5.

The genotype specific combinations of the MID sequences with UniA and UniB sequences, respectively, allow to identify unambiguously each genotype. By using a combination of forward and reverse MID a large number of genotypes can be barcoded with a relatively small number of primers. With 2n MID primers n^2^ genotypes can be discriminated. 

For each barcoding reaction, a 25 µL reaction volume was prepared containing 1 µL of the purified PCR product, 0.2 µM forward and reverse barcoding primer, and Invitrogen™ Platinum™ SuperFi™ PCR Master Mix. PCR reactions and visualization were performed as described before in the first step.

PCR products of each barcoded genotype were individually quantified with a Qubit 2.0 device, using the Qubit dsDNA HS assay (Life Technologies, Carlsbad, CA, USA). Equal concentrations of genotype specific PCR products were mixed in one tube.

Each pool was purified with columns using the GeneRead Size Selection Kit (Qiagen, Hilden, Germany). The quality of the libraries was verified on an Agilent 2100 Bioanalyzer using DNA Chips with HS DNA Kit reagents according to the manufacturer’s protocol (Agilent Technologies). The libraries were sent for sequencing to the Center for Applied Medical Research (CIMA, Pamplona, Spain), using the Ion Torrent PGM. Emulsion PCR was performed with Ion PGM™ Template OT2 400 Kit according to the manufacturer’s protocol. All libraries were sequenced using the 318 Chip v2 with the Ion PGM™ Sequencing 400 Kit. Sequencing was performed unidirectionally. 

### 4.5. Sequence Processing and Association Analysis

Analyses of the obtained sequences were performed using the South Green Bioinformatics Platform http://southgreen.cirad.fr/ [63], which provides different bioinfomatic tools and methods for sequence analysis.

The Fastq files of the three libraries were combined and processed together, since all genotypes had the same MID combination in the three libraries. In order to obtain clean amplicon sequences, trimming and demutliplexing steps were performed. First, each genotype was identified by the combination of MIDs in each read. Sequences were separated in genotype specific files. For this purpose the public “demultiplex.py” Python script [64] was used. Then, the “Cutadapt trimming tool” v1.8.1 [65] was applied to remove universal primer parts (UniA, UniB) and the MIDs. The cleaned, genotype specific sequences were processed using the “Snakemake-capture” script [66] of the South Green bioinformatics platform to map the reads using BWA v0.7.15 [67], to clean the alignments with Samtools v1.3 [68], to sort the reads with Picard-tools v2.7.0 [69] and to call the SNP using GATK haplotype caller v3.7-0 [70]. The MPOB *E. guineensis pisifera* genome sequence [27] was used as reference. 

The SNP of the obtained Variant Calling Format (VCF) file were filtered using VCFtools software v4.2 [71]. Markers were filtered for only biallelic SNP with a minimum allele frequency of 0.05 and a maximum of 0.95, markers below q < 30 were eliminated as well as indels. Additionally, variants with more than 20% of missing data were eliminated for the following analyses. Genetic diversity was studied in terms He and Ho of the markers using the adegenet [72] and hierfstat [73] packages in R. Monomorfic markers were eliminated for the following analyses. For studying genetic variances between and within origins, Fst obtained from VCFtools and Fis obtained from the hierfstat package were used. We tested also for HWE using the pegas package [74]. The null hypothesis (Ho = 0; *p* value < 0.05) was that the population is in equilibrium and pairing occurs randomly. fastStructure software v1.0 [75] was applied to analyze the population structure. Allele frequencies of each cluster from 1 to 9 were estimated with a 10-fold cross-validation (CV). In order to choose the appropriate number of model components explaining the structure in our dataset, thechooseK.py script of the fastStructure software was run. The distruct.py script from the fastStructure was used for drawing the distruct plot. 

Association studies were performed on a single marker basis using GAPIT v 3.0 [76] in R environment. Initially, fixed effects GLM were applied to test associations between segregating markers and phenotype for each trait. For this purpose, either Q matrix obtained from fastStructure (K = 6) was used as covariate, or PCA matrix with three components derived from GAPIT was used as covariate (GLM_Q, GLM_PCA). In addition, MLM analyses were performed in order to include both fixed and random effects. In this case, the IBS K matrix obtained from Tassel (v5.2.44) was incorporated into the previous models (MLM_Q+K, MLM_PCA+K) in order to reflect relationships among individuals with either the Q matrix or the PCA matrix. Multiple testing was also considered, since GAPIT provides beside unadjusted *p* values also FDR using the method of Benjamini and Hochberg [77] adjusted *p* values. 

The resulting observed and expected *p* values of each model were visualized separately for each trait in a QQ plot, in order to get a first impression on the fitting of different alternative models. In addition, an equation was developed to measure the average square distance (d^2^) of the CG data points from the diagonal of the QQ plot for each model:

(1) $$d^{2}=(\overset{\;n}{\underset{i=1}{\sum }}P_{o}{}^{2}+P_{e}{}^{2}-{\left(\frac{P_{o}+P_{e}}{2}\right)}^{2})/n,$$

where, *P_o_* and *P_e_* are the expected and observed –log(*p*) values, respectively and *n* the number of CG data points. The model with the smallest *d*^2^ value was considered as the best fitting model for our data.

## Figures and Tables

**Table 1:** Mean values of the studied traits for each origin and significant levels obtained by Tukey post hoc tests.

Origin:	Coari × LaMé		Taisha × Avros (RGS)		Taisha × Avros (Oleoflores)		Taisha × Econa		Taisha × Yangambi	
Production Traits	Mean Value	Level	Mean Value	Level	Mean Value	Level	Mean Value	Level	Mean Value	Level
BN (nº) *	52.49	B	39.81	C	63.00	A	32.75	C	40.10	BC
BW (kg)	9.44	B	11.04	B	13.22	A	9.81	B	9.91	B
BY (kg) *	501.67	B	469.32	B	845.66	A	334.30	B	444.75	B
OilfM (%)	34.69	A	28.99	B	29.31	B	24.74	C	28.71	BC
OildM (%) *	65.23	A	51.20	B	53.70	B	45.69	C	51.14	BC
OilB (%)	22.66	A	17.38	BC	19.67	B	14.09	C	17.04	BC
**Oil Quality Traits**										
Sat (%) *	32.07	B	37.91	A	38.64	A	39.39	A	40.00	A
Mono-Un (%) *	56.06	A	48.74	B	46.54	B	46.66	B	46.05	B
Poly-Un (%)	12.35	C	13.01	BC	14.31	A	13.68	AB	13.62	AB
OA (%) *	54.84	A	47.21	B	44.84	B	44.98	B	44.16	B
IV (cg/g) *	68.87	A	63.25	B	63.56	B	61.97	B	61.62	B
SSS (%) *	1.08	-	1.48	-	1.12	-	1.18	-	1.64	-
SUS (%) *	17.76	B	24.38	A	25.41	A	25.80	A	25.99	A
SUU (%)	35.82	A	31.95	B	31.40	B	31.77	B	29.44	B
UUU (%) *	21.06	A	12.01	B	10.28	B	10.23	B	9.90	B
Tocph (ppm) *	164.37	C	247.15	AB	198.63	BC	290.47	A	255.70	AB
Alpha (ppm) *	115.22	B	178.43	A	130.78	B	211.37	A	203.34	A
Delta (ppm) *	40.28	-	44.17	-	40.93	-	54.31	-	43.10	-
Gamma (ppm) *	39.49	-	46.64	-	47.29	-	47.35	-	42.15	-
Toc3 (ppm)	874.15	C	1087.74	B	1338.07	A	1159.80	AB	1065.70	BC
Alpha3 (ppm)	203.71	C	313.70	B	396.75	A	320.28	AB	314.24	AB
Delta3 (ppm) *	66.96	B	98.57	B	143.11	A	95.68	B	80.82	B
Gamma3 (ppm)	605.39	B	675.47	B	806.15	A	743.84	AB	670.64	B
Toc (ppm)	1038.52	B	1334.90	A	1543.12	A	1450.27	A	1321.40	AB
Car (ppm) *	785.89	BC	832.09	B	671.91	C	900.20	AB	1068.65	A

* Means with the same letter are not statistically different (α > 0.05). Traits marked with “*” did not follow a normal distribution according to Saphiro–Wilk tests. **Production traits:** bunch number (BN), bunch weight (BW), bunch yield (BY), oil % in fresh mesocarp (OilfM), oil % in dry mesocarp (OildM) and oil % in bunch (OilB). **Quality traits:** oleic acid % (OA), saturated fatty acids % (Sat), mono-unsaturated fatty acids % (Mono-Un), poly-unsaturated fatty acids % (Poly-Un), iodine value (IV), carotene contents (Car), different types of triglycerides in % (SSS, SUS, SUU, UUU), tocopherol (Tocph) compounds; Alpha, Delta, Gamma, tocotrienol (Toc3) compounds; Alpha3, Delta3, Gamma3, tocols (Toc).

**Table 2:** Genetic diversity studies in terms of inter cross Fixation indices (Fst) and intra cross Inbreeding coefficients (Fis).

Inter-Cross Fst Value	Taisha × Yangambi	Taisha × Ekona	Taisha × Avros (Oleoflores)	Taisha × Avros (RGS)	Coari × La Mé
Taisha × Yangambi	-	0.028876	0.055139	0.068303	0.10416
Taisha × Ekona	-	-	0.051121	0.064635	0.083617
Taisha × Avros (Oleoflores)	-	-	-	0.10259	0.10992
Taisha × Avros (RGS)	-	-	-	-	0.012305
**Intra-Cross Fis Values**	−0.7447191	−0.69170213	−0.72402062	−0.46477064	−0.46522124

Cluster analysis of the 115 markers by fastStructure for determining ancestry indicated that six sub-populations (K = 6) exists in our germplasm. These six cluster are represented in Figure S1 of the Supplementary Data as distruct plot. This parameter was also used for association mapping analyses.

**Table 3:** Average square distance (d^2^) values of the CG data points from the diagonal of the QQ plot for determining the best fitting model for each trait.

Production Traits	GLM_PCA	GLM_Q	MLM_PCA+K	MLM_Q+K
BN	0.4349	0.335	0.350	**0.286**
BY	0.369	0.335	0.298	**0.289**
BW	0.377	0.383	0.357	**0.332**
OilfM	0.294	**0.293**	0.294	0.294
OildM	0.281	0.285	**0.281**	0.327
OilB	0.331	0.337	**0.303**	0.458
**Oil Quality Traits**				
Sat	0.301	0.332	**0.270**	0.442
Mono-Un	0.305	0.352	**0.298**	0.317
Poly-Un	0.348	0.385	**0.347**	0.381
OA	0.333	0.365	**0.323**	0.426
IV	0.434	0.376	**0.327**	0.753
SSS	0.310	0.312	0.295	**0.292**
SUS	0.286	0.319	**0.285**	0.314
SUU	0.272	0.279	**0.271**	0.282
UUU	0.313	0.348	**0.306**	0.355
Tocph	0.333	0.355	0.323	**0.322**
Alpha	0.359	0.394	0.330	**0.327**
Delta	0.341	0.319	0.341	**0.317**
Gamma	0.265	**0.260**	0.265	0.266
Toc3	0.315	**0.306**	0.315	0.311
Alpha3	0.284	**0.264**	0.284	0.270
Delta3	0.329	0.382	0.315	**0.295**
Gamma3	0.342	0.339	0.337	**0.333**
Toc	0.325	**0.309**	0.325	0.316
Car	0.486	0.645	0.359	**0.334**

The best fitting model with smallest d^2^ value is indicated in bold and underlined for each CG.

**Table 4:** Results of association mapping between CG Single nucleotide polymorphisms (SNP) and production and oil quality traits in oil palm hybrids.

CG	SNP Position	Production Traits	AM Model	*p* Value	%VA	Effect
BKACPII_1	C10: 22949607	BW	MLM_Q	0.013	13.9	6.812
		BY	MLM_Q	0.037	26.2	538.811
EgNAC	C05: 40852639	OildM	MLM_PCA	0.044	18.3	−5.524
		OilB	MLM_PCA	0.046	8.9	−3.256
LIPOIC	C07: 18432097	OilfM	GLM_Q	0.042	10.9	−2.387
M2200	C13: 12503450	OildM	MLM_PCA	0.009	19.9	13.384
PKP-ALPHA	C01: 40816686	OilB	MLM_PCA	0.007	10.8	−9.339
SEQUI	U02: 19591286	BW	MLM_Q	0.015	14.1	2.319
TO1	U02: 79752170	BN	MLM_Q	0.020	24.4	−45.134
		BW	MLM_Q	0.033	14.8	−6.218
**CG Name**	**SNP Position**	**Quality Traits**	**AM Model**	***p* Value**	**%VA**	**Effect**
ATAGB1_ML *	C13: 103569	SSS	MLM_Q	0.022	7.2	−0.614
		Mono-Un	MLM_PCA	0.008	20.4	−5.291
		Poly-Un	MLM_PCA	0.047	10.7	1.136
ATP3	U05: 50035832	Mono-Un	MLM_PCA	0.050	18.7	−5.726
		Poly-Un	MLM_PCA	0.003	13.7	2.549
atpB	CT: 54552	Delta	MLM_Q	0.046	11.6	−6.913
BnC8_761	C08: 4351912	Delta3	MLM_Q	0.008	17.3	33.287
		OA	MLM_PCA	0.025	20.1	−2.488
		UUU	MLM_PCA	0.048	21.8	−2.250
CA3	C02: 35978226	Delta	MLM_Q	0.045	11.6	15.740
EgNAC	C05: 40852136	OA	MLM_PCA	0.015	20.6	2.890
		Sat	MLM_PCA	0.042	17.2	−1.990
		SUS	MLM_PCA	0.014	23.2	−2.189
		SUU	MLM_PCA	0.019	14.3	2.125
		UUU	MLM_PCA	0.007	23.5	3.246
	C05: 40852594	Mono-Un	MLM_PCA	0.044	18.8	3.568
		OA	MLM_PCA	0.005	21.7	4.826
		Poly-Un	MLM_PCA	0.010	12.3	−1.310
		SUS	MLM_PCA	0.009	23.6	−3.376
		UUU	MLM_PCA	0.003	24.3	5.081
	C05: 40852639	Car	MLM_Q	0.026	26.9	−173.576
EOCHYB	C04: 37534489	Alpha	MLM_Q	0.027	14.6	−50.440
GLUT1	C12: 28135330	OA	MLM_PCA	0.040	19.7	−2.823
	C12: 28135361	OA	MLM_PCA	0.040	19.7	−2.823
	C12: 28135379	OA	MLM_PCA	0.040	19.7	−2.823
HtC2_11412	C08: 25294023	Delta3	MLM_Q	0.036	15.7	27.356
		SUU	MLM_PCA	0.047	13.4	−1.552
	C08: 25294107	Delta3	MLM_Q	0.015	16.7	29.133
		SSS	MLM_Q	0.049	6.1	0.290
HtC2_1255C2-411	C02: 43975856	SSS	MLM_Q	0.046	6.1	0.529
	C02: 43975982	SSS	MLM_Q	0.046	6.1	0.529
HtC7_9200	C06: 41269483	Toc	GLM_Q	0.042	13.6	157.269
		Tocph	MLM_Q	0.046	13.2	35.249
	C06: 41269559	Car	MLM_Q	0.005	28.3	−158.848
JC35	C13: 22806955	Car	MLM_Q	0.024	27.0	−109.838
JC55	C05: 14759308	IV	MLM_PCA	0.007	15.1	7.826
LIPOIC	C07: 18431998	Gamma	GLM_Q	0.041	7.0	−7.841
		Mono-Un	MLM_PCA	0.039	18.9	−2.700
		OA	MLM_PCA	0.024	20.2	−2.842
		Poly-Un	MLM_PCA	0.037	11.0	0.782
	C07: 18432097	Gamma	GLM_Q	0.003	11.6	−11.135
		Toc	GLM_Q	0.043	13.5	−184.481
		Toc3	GLM_Q	0.027	16.3	−173.161
		Delta3	MLM_Q	0.033	16.0	−27.292
PAT_2	C09: 34725045	Alpha	MLM_Q	0.014	15.5	49.496
		Delta	MLM_Q	0.027	12.1	9.758
		Tocph	MLM_Q	0.005	15.4	70.245
PAT_2_ML	C02: 23775894	Poly-Un	MLM_PCA	0.035	11.0	−0.877
PAT_6	C08: 27075521	Car	MLM_Q	0.040	26.6	−163.806
PDHB	C01: 51857834	IV	MLM_PCA	0.027	13.8	3.407
PKP-ALPHA	C01: 40816686	UUU	MLM_PCA	0.034	22.1	−7.962
SEQUI	U02: 19591232	Toc	GLM_Q	0.031	13.9	260.781
		Toc3	GLM_Q	0.040	15.9	212.755
		Gamma3	MLM_Q	0.015	14.1	142.540
		SSS	MLM_Q	0.028	6.6	0.504
		IV	MLM_PCA	0.037	13.5	−2.828
		Poly-Un	MLM_PCA	0.020	11.6	−1.139
	U02: 19591286	IV	MLM_PCA	0.020	14.1	−3.630
SHELL	C02: 3078054	Alpha	MLM_Q	0.029	14.6	66.367
		Delta	MLM_Q	0.028	12.1	17.751
		Tocph	MLM_Q	0.019	14.1	90.283
	C02: 3078154	Toc	GLM_Q	0.031	13.9	213.715
		Toc3	GLM_Q	0.046	15.8	169.457
		Alpha	MLM_Q	0.048	14.2	37.502
		Delta3	MLM_Q	0.026	16.1	32.474
		Gamma3	MLM_Q	0.023	13.7	109.420
		Tocph	MLM_Q	0.029	13.7	51.563
TO1	U02: 79752182	Gamma3	MLM_Q	0.030	13.6	136.526
	U02: 79752184	Gamma3	MLM_Q	0.030	13.6	136.526
TO3	C03: 13885419	Car	MLM_Q	0.029	27.0	−157.239

Legend: CG Name: internal name of the CG; SNP position: genome location of the SNP; Trait: associated trait; Association Mapping (AM) Model: best fitting model for AM; *p* value: observed error probability value for the model; %VA: percentage of the total variance explained by the model; Effect: effect of the marker.

## Supplementary Materials

The following are available online at https://www.mdpi.com/2223-7747/8/10/377/s1, Table S1: Mean values, standard deviations (SD), minimum and maximum values of each analyzed trait, and ANOVA significance levels between the different origins of oil palm hybrids, Figure S1: Distruct plot of the six clusters used to explain our population structure, Table S2: List of the 62 Candidate Genes (CG) targeted by SNP which were used for the Association Mapping studies in oil palm hybrids, Figure S2: Quantile-Quantile plots of the different studied traits for the four tested models, Table S3: Characteristics of all 171 candidate genes initially analyzed by Amplicon sequencing in oil palm hybrids, Table S4: Raw phenotypic data from for each genotype, Table S5: Universal adapters and MID sequences used for generating barcoded amplicons of the different Candidate Genes (CG).

## References

[1] Seto K.C., Reenberg A. (2016). Rethinking Global Land Use in an Urban Era An Introduction.

[2] USDA (2019). Oilseeds: World Markets and Trades.

[3] Food and Agriculture Organization of the United Nations FAOSTAT. http://www.fao.org/faostat/en/#data/QC/visualize.

[4] Sundram S., Intan-Nur A.M.A. (2017). South American Bud rot: A biosecurity threat to South East Asian oil palm. Crop Prot..

[5] Pelaez E., Ramirez D., Gerardo C. (2010). Fisiología comparada de palmas africana (*Elaeis guineensis* Jacq.), americana (*Elaeis oleifera* hbk Cortes) e híbridos (*E. oleifera* × *E. guineensis*) en Hacienda La Cabaña. Palmas.

[6] Barba J. (2016). Introgresión de genes *E. guineensis* en híbridos interespecíficos O × G para recuperar la fertilidad del polen y otras características deseables en palma de aceite. Palmas.

[7] Torres M., Rey L., Gelves F., Santacruz L. (2004). Evaluación del comportamiento de los híbridos interespecíficos *Elaeis oleifera* × *Elaeis guineensis*, en la plantación de Guaicaramo S.A. Evaluation of the Behavior of Elaeis Oleifera × Elaeis Guineensis Hybrids in Guaicaramo Plantation. Palmas.

[8] Mohd D., Rajanaidu N., Jalani B.S. (2000). Performance of *Elaeis oleifera* from Panama, Costa Rica, Colombia and Honduras in Malasya. J. Oil Palm Res..

[9] Cadena T., Prada F., Perea A., Romero H.M. (2013). Lipase activity, mesocarp oil content, and iodine value in oil palm fruits of *Elaeis guineensis*, *Elaeis oleifera*, and the interspecific hybrid O × G (*E. oleifera* × *E. guineensis*). J. Sci. Food Agric..

[10] Montoya C., Lopes R., Flori A., Cros D., Cuellar T., Summo M., Espeout S., Rivallan R., Risterucci A.M., Bittencourt D. (2013). Quantitative trait loci (QTLs) analysis of palm oil fatty acid composition in an interspecific pseudo-backcross from *Elaeis oleifera* (H.B.K.) Cortés and oil palm (*Elaeis guineensis* Jacq.). Tree Genet. Genomes.

[11] Singh R., Tan S.G., Panandam J.M., Rahman R.A., Ooi L.C., Low E.-T.L., Sharma M., Jansen J., Cheah S.-C.C. (2009). Mapping quantitative trait loci (QTLs) for fatty acid composition in an interspecific cross of oil palm. BMC Plant Biol..

[12] Risch N., Merikangas K. (1996). The future of genetic studies of complex diseases. Science.

[13] Augusto A., Garcia F. (2001). Genetic Architecture of Quantitative Traits. Architecture.

[14] Teh C.K., Ong A.L., Kwong Q.B., Apparow S., Chew F.T., Mayes S., Mohamed M., Appleton D., Kulaveerasingam H. (2016). Genome-wide association study identifies three key loci for high mesocarp oil content in perennial crop oil palm. Sci. Rep..

[15] Kwong Q.B., Teh C.K., Ong A.L., Heng H.Y., Lee H.L., Mohamed M., Low J.Z.B., Apparow S., Chew F.T., Mayes S. (2016). Development and Validation of a High-Density SNP Genotyping Array for African Oil Palm. Mol. Plant.

[16] Xia W., Luo T., Zhang W., Mason A.S., Huang D., Huang X., Tang W., Dou Y., Zhang C., Xiao Y. (2018). Identification of genes affecting saturated fat acid content in Elaeis guineensis by genome-wide association analysis. BioRxiv.

[17] Corley R.H.V., Tinker P.B.H. (2015). The Oil Palm.

[18] Barba J. (2019). Oleiferas Ecuatorianas Alternativa de Manejo Agronomico para Compensar Las Perdidas Ocasionadas por la Pudricion del Cogollo en America Latina.

[19] Arias D., González M., Prada F., Ayala-Diaz I., Montoya C., Daza E., Romero H.M. (2015). Genetic and phenotypic diversity of natural American oil palm (*Elaeis oleifera* (H.B.K.) Cortés) accessions. Tree Genet. Genomes.

[20] Soh A.C., Mayes S., Roberts J.A. (2017). Oil Palm Breeding: Genetics and Genomics.

[21] Qian F., Korat A.A., Malik V., Hu F.B. (2016). Metabolic Effects of Monounsaturated Fatty Acid-Enriched Diets Compared with Carbohydrate or Polyunsaturated Fatty Acid-Enriched Diets in Patients with Type 2 Diabetes: A Systematic Review and Meta-analysis of Randomized Controlled Trials. Diabetes Care.

[22] Tierney A.C., Roche H.M. (2007). The potential role of olive oil-derived MUFA in insulin sensitivity. Mol. Nutr. Food Res..

[23] Nesaretnam K., Guthrie N., Chambers A.F., Carroll K.K. (1995). Effect of Tocotrienols on the Growth of a Human Breast Cancer Cell Line in Culture 1. Lipids.

[24] May C.Y., Nesaretnam K. (2014). Research advancements in palm oil nutrition. Eur. J. Lipid Sci. Technol..

[25] Guo L., Xia J., Yang S., Li M., You X., Meng Z., Lin H. (2015). GHRH, PRP-PACAP and GHRHR Target Sequencing via an Ion Torrent Personal Genome Machine Reveals an Association with Growth in Orange-Spotted Grouper (*Epinephelus coioides*). Int. J. Mol. Sci..

[26] Singh D., Singh B., Mishra S., Singh A.K., Singh N.K. (2019). Candidate gene based association analysis of salt tolerance in traditional and improved varieties of rice (*Oryza sativa* L.). J. Plant Biochem. Biotechnol..

[27] Singh R., Ong-Abdullah M., Low E.T.L., Manaf M.A.A., Rosli R., Nookiah R., Ooi L.C.L., Ooi S.E., Chan K.L., Halim M.A. (2013). Oil palm genome sequence reveals divergence of interfertile species in Old and New Worlds. Nature.

[28] Camillo J., Leão A.P., Alves A.A., Formighieri E.F., Azevedo A.L.S., Nunes J.D., de Capdeville G., de Mattos J.K.A., Souza M.T. (2014). Reassessment of the Genome Size in *Elaeis guineensis* and *Elaeis oleifera*, and Its Interspecific Hybrid. Genom. Insights.

[29] Arias D., Ochoa I., Castro F., Romero H. (2014). Molecular characterization of oil palm *Elaeis guineensis* Jacq. of different origins for their utilization in breeding programmes. Plant Genet. Resour..

[30] Johnson M.G., Shaw A.J. (2015). Genetic diversity, sexual condition, and microhabitat preference determine mating patterns in Sphagnum (Sphagnaceae) peat-mosses. Biol. J. Linn. Soc..

[31] Ting N.C., Jansen J., Mayes S., Massawe F., Sambanthamurthi R., Ooi L.C.L., Chin C.W., Arulandoo X., Seng T.Y., Alwee S.S.R.S. (2014). High density SNP and SSR-based genetic maps of two independent oil palm hybrids. BMC Genom..

[32] Ting N.-C., Yaakub Z., Kamaruddin K., Mayes S., Massawe F., Sambanthamurthi R., Jansen J., Eng Ti Low L., Ithnin M., Kushairi A. (2016). Fine-mapping and cross-validation of QTLs linked to fatty acid composition in multiple independent interspecific crosses of oil palm. BMC Genom..

[33] Álvarez M.F., Angarita M., Delgado M.C., García C., Jiménez-Gomez J., Gebhardt C., Mosquera T. (2017). Identification of Novel Associations of Candidate Genes with Resistance to Late Blight in Solanum tuberosum Group Phureja. Front. Plant Sci..

[34] Gamazon E.R., Wheeler H.E., Shah K.P., Mozaffari S.V., Aquino-Michaels K., Carroll R.J., Eyler A.E., Denny J.C., Nicolae D.L., Cox N.J. (2015). A gene-based association method for mapping traits using reference transcriptome data. Nat. Genet..

[35] Lin Y., Liu S., Liu Y., Liu Y., Chen G., Xu J., Deng M., Jiang Q., Wei Y., Lu Y. (2017). Genome-wide association study of pre-harvest sprouting resistance in Chinese wheat founder parents. Genet. Mol. Biol..

[36] Wang M., Yan J., Zhao J., Song W., Zhang X., Xiao Y., Zheng Y. (2012). Genome-wide association study (GWAS) of resistance to head smut in maize. Plant Sci..

[37] Nigro D., Gadaleta A., Mangini G., Colasuonno P., Marcotuli I., Giancaspro A., Giove S.L., Simeone R., Blanco A. (2019). Candidate genes and genome-wide association study of grain protein content and protein deviation in durum wheat. Planta.

[38] Gao L., Turner M.K., Chao S., Kolmer J., Anderson J.A. (2016). Genome Wide Association Study of Seedling and Adult Plant Leaf Rust Resistance in Elite Spring Wheat Breeding Lines. PLoS ONE.

[39] Li Y., Wilcox P., Telfer E., Graham N., Stanbra L. (2016). Association of single nucleotide polymorphisms with form traits in three New Zealand populations of radiata pine in the presence of genotype by environment interactions. Tree Genet. Genomes.

[40] Zegeye H., Rasheed A., Makdis F., Badebo A., Ogbonnaya F.C. (2014). Genome-Wide Association Mapping for Seedling and Adult Plant Resistance to Stripe Rust in Synthetic Hexaploid Wheat. PLoS ONE.

[41] Pasam R.K., Sharma R., Malosetti M., van Eeuwijk F.A., Haseneyer G., Kilian B., Graner A. (2012). Genome-wide association studies for agronomical traits in a world wide spring barley collection. BMC Plant Biol..

[42] Solmonson A., Deberardinis R.J. (2017). Lipoic acid and mitochondrial redox regulation. J. Biol. Chem..

[43] Ewald R., Hoffmann C., Florian A., Neuhaus E., Fernie A.R., Bauwe H. (2014). Lipoate-Protein Ligase and Octanoyltransferase Are Essential for Protein Lipoylation in Mitochondria of Arabidopsis. Plant Physiol..

[44] Schoen H., Thimm O., Ritte G., Blaesing O., Bruynseels K., Hatzfeld Y., Frankard V. (2010). Plants with increased yield (NUE).

[45] Shintani D., DellaPenna D. (1998). Elevating the vitamin E content of plants through metabolic engineering. Science.

[46] Benelli G., Pavela R., Petrelli R., Cappellacci L., Santini G., Fiorini D., Sut S., Dall’Acqua S., Canale A., Maggi F. (2018). The essential oil from industrial hemp (*Cannabis sativa* L.) by-products as an effective tool for insect pest management in organic crops. Ind. Crops Prod..

[47] Fernandes E.S., Passos G.F., Medeiros R., Da Cunha F.M., Ferreira J., Campos M.M., Pianowski L.F., Calixto J.B. (2007). Anti-inflammatory effects of compounds alpha-humulene and (−)-trans-caryophyllene isolated from the essential oil of Cordia verbenacea. Eur. J. Pharmacol..

[48] Yu F., Okamto S., Nakasone K., Adachi K., Matsuda S., Harada H., Misawa N., Utsumi R. (2008). Molecular cloning and functional characterization of-humulene synthase, a possible key enzyme of zerumbone biosynthesis in shampoo ginger (*Zingiber zerumbet* Smith). Planta.

[49] Ames-Sibin A.P., Barizão C.L., Castro-Ghizoni C.V., Silva F.M.S., Sá-Nakanishi A.B., Bracht L., Bersani-Amado C.A., Marçal-Natali M.R., Bracht A., Comar J.F. (2018). β-Caryophyllene, the major constituent of copaiba oil, reduces systemic inflammation and oxidative stress in arthritic rats. J. Cell. Biochem..

[50] Kou X., Liu C., Han L., Wang S., Xue Z. (2016). NAC transcription factors play an important role in ethylene biosynthesis, reception and signaling of tomato fruit ripening. Mol. Genet. Genom..

[51] Hu X.-G., Wu B.-H., Liu D.-C., Wei Y.-M., Gao S.-B., Zheng Y.-L. (2013). Variation and their relationship of NAM-G1 gene and grain protein content in *Triticum timopheevii* Zhuk. J. Plant Physiol..

[52] Liang C., Wang Y., Zhu Y., Tang J., Hu B., Liu L., Ou S., Wu H., Sun X., Chu J. (2014). OsNAP connects abscisic acid and leaf senescence by fine-tuning abscisic acid biosynthesis and directly targeting senescence-associated genes in rice. Proc. Natl. Acad. Sci. USA.

[53] Nuruzzaman M., Sharoni A.M., Kikuchi S. (2013). Roles of NAC transcription factors in the regulation of biotic and abiotic stress responses in plants. Front. Microbiol..

[54] López de Armentia A. (2017). Mapeo por Asociación Mediante Genes Candidatos en Palmera de Aceite Africana (E. guineensis Jacq.). Ph.D. Thesis.

[55] García J.A., Yáñez E.E. (2000). Aplicación de la metodología alterna para análisis de racimos y muestreo de racimos en tolva. Palmas.

[56] AOCS (2017). AOCS Official Method Ce 1h-05. Official Methods and Recommended Practices of the AOCS.

[57] AOCS (2017). AOCS Official Method Da 15-48. Official Methods and Recommended Practices of the AOCS.

[58] AOCS (2017). AOCS Official Method Ce 5c-93. Official Methods and Recommended Practices of the AOCS.

[59] AOCS (2017). AOCS Official Method Ce 8-89. Official Methods and Recommended Practices of the AOCS.

[60] Siew W.L., Tang T.S. (1995). PORIM p2.6 Method. PORIM Test Methods.

[61] Untergasser A., Cutcutache I., Koressaar T., Ye J., Faircloth B.C., Remm M., Rozen S.G. (2012). Primer3-new capabilities and interfaces. Nucleic Acid Res..

[62] Thermo Fisher Scientific Multiple Primer Analyzer. https://www.thermofisher.com/es/es/home/brands/thermo-scientific/molecular-biology/molecular-biology-learning-center/molecular-biology-resource-library/thermo-scientific-web-tools/multiple-primer-analyzer.html.

[63] South Green Collaborators (2016). The South Green portal: A comprehensive resource for tropical and Mediterranean crop genomics. Curr. Plant Biol..

[64] Flutre T., Gay L., Rode N. GitHub. https://github.com/timflutre/quantgen/blob/master/demultiplex.py.

[65] Martin M. (2011). Cutadapt removes adapter sequences from high-throughput sequencing reads. EMBnet. J..

[66] Soriano A., Guitton P., Mournet P. Workflow-Snakemake-Capture. https://github.com/SouthGreenPlatform/Workflow-snakemake-capture.

[67] Li H., Durbin R. (2010). Fast and accurate long-read alignment with Burrows–Wheeler transform. Bioinformatics.

[68] Li H., Handsaker B., Wysoker A., Fennell T., Ruan J., Homer N., Marth G., Abecasis G., Durbin R. (2009). 1000 Genome Project Data Processing Subgroup the Sequence Alignment/Map format and SAMtools. Bioinformatics.

[69] Picard Tools—By Broad Institute. https://broadinstitute.github.io/picard/index.html.

[70] McKenna A., Hanna M., Banks E., Sivachenko A., Cibulskis K., Kernytsky A., Garimella K., Altshuler D., Gabriel S., Daly M. (2010). The Genome Analysis Toolkit: A MapReduce framework for analyzing next-generation DNA sequencing data. Genome Res..

[71] Danecek P., Auton A., Abecasis G., Albers C.A., Banks E., Depristo M.A., Handsaker R.E., Lunter G., Marth G.T., Sherry S.T. (2011). The variant call format and VCFtools. Bioinformatics.

[72] Jombart T. (2008). Adegenet: A R package for the multivariate analysis of genetic markers. Bioinformatics.

[73] Goudet J., Jombart T., Goudet M.J. (2015). Package “hierfstat”: Estimation and Tests of Hierarchical F-Statistics. https://rdrr.io/cran/hierfstat/.

[74] Paradis E., Jombart T., Brian K., Schliep K., Winter D., Kamvar Z.N. (2018). Package “pegas”: Population and Evolutionary Genetics Analysis System. https://cran.r-project.org/web/packages/pegas/index.html.

[75] Raj A., Stephens M., Pritchard J.K. (2014). fastSTRUCTURE: Variational inference of population structure in large SNP data sets. Genetics.

[76] Wang J., Zhang Z. (2018). GAPIT Version 3: An Interactive Analytical Tool for Genomic Association and Prediction. https://www.researchgate.net/publication/329829469_GAPIT_Version_3_An_Interactive_Analytical_Tool_for_Genomic_Association_and_Prediction.

[77] Benjamini Y., Hochberg Y. (1995). Controlling the False Discovery Rate: A Practical and Powerful Approach to Multiple Testing. J. R. Stat. Soc. Ser. B.

