# Supplementary material for: Association Mapping between Candidate Gene SNP and Production and Oil Quality Traits in Interspecific Oil Palm Hybrids
Source: Plants (Basel). 2019 Sep 26;8(10):377. doi: 10.3390/plants8100377 (PMC6843369; doi:10.3390/plants8100377)
Supplement: Supplementary file 1 [file plants-08-00377-s001.pdf]

SUPLEMENTARY MATERIAL

Table S1. Mean values, standard deviations (SD), minimum and maximum values of each analysed trait, and ANOVA significance levels between the different origins of oil palm hybrids.

Production traits	Mean	SD	Min	Max	ANOVA
BN [nº]*	50.36	20.81	1.00	87.00	***
BW [Kg]	11.28	3.15	2.65	18.30	***
BY [Kg]*	600.14	320.35	5.30	1280.90	***
OilfM [%]	29.90	5.52	15.30	45.18	***
OildM [%]*	54.49	8.41	35.67	77.68	***
OilB [%]	19.10	4.99	5.71	32.16	***
**Oil quality traits**	**Mean**	**SD**	**Min**	**Max**	**ANOVA**
Sat [%]*	37.30	4.33	20.07	45.26	***
Mono-Un [%]*	48.95	5.92	37.46	85.46	***
Poly-Un [%]	13.53	1.64	9.93	17.33	***
OA [%]*	47.36	5.71	35.00	65.20	***
IV [cg/g]*	64.30	4.17	54.62	81.39	***
SSS [%]*	1.28	0.92	0.10	8.50	
SUS [%]*	23.69	4.39	8.93	33.20	***
SUU [%]	32.28	4.25	21.40	45.22	***
UUU [%]*	12.84	5.80	3.34	31.95	***
Tocph [ppm]*	214.47	96.51	18.30	624.20	***
Alpha [ppm]*	151.10	75.71	18.30	467.40	***
Delta [ppm]*	43.10	16.20	10.50	98.30	
Gamma [ppm]*	45.78	13.52	28.40	131.90	
Toc3 [ppm]	1149.75	367.84	306.80	2096.40	***
Alpha3 [ppm]	324.82	142.99	48.50	743.70	***
Delta3 [ppm]*	108.06	58.54	18.60	272.20	***
Gamma3 [ppm]	720.15	200.23	211.70	1199.70	***
Toc [ppm]	1366.86	423.13	392.90	2361.5	***
Car [ppm]*	795.68	241.13	353.00	1469.00	***

Significance levels: $p < 0.001$***; $p < 0.01$** and $p < 0.05$*. Traits marked with "*" did not follow a normal distributions according to Saphiro-Wilk tests. Production traits: bunch number (BN), bunch weight (BW), bunch yield (BY), oil % in fresh mesocarp (OilfM), oil % in dry mesocarp (OildM) and oil % in bunch (OilB). Quality traits: oleic acid % (OA), saturated fatty acids % (Sat), mono-unsaturated fatty acids % (Mono-Un), poly-unsaturated fatty acids % (Poly-Un), iodine value (IV), carotene contents (Car), different types of triglycerides in % (SSS, SUS, SUU, UUU), tocols (Toc), tocopherols (Tocph) and compounds Alpha, Delta and Gamma, tocotrienols (Toc3) and compounds Alpha3, Delta3 and Gamma3.

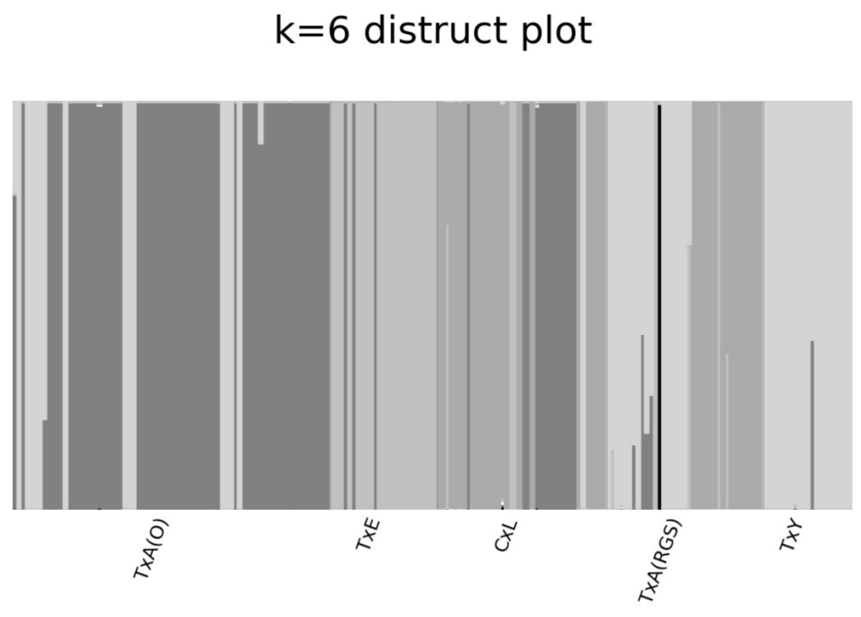

Figure S1: distruct plot of the 6 clusters used to explain our population structure.

Table S2. List of the 62 Candidate Genes (CG) targeted by single nucleotide polymorphism (SNP) which were used for the Association Mapping studies in Oil palm hybrids.

No	CG Name	GeneID_NCBI	CGpos_MPOB	CG function
1	HOLOS	NM_001304427.1	C01: 2788014-2788219	holocarboxylase synthetase
2	JC47	XM_010912944.2	C01: 5753178-5753378	Vacuolar Processing Enzyme
3	M14540	XM_010934428.1	C01: 34062235-34062416	ubiquitin-conjugating enzyme 15-like
4	CA4	XR_002166117.1	C01: 37606005-37605886	zeta-carotene desaturase
5	PKP-ALPHA	XM_010937608.1	C01: 40816787-40816570	pyruvate kinase isozyme A
6	PDHB	XM_010942881.2	C01: 51857666-51857866	pyruvate dehydrogenase E1 component subunit beta
7	SHELL	XM_010909778.2	C02: 3056550- 3056256	MADS-box transcription factor 21
8	PAT_3	NM_001319906.1	C02: 12398335-12398515	actin-3-like
9	PAT_2_ML*	XM_010914104	C02: 23775797- 23775954	actin-101
10	FFB2_C3566_S9	XM_019847934.1	C02: 31308535-31308364	transport protein Sec61 subunit alpha-like
11	CA3	XM_010914762.2	C02: 35978110-35978321	15-cis-zeta-carotene isomerase
12	HtC2_1255C2-411	XR_830848.2	C02: 43975808-43976030	ferredoxin-thioredoxin reductase catalytic chain
13	EgFATB2.2	XM_010916714.2	C03: 1846524-1846322	palmitoyl-acyl carrier protein thioesterase
14	HDAC3	XM_010916856.2	C03: 4083972-4084211	histone deacetylase 19-like
15	PO3_5-7	XM_010917092.2	C03: 7306161-7306358	NADP-dependent malic enzyme
16	MUM4	XM_010917136.2	C03: 8044490-8044684	trifunctional UDP-glucose 4,6-dehydratase/UDP-4-keto-6-deoxy-D-glucose 3,5-epimerase/UDP-4-keto-L-rhamnose-reductase RHM1
17	PO3_5-8	XM_019849509.1	C03: 8074053- 8073904	MADS-box transcription factor 3 isoform X2
18	TO3	XR_831277.2	C03: 13885380-13885529	probable tocopherol cyclase
19	DWARF7	XM_010918132.2	C03: 21298109-21298302	delta(7)-sterol-C5(6)-desaturase
20	BnC3_792	XM_010918923.1	C03: 32128327-32128119	oryzain gamma chain
21	JC59	XM_010920313.2	C04: 27102747-27102946	serine carboxypeptidase-like
22	EOCHYB	XM_010920813.1	C04: 37534421-37534541	beta-carotene 3-hydroxylase 2
23	EgDSI	AY182168.1	C04: 56149385-56149241	opsc112 protein disulphide isomerase
24	O3FAD	XM_010922542.2	C05: 4454772-4454530	omega-3 fatty acid desaturase
25	EPS3	XM_010923043.2	C05: 10242828-10242630	Peroxirredoxin 1-Cys
26	JC55	XM_010923296.2	C05: 14759213-14759438	probable ethylene response sensor 1
27	EgNAC	DQ267443.1	C05: 40852033-40852228	1 NAC protein
28	WOS6942	XM_010924566.2	C05: 40852751-40852568	NAC protein 1
29	JC8	XM_010926273.2	C06: 36747648-36747487	malonate--CoA ligase-like
30	HtC7_9200	XM_010913888.1	C06: 41269444-41269579	PP2A regulatory subunit TAP46
31	M847	XM_010927799.2	C07: 12154005-12153789	microtubule-associated protein 70-1-like
32	M3256	XR_002165148.1	C07: 12405669-12405851	T-complex protein 1 subunit delta
33	LIPOIC	XM_010927965.2	C07: 18431940-18432130	lipoyl synthase
34	FA4	XM_010928432.1	C07: 23768995-23768846	stearoyl-[acyl-carrier-protein] 9-desaturase 5
35	BnC8_761	XM_010929225.2	C08: 4351998-4351827	60S ribosomal protein L23
36	PAT_7	XM_010929278.2	C08: 5260692-5260513	fructose-bisphosphate aldolase 1
37	HtC2_11412	XM_010929998	C08: 25294193-25293999	pyrophosphate-energized vacuolar membrane proton pump

38	PAT_6	XM_010930111.2	C08: 27075380-27075546	probable plastid-lipid-associated protein 2
39	M6ASA	XM_010930131.2	C08: 27391101-27391282	Microsome localized omega-6-desaturase
40	PAT_2	XM_010932692	C09: 34724992-34725149	actin-101
41	BKACPII_1	FJ940767.1	C10: 22949664-22949486	beta-ketoacyl-ACP synthase II
42	PAT_11	XM_010936324.2	C12: 9940907-9941065	heat shock protein 83-like
43	ATAGB1	XR_002165879	C12: 17556353-17556542	GTP binding protein beta 1
44	GLUT1	AF261691	C12: 28135291-28135449	glutelin
45	ATAGB1_ML*	XM_019854409	C13: 103406- 103595	guanine nucleotide-binding protein subunit beta
46	M2200	XM_010938349.2	C13: 12503327-12503494	uncharacterized protein
47	JC35	XM_010938902.2	C13: 22806861-22807087	peptidyl-prolyl cis-trans isomerase nima-interacting 4-like
48	PAT_9	XM_010939180.2	C13: 27325349-27325185	PLAT domain-containing protein 3-like
49	QM	XM_010939750.2	C14: 5029626-5029785	60S ribosomal protein L10
50	DXS2	NM_001303573.1	C14: 21869601-21869411	1-deoxy-D-xylulose-5-phosphate synthas
51	GID1	XM_010940559.2	C14: 22469857-22470046	gibberellin receptor
52	M8373	XM_010940580.2	C14: 23021215-23021047	polyadenylate-binding protein RBP47B
53	ATP2	EU016918.1	CT: 53908-54078	ATP synthase CF1 epsilon subunit
54	atpB	EU016907.1	CT: 54576-54425	ATP synthase beta subunit (atpB)
55	PSII2	EU016919.1	CT: 75289-75438	photosystem II protein N (psbN)
56	SEQUI	XM_010906840.2	U02: 19591209-19591378	alpha-humulene synthase-like, transcript variant X2
57	FA8	XM_010906903.1	U02: 21412859-21413024	stearoyl-[acyl-carrier-protein] 9-desaturase 5
58	TO1	JN848783.1	U02: 79752127-79752276	gamma-tocopherol methyltransferase
59	M6256	XM_019846597.1	U02: 80801398-80801584	DUO pollen 3 (DUO3) gene
60	PAT_1	XM_010910419.2	U05: 40088262-40088420	5-methyltetrahydropteroyltriglutamate--homocysteine methyltransferase 1
61	ATP3	EU016883.1	U05: 50035784-50035933	ATP synthase CF0 subunit III
62	M7467	No annotation	U07: 44621406-44621548	pentatricopeptide repeat-containing protein

_ML*= Multi Locus CG. No: consecutive number of the CG; CG Name: internal name of the CG; GeneID_NCBI: identifier in the nucleotide data base of NCBI; CGpos_MPOB: CG position according to MPOB´s Oil Palm reference genome on Chromosome (Ci), unassigned Scaffold (Ui) or chloroplast gene (CT); CG function: function of the CG indicated in the nucleotide database; Amplicon primers: forward and reverse primers used for producing amplicons from each CG.

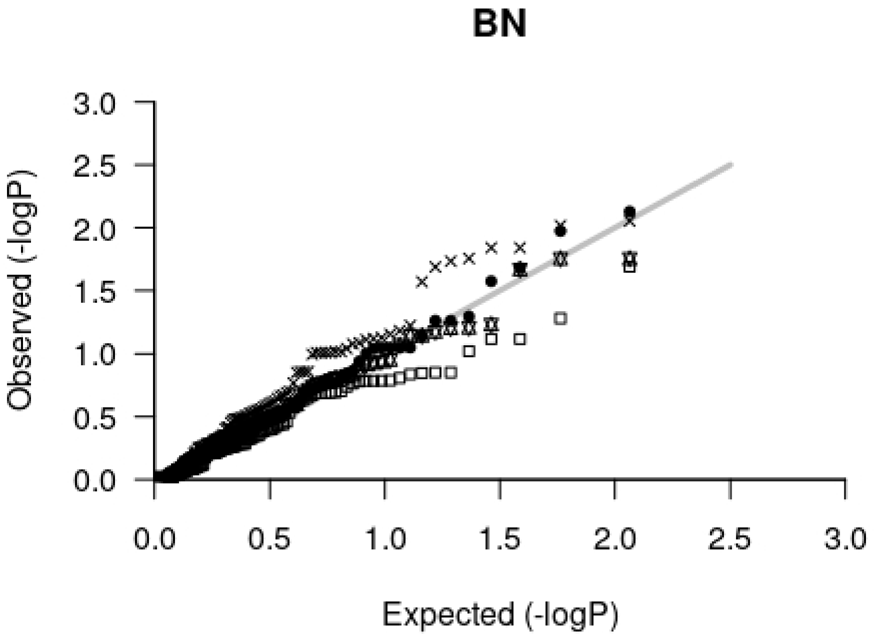
BN
3.0
2.5
2.0
1.5
1.0
0.5
0.0
Observed (-logP)
0.0
0.5
1.0
1.5
2.0
2.5
3.0
Expected (-logP)

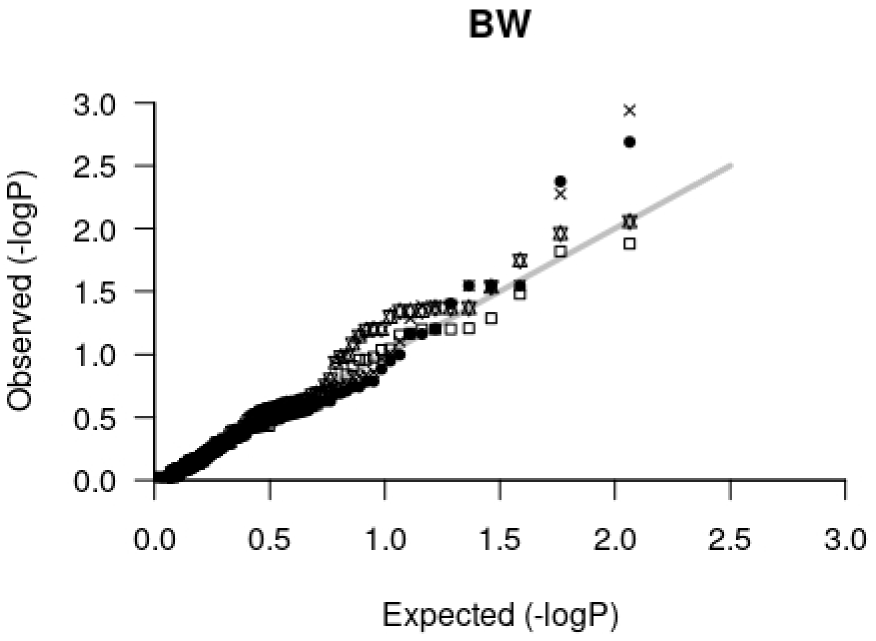
BW
3.0
2.5
2.0
1.5
1.0
0.5
0.0
Observed (-logP)
0.0
0.5
1.0
1.5
2.0
2.5
3.0
Expected (-logP)

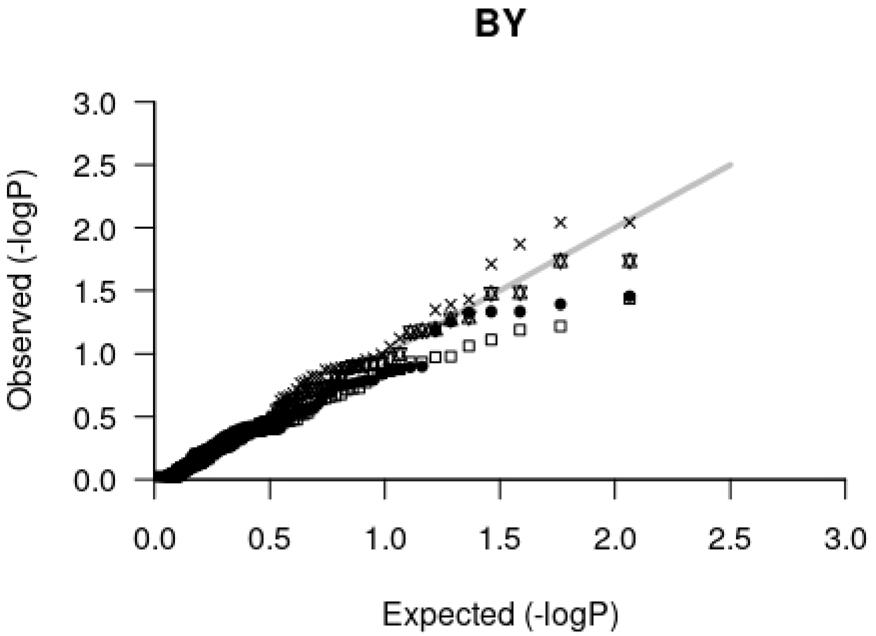
BY
Observed (-logP)
Expected (-logP)
0.0
0.5
1.0
1.5
2.0
2.5
3.0

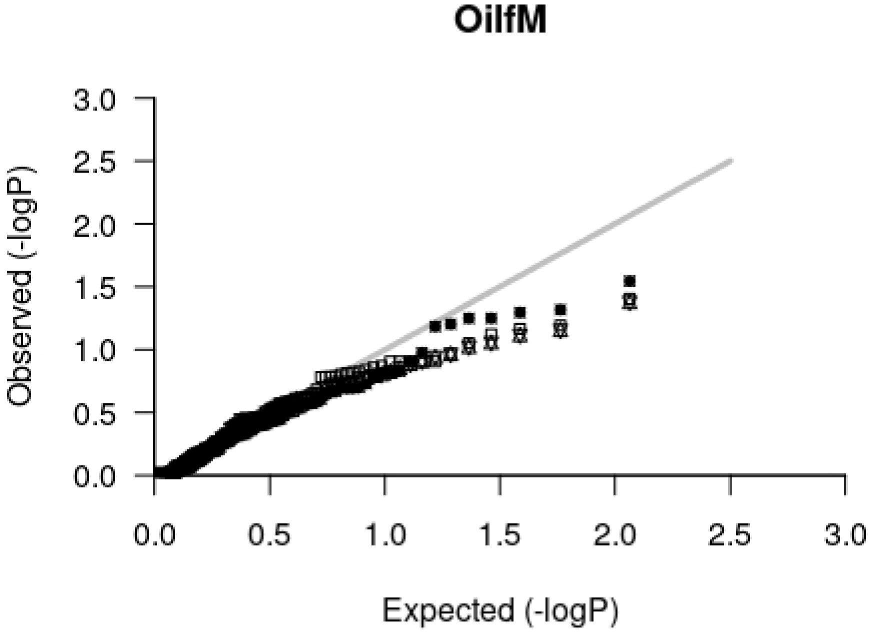
OilfM
Observed (-logP)
Expected (-logP)
0.0
0.5
1.0
1.5
2.0
2.5
3.0

OildM

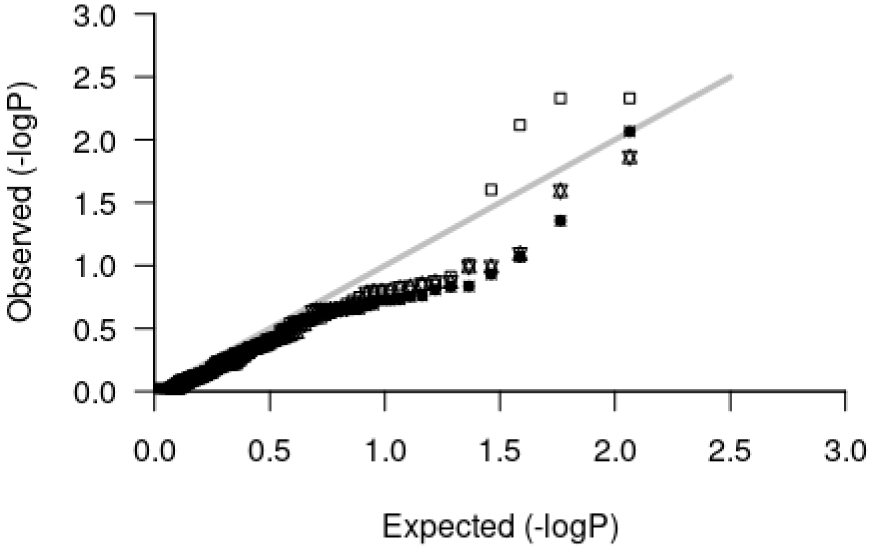
Observed (-logP)
Expected (-logP)
0.0
0.5
1.0
1.5
2.0
2.5
3.0

OilB

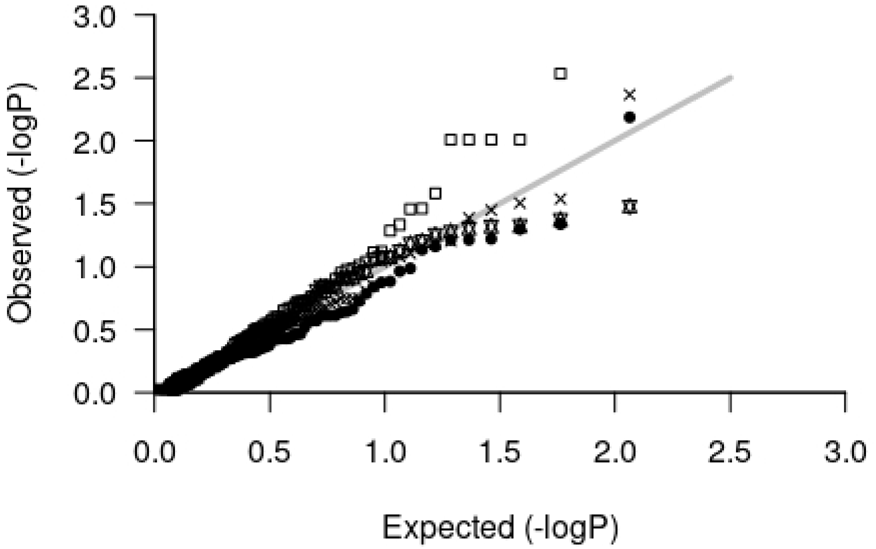
Observed (-logP)
Expected (-logP)
0.0
0.5
1.0
1.5
2.0
2.5
3.0

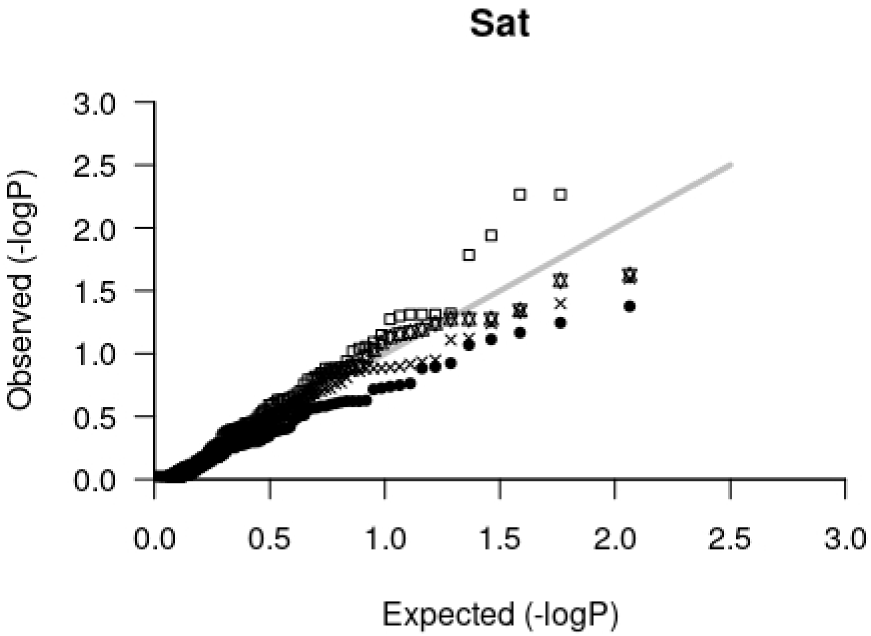
Sat
Observed (-logP)
Expected (-logP)
0.0
0.5
1.0
1.5
2.0
2.5
3.0

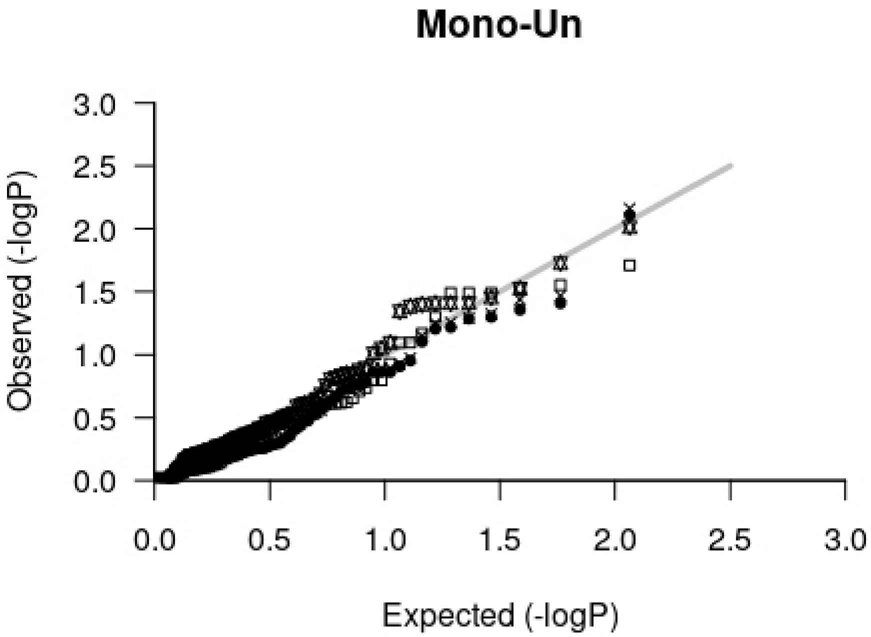
Mono-Un
Observed (-logP)
Expected (-logP)
0.0
0.5
1.0
1.5
2.0
2.5
3.0

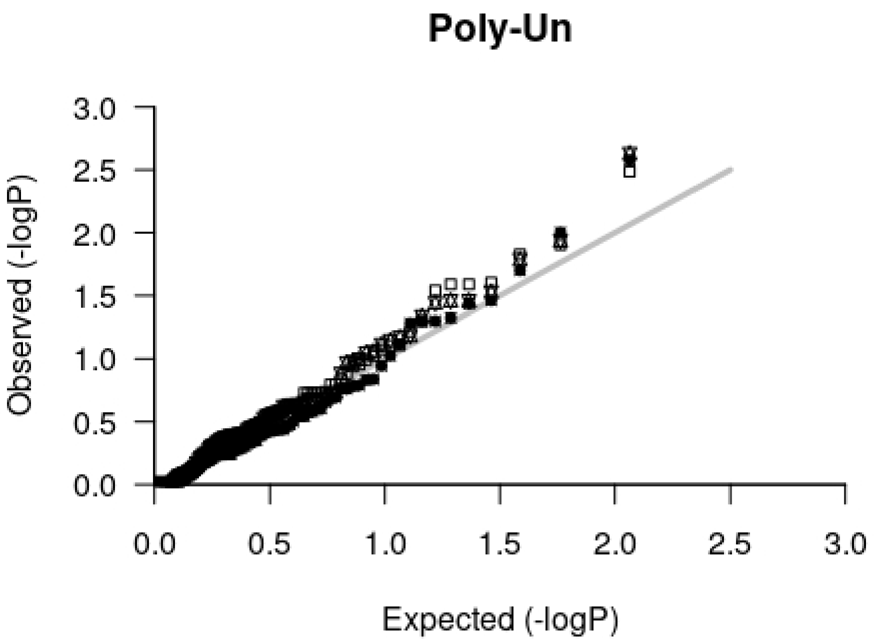

Poly-Un
3.0
2.5
2.0
1.5
1.0
0.5
0.0
Observed (-logP)
0.0
0.5
1.0
1.5
2.0
2.5
3.0
Expected (-logP)

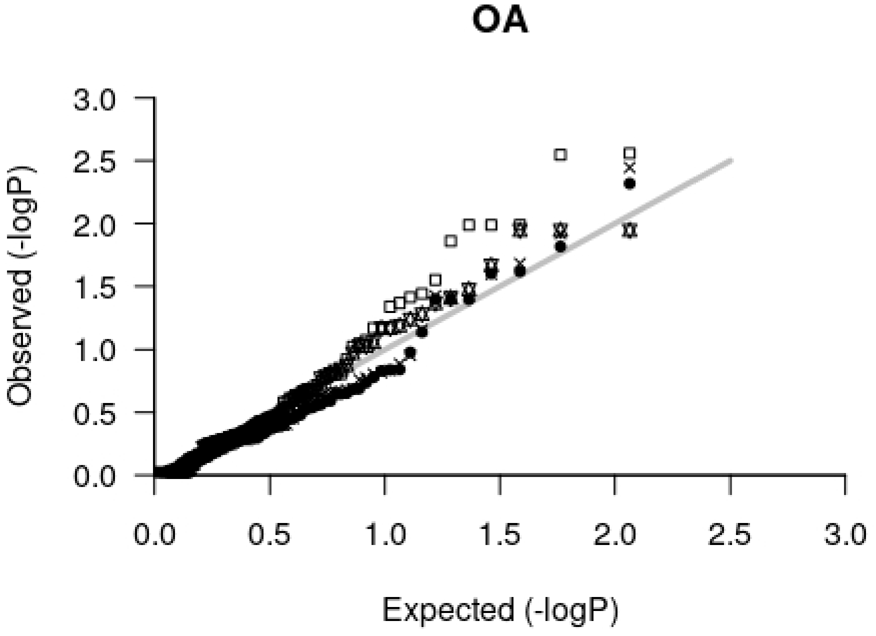

OA
3.0
2.5
2.0
1.5
1.0
0.5
0.0
Observed (-logP)
0.0
0.5
1.0
1.5
2.0
2.5
3.0
Expected (-logP)

IV

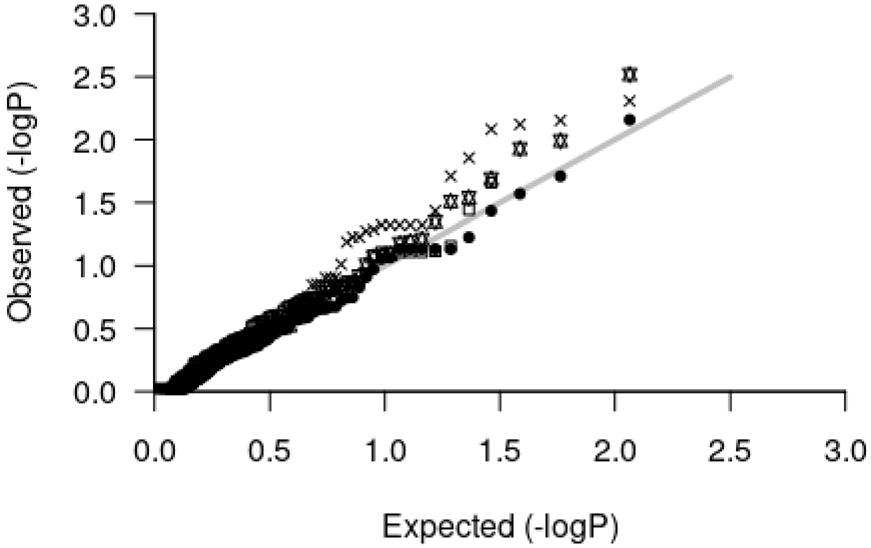
Observed (-logP)
Expected (-logP)
0.0
0.5
1.0
1.5
2.0
2.5
3.0

SSS

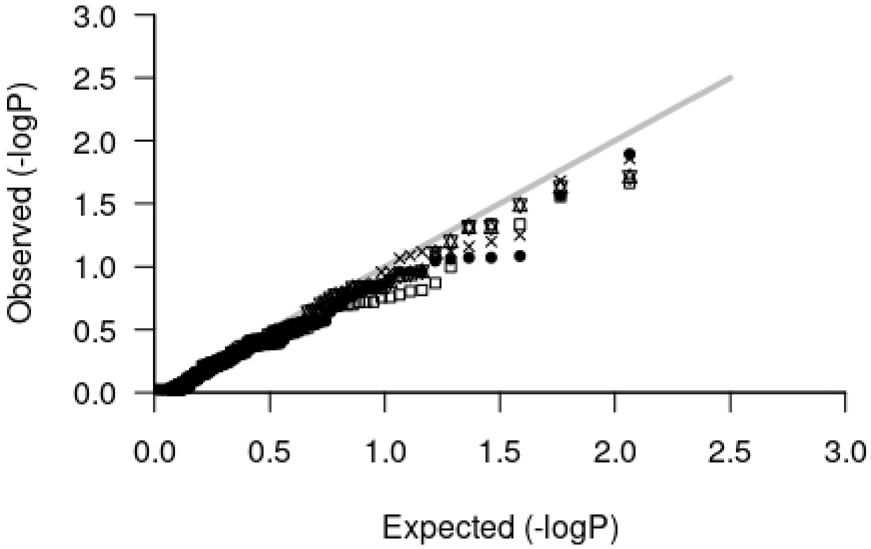
Observed (-logP)
Expected (-logP)
0.0
0.5
1.0
1.5
2.0
2.5
3.0

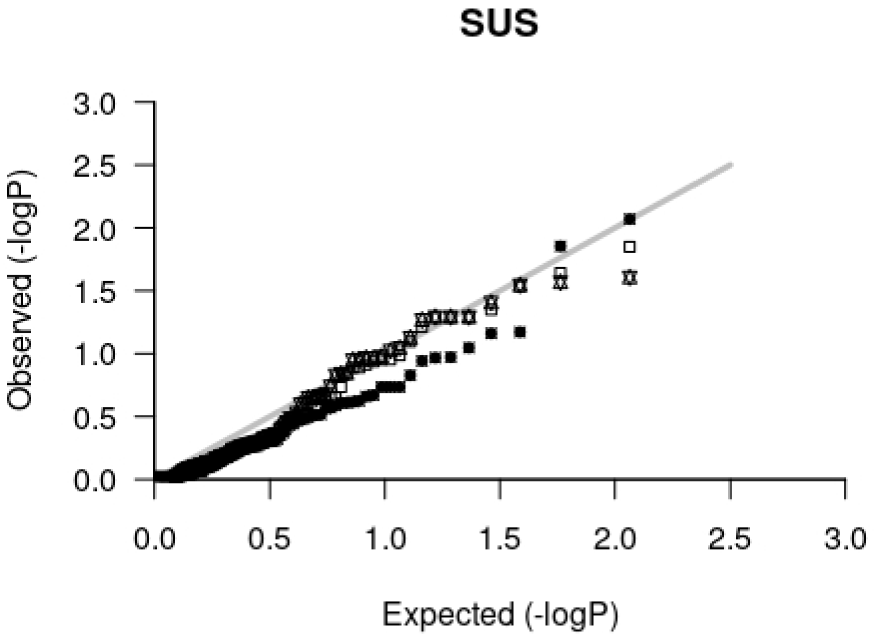
SUS
Observed (-logP)
Expected (-logP)
0.0
0.5
1.0
1.5
2.0
2.5
3.0

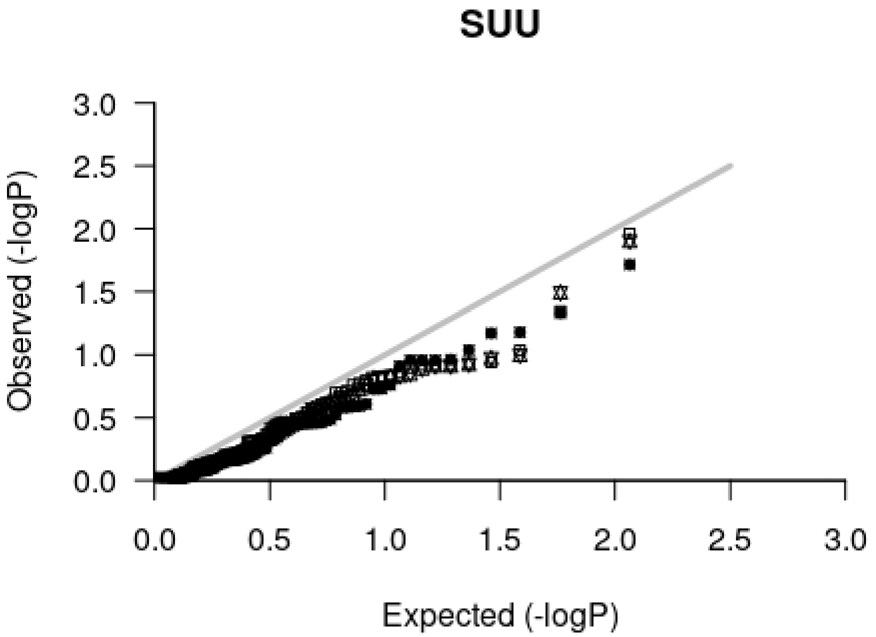
SUU
Observed (-logP)
Expected (-logP)
0.0
0.5
1.0
1.5
2.0
2.5
3.0

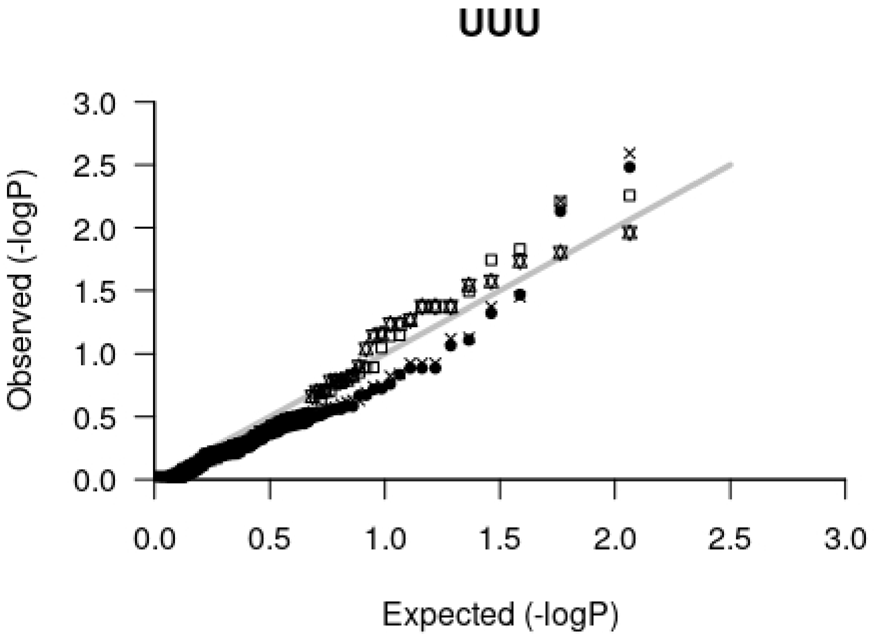
UUU
Observed (-logP)
Expected (-logP)
0.0
0.5
1.0
1.5
2.0
2.5
3.0

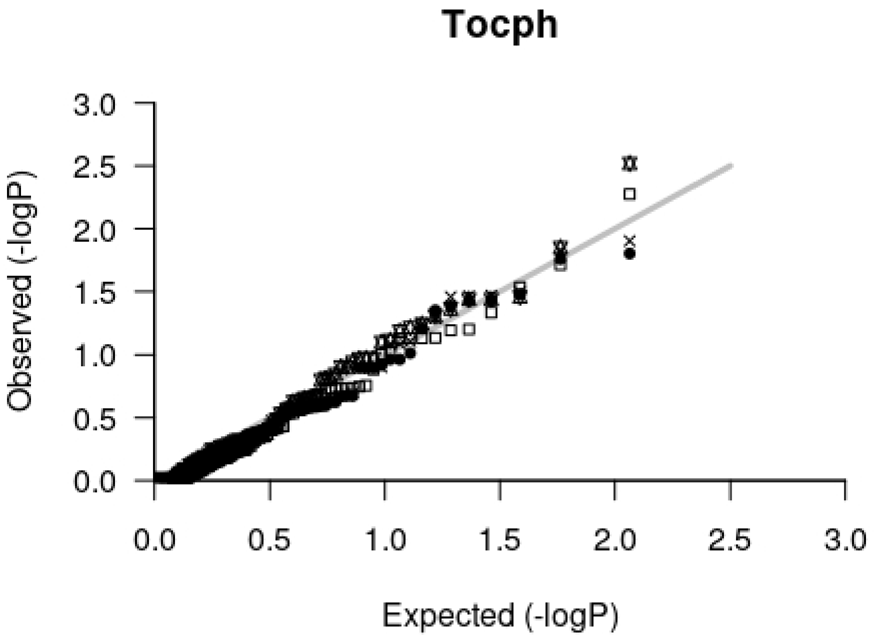
Tocph
Observed (-logP)
Expected (-logP)
0.0
0.5
1.0
1.5
2.0
2.5
3.0

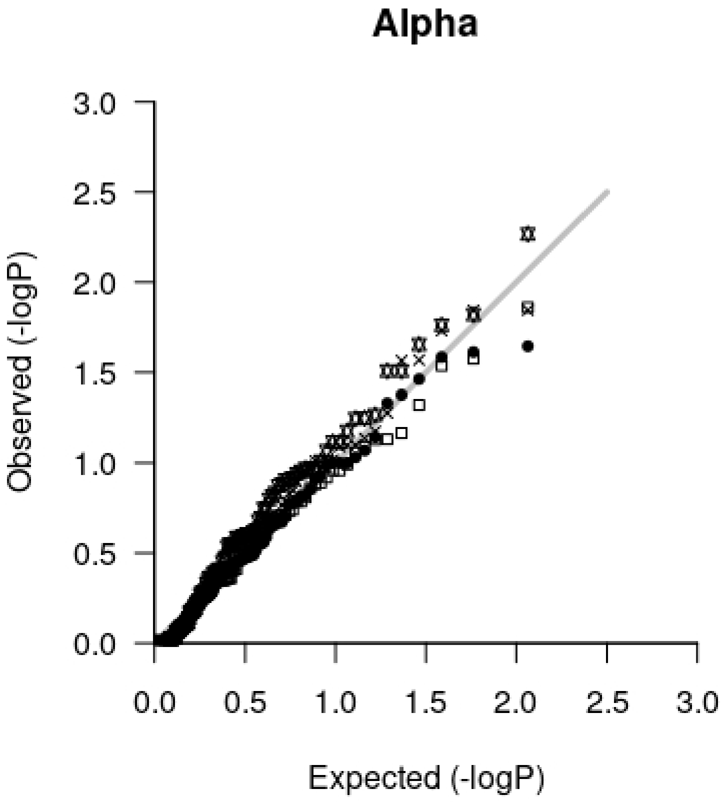
Alpha
3.0
2.5
2.0
1.5
1.0
0.5
0.0
Observed (-logP)
0.0 0.5 1.0 1.5 2.0 2.5 3.0
Expected (-logP)

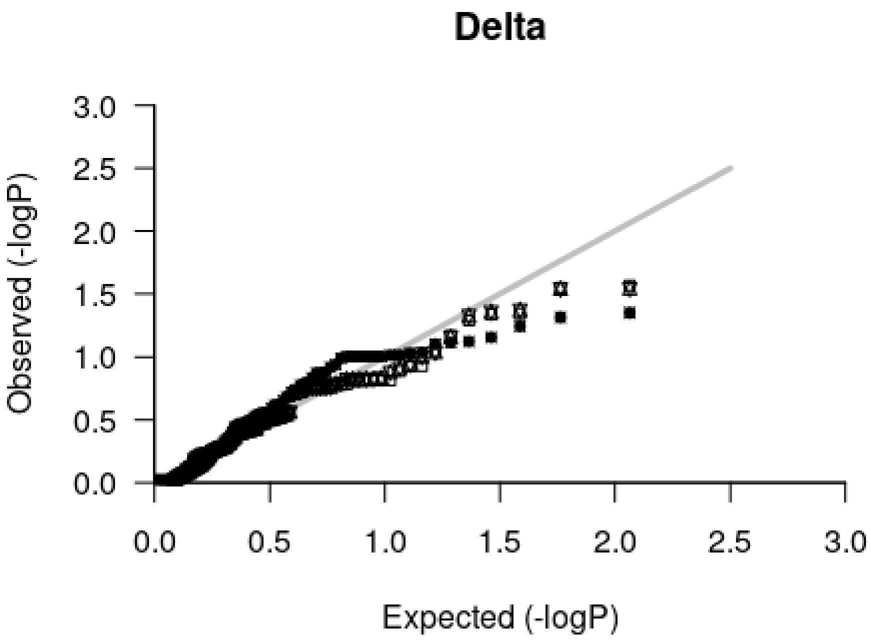
Delta
3.0
2.5
2.0
1.5
1.0
0.5
0.0
Observed (-logP)
0.0 0.5 1.0 1.5 2.0 2.5 3.0
Expected (-logP)

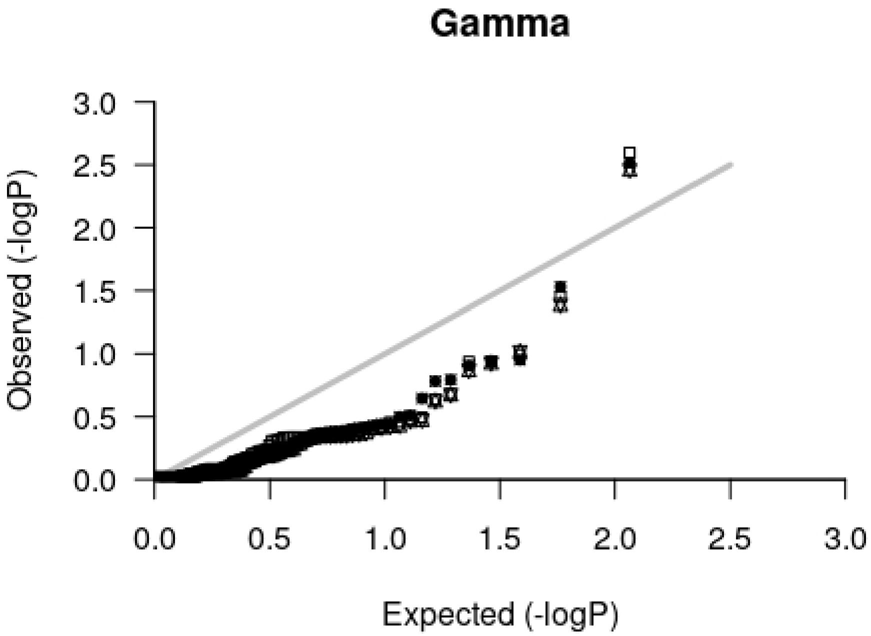
Gamma
Observed (-logP)
Expected (-logP)
0.0
0.5
1.0
1.5
2.0
2.5
3.0

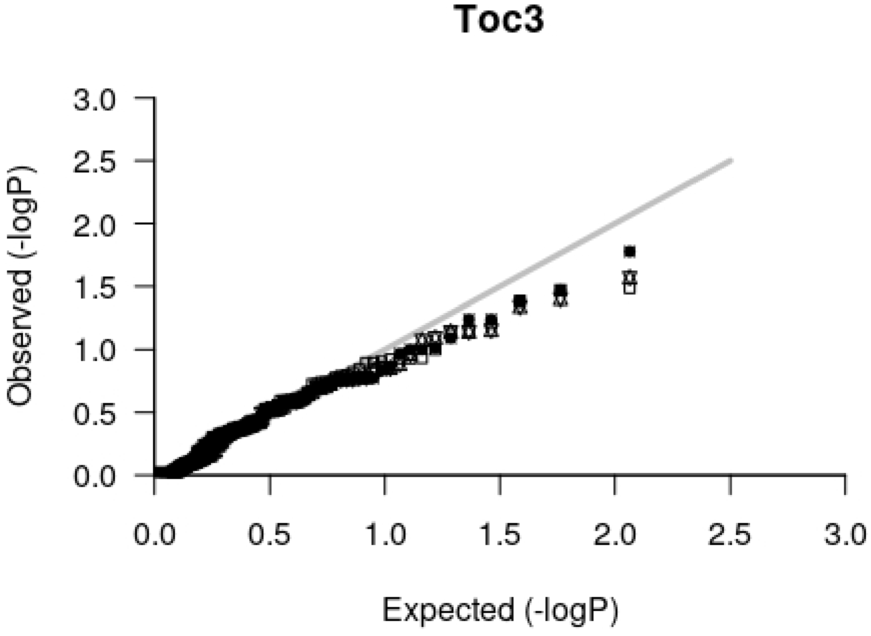
Toc3
Observed (-logP)
Expected (-logP)
0.0
0.5
1.0
1.5
2.0
2.5
3.0

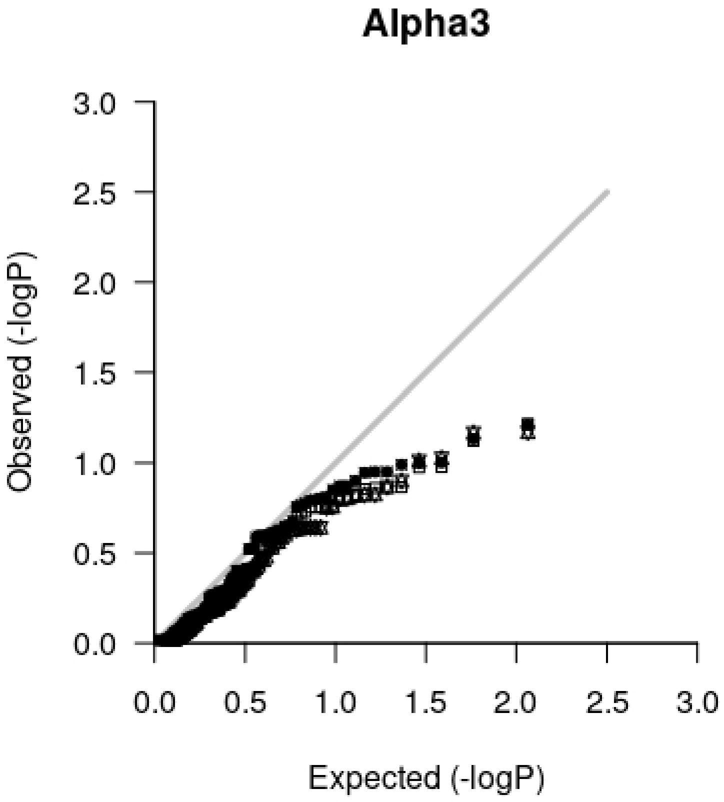
Alpha3
Observed (-logP)
Expected (-logP)
0.0
0.5
1.0
1.5
2.0
2.5
3.0

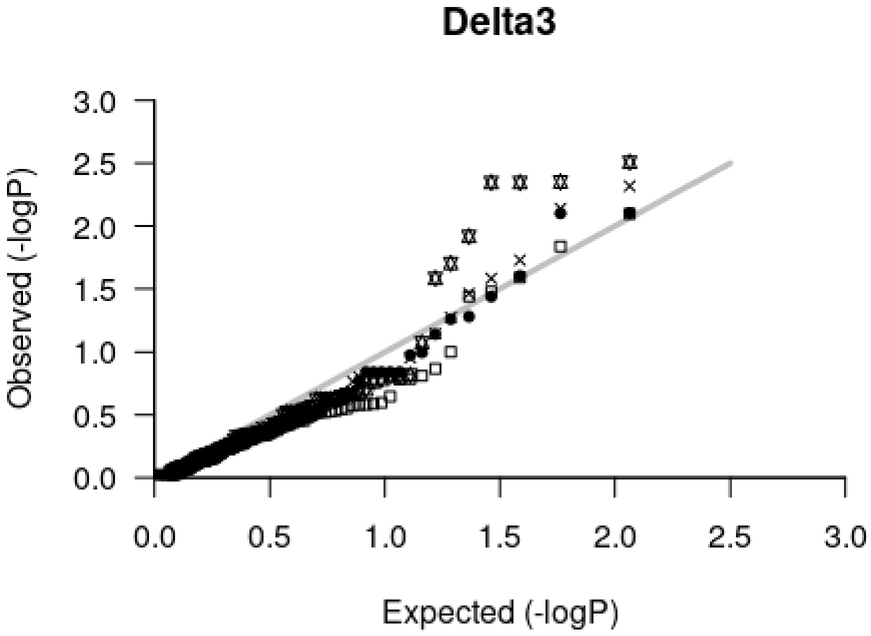
Delta3
Observed (-logP)
Expected (-logP)
0.0
0.5
1.0
1.5
2.0
2.5
3.0

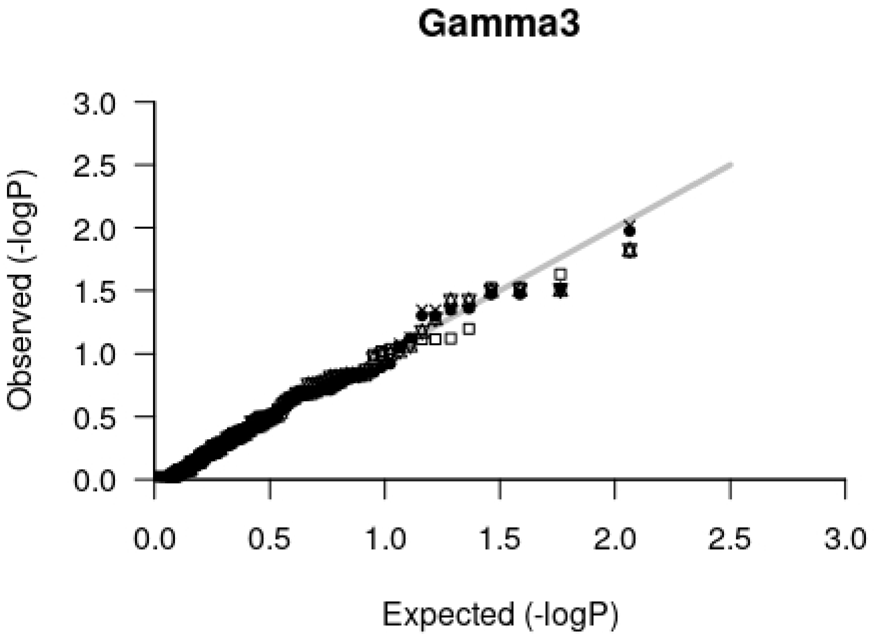

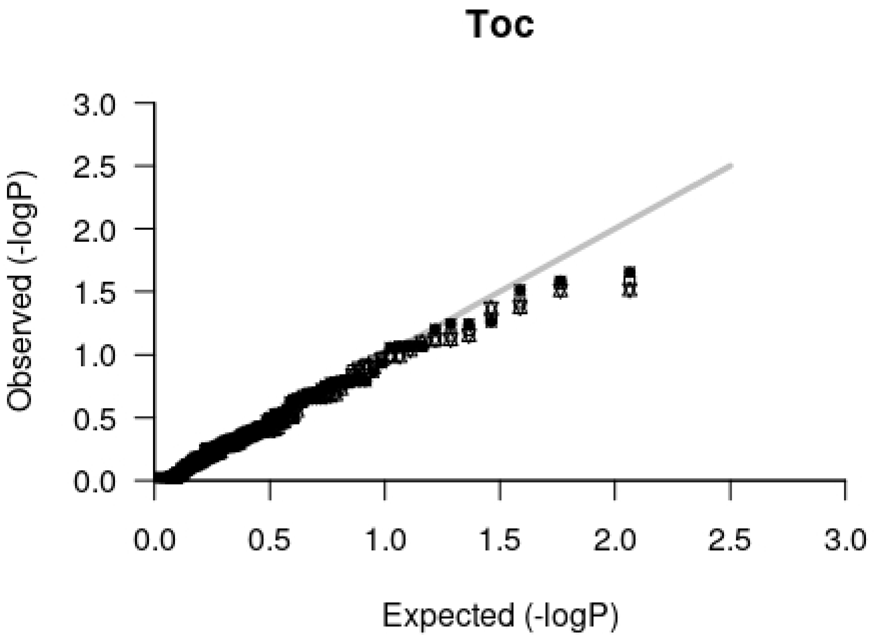

Figures S2: QuantileQuantile plots of the different studied traits for the tested models (black circles: MLM_PCA+K; white squares: MLM_Q+K; stars: GLM_Q; crosses: GLM_PCA).

Table S3: Characteristics of all 171 Candidate genes analysed initially by Amplicon sequencing in oil palm hybrids

No	CG Name	**GeneID_NCBI**	**CGpos MPOB (Amplicon_length)**	**CG function**	**Amplicon Pimers**	**Library No**
1	ACAD	XM_010942754.2	C16: 15433126-15432973 (154 bp)	peroxisomal acyl-coenzyme A oxidase 1	Fw:TCCAGCTATAAAAGGACAAGGAA<br>Rv: TTTGGGATCAAATGTTGCAG	2
2	ACYL-ACPF	XM_010926998.2	C07: 1586207-1586053 (155 bp)	palmitoyl-acyl carrier protein thioesterase	Fw: AAGCAGTGGACCCTTCTTGA<br>Rv:TCTATAGAAGCCGTCCGATCA	3
3	AG1	XM_019849509.1	C03: 8101407-8101187 (221 bp)	MADS BOX trancription factor	Fw: AGGAGGAGCCAAGAGGTAGC<br>Rv: TTGTTGGCGTATTCGTAGAGG	3
4	AIL5	XM_010930367.2	C02: 47130315-47130524 (210 bp)	AP2-like ethylene-responsive transcription factor	Fw: TCATGAACAGTGACCTCCCC<br>Rv: CTTCAAAACCCAACGCCAGA	3
5	ANT	XM_019849201.1	U10: 19445997-19446205 (209 bp)	AP2-like ethylene-responsive transcription factor	Fw: TTGGCTCTTGGTTCATTGGC<br>Rv: AGGATTCAAGTCACGGCTGA	3
6	ASP2	No annotation	C16: 3316490-3316701 (212 bp)	asparagine synthetase-related protein	Fw: GGAAAATAATATCTTGAGTTCCACA<br>Rv: TGGATCATAGATCGGA	3
7	ATAGB1	XR_002165879	C12: 17556353-17556542 (190 bp)	GTP binding protein beta 1	Fw: ATTCACTGGATTGGGCTCCT<br>Rv: CATGCACTATCAAGACCACCAC	1
8	ATP1	EU016946.1	CT: 15520-15741 (222 bp)	ATP synthase CF0 subunit IV	Fw: TTCGGAATCCACAAACCATT<br>Rv: TCGTAGGCGCAGCTAACTCT	2
9	ATP2	EU016918.1	CT: 53908-54078 (171 bp)	ATP synthase CF1 epsilon subunit	Fw: CAATGGTTAACGGTGGCTCT<br>Rv: TGCATGTCTCTTACCCTCAGC	2
10	ATP3	EU016883.1	U05: 50035784-50035933 (150 bp)	ATP synthase CF0 subunit III	Fw: TTGCTGTAGGCCAAGCTGTA<br>Rv: AAAAGGATTCGCCAACAAAA	2

11	atpB	EU016907.1	CT: 54576-54425 (152 bp)	ATP synthase beta subunit (atpB)	Fw: TGCGCAAAGAGTTAAGCAAA Rv: CCACGAAGAAGGGTTGTGAT	2
12	AUX2	XM_010920561.2	C04: 33587161-33587383 (223 bp)	probable indole-3-pyruvate monooxygenase	Fw: CAGGGTGTTCCTTTCGTGAT Rv: ACTGGTTGAATCTCGGGTTG	2
13	BAK1	XM_010938735.2	C13: 25407350-25407528 (179 bp)	somatic embryogenesis receptor kinase 2-like	Fw: ACAGCAGTCCGTGGAACAAT Rv: ACCCAGTCAAGCAACATCAC	3
14	BKACPII_1	FJ940767.1	C10: 22949664-22949486 (179 bp)	beta-ketoacyl-ACP synthase II	Fw: TGACCTTTGCATCATTCAGC Rv: ACGCAGCTTCTTTGTTGGT	1
15	BKACPIII	AF169015.1	C15: 19066681-19066878 (198 bp)	beta ketoacyl synthase III	Fw: TGCAACAGTCAAGGATGAGG Rv: AGCCAGTCAATGCTGGAACT	1
16	BnC10_7131	XM_010934039.2	C10: 22721817-22722099 (283 bp)	cell division control protein 48 homolog B isoform X2	Fw: GGCAGGCATGGTAGCTCTTA Rv: TGGCTGAAGGTGTTGTCAAG	1
17	BnC12_2975	XM_019854065.1	C12: 13275500-13275773 (274 bp)	nucleolar GTP-binding protein 2-like	Fw: ATCATCCCAGCAGCTAATGC Rv: GCAAAACATTTCCCACCTT	1
18	BnC13_gi191204957	XM_010938111.2	C13: 5330168-5330456 (289 bp)	TPD1 protein homolog 1-like	Fw: AGGACGACGTGGTGGTGTA Rv: CAGGAGACGGAGGAGACGTA	1
19	BnC2_10C3-629	XM_019847956.1	C02: 28517586-28517885 (300 bp)	MADS-box transcription factor 21 isoform X2	Fw: TGAAGCAGGACATGAGTTTGA Rv: TGCAACAAAAGTCATGCAAT	1
20	BnC2_1289	XM_010914447.1	C02: 29220936-29220683 (254 bp)	FRIGIDA-like protein 3	Fw: TAAGGTCACAGAGGCCCAAG Rv: CATTTTCAGCAGGCTTCACA	3
21	BnC3_792	XM_010918923.1	C03: 32128327-32128119 (209 bp)	oryzain gamma chain	Fw: GACTGGGAAAGGCATCTCTCT Rv: CGACAACCTTAACACCCACA	3

22	BnC7_3962	XM_010927796.2	C07: 12214023-12213768 (256 bp)	alpha,alpha-trehalose-phosphate synthase	Fw: TGTCTGTGATGCAGGAGAGG<br>Rv: TGCGAGCTTCTTACTGCTTG	1
23	BnC8_761	XM_010929225.2	C08: 4351998-4351827 (172 bp)	60S ribosomal protein L23	Fw: CAACTTGTTAGTTTGTGTTTTGGAA<br>Rv: ACAGGTTGTTCCCGAATCAG	1
24	BRI1	XM_010929461.2	C01: 2799339-2799525 (187 bp)	systemin receptor SR160-like	Fw: CTGCAAGGTTGGAGAAGAGC<br>Rv: GTGAATTATGTGGGGAATGC	2
25	CA3	XM_010914762.2	C02: 35978110-35978321 (212 bp)	15-cis-zeta-carotene isomerase	Fw: CAGATGGTTGGGCAGGTAAT<br>Rv: TGACGTCCATCAAGAATTGC	2
26	CA4	XR_002166117.1	C01: 37606005-37605886 (120 bp)	zeta-carotene desaturase	Fw: GATGTGGGATCCTGTTGCTT<br>Rv: TCAGGAGAACCCTTCAGCAT	2
27	DDB1_CUL4	XM_010913578.2	C02: 9118490-9118731 (242 bp)	DDB1-CUL4 associated factor 1	Fw: ATTCTCAGGCGAAATCCAGA<br>Rv: TTCCTCCCAACCTCAAACAG	3
28	DEF1	NM_001303583.1	U03:955283-955535 (253 bp)	MADS box transcription factor	Fw: CGAGCACCCAGTTTATGGTT<br>Rv: GCGGATAGAGAGGCTTACCA	1
29	DWARF7	XM_010918132.2	C03: 21298109-21298302 (194 bp)	delta(7)-sterol-C5(6)-desaturase	Fw: ACCTTCAAACAAAGCCATGC<br>Rv: TCCAAACTCCACAAAGACCA	1
30	DXS2	NM_001303573.1	C14: 21869601-21869411 (191 bp)	1-deoxy-D-xylulose-5-phosphate synthas	Fw: ATGTTGGTGGGAATGGAGGT<br>Rv: TCCCTTGTTCTTGCGTCTCT	3
31	EgAcp	XM_010926732.1	C06: 42406831-42406636 (196 bp)	1-aminocyclopropane-1-carboxylate oxidase 1	Fw: ACAAGGATGGAAGCCGTCTA<br>Rv: CGTCGTCGTCACTTGAACAT	3
32	EgARF1	JN003543.1	C09: 32491569-32491287 (283 bp)	Auxin response factor as transcription factor	Fw: AGCCCTTGATTTGTCGATGC<br>Rv: ACTGGACTTATCTTGGGGTTGT	3
33	EgBRX	XM_019853109.1	C10: 23849149-23848990	Brevis radix for regulating brassinosteroid-biosynthesis	Fw: ATGTGTAACCCATCGCTCCA<br>Rv: ACTCTTTCGGGCCTTGGTTA	3

			(160 bp)			
34	EgDSI	AY182168.1	C04: 56149385-56149241 (145 bp)	opsc112 protein disulphide isomerase	Fw: GTGCGGATAGTCAGGAGCTT<br>Rv: GCCTCCTTCTTCAGCAACAC	3
35	EgEBF	XM_010908977.2	U03: 51006442-51006615 (174 bp)	EIN3-binding F-box protein 1-like	Fw: TGCTGCCCAAAGTTGAAGTC<br>Rv: AGCAACAGCCTTCCCATAGT	3
36	EgFATB1.2	XM_010926998.2	C07: 1581809-1581616 (194 bp)	palmitoyl-acyl carrier protein thioesterase, chloroplastic	Fw: CTAATGACTGCACTGGTGGC<br>Rv: CCTGCCCCAATTGAGAATGG	3
37	EgFATB2.2	XM_010916714.2	C03: 1846524-1846322 (203 bp)	palmitoyl-acyl carrier protein thioesterase	Fw: GGAATGTGGGACCGAACTTG<br>Rv: CTCCATCCATCCTAGCACGT	1
38	EgMBAGL2-3	XM_010914716.2	C02: 33901209-33901053 (157 bp)	agamous-like MADS-box protein AGL9 homolog (AGL2-3)	Fw: CTGGGCCTAGCGTGAGTAAT<br>Rv: AACAGTTGCCAATTACAGACCA	1
39	EgNAC	DQ267443.1	C05: 40852033-40852228 (196 bp)	1 NAC protein	Fw: CGTTGTTCGGGCTAAAAGAG<br>Rv: ACTTGGTGCCTTCCCAGAGTA	1
40	EgPINF3-6_PIN1	XR_002165602.1	C10: 11001094-11000825 (270 bp)	probable auxin efflux carrier component 1c	Fw: CTACCGAGCTCCTCCCAAAA<br>Rv: GCAGGCATTTCAACATCCCA	3
41	EgPINF3-9_PIN4	XM_010910943.2	U06: 37424604-37424811 (208 bp)	probable auxin efflux carrier component 1c	Fw: ATGGAGCGAGAGGACTTCAG<br>Rv: TGAGGCTGGAGTAGGTGTTG	3
42	EgPPGL	XR_833625.1	C12: 8353843-8353638 (206 bp)	opsc155 putative 6-phosphogluconolactonase	Fw: GTGCTGTGCACAAGGCTCTA<br>Rv: GCTAGTCCCGCAGAAAGTTG	1
43	EgTPase	AJ507416.1	C03: 36154746-36154995 (250 bp)	partial TPase pseudogene	Fw: TAGGCCTCATTTGCACTCGA<br>Rv: GGCACCATACCTCGAAAAGC	3
44	EgWRI1-2.2	XM_010924633.2	C05: 39784587-39784821 (235 bp)	ethylene-responsive transcription factor WRI1	Fw: ACCAACTTTGCTAGCAGTGC<br>Rv: GGCCATCATCAATTGGGACA	3

45	EIN4	XM_019850079.1	C04: 31379185-31379020 (166 bp)	ethylene receptor 2-like	Fw: AGGTGGGACACCAGAGATTG<br>Rv: CTGCGATTCCTCCAAAACT	2
46	ELO2	XM_010939372.2	C14: 1363060-1363273 (214 bp)	elongator complex protein 1	Fw: ATCCAGCTGAGGCTGCTAAA<br>Rv: TTCGCCACTTTCTCTGTTCC	3
47	EPS134312	XM_010934072.2	C10: 22943253-22943135 (119 bp)	3-oxoacyl-[acyl-carrier-protein] synthase II	Fw: AACGAATGGACAAATTCATGC<br>Rv: TATGAGCACTCCGCATTTTG	2
48	EPS168	XM_019849021.1	C01: 43301684-43301536 (149 bp)	MADS box transcription factor	Fw: GGCGGATCGAGAACAAGATA<br>Rv: GGCGTACTCGTAGAGCTTGC	2
49	EPS3	XM_010923043.2	C05: 10242828-10242630 (199 bp)	Peroxirredoxin 1-Cys	Fw: AACATGGAGGAGGTGGTCAG<br>Rv: TTGTGAAGCGGAGGTAATCC	2
50	EPS50987A	U68756.1	C07: 23767648-23767541 (108 bp)	Esteroil-ACP- desaturase	Fw: CCAGGATCAGGAGGATTGAA<br>Rv: TTGGTCATGCTCAGAGTTGC	2
51	ETR1	XM_010923296.2	C05: 14758531-14758659 (129 bp)	probable ethylene response sensor 1	Fw: ACAATGGCCTGCTGAAGAAC<br>Rv: AGATGGGTTGCTCCACAAAG	2
52	ETR2	XM_010937466.2	C12: 25045240-25045404 (165 bp)	ethylene receptor 3	Fw: GATCCCGCAACTTCTGAGAG<br>Rv: AAGATGGTATGCCGGTCAAG	2
53	FA1	XM_010923348.2	C05: 16828712-16828892 (181 bp)	glyoxysomal fatty acid beta-oxidation multifunctional protein	Fw: TTGTCCCTCCAACTGATGTG<br>Rv: ATCCGATTCACAGCAAAACC	2
54	FA2	XM_010906903.1	C09: 6276571-6276327 (245 bp)	stearoyl-[acyl-carrier-protein] 9-desaturase 5	Fw: ATGAGAAGCGCCATGAGACT<br>Rv: GTTCCACCTTCGGACAAGAA	2
55	FA4	XM_010928432.1	C07: 23768995-23768846 (150 bp)	stearoyl-[acyl-carrier-protein] 9-desaturase 5	Fw: GTGCATCTGAAGCCTGTTGA<br>Rv: CATATCTCCAACCAAGCAAACA	2

56	FA6	XM_010928432.1	C07: 23768994-23768762 (233 bp)	stearoyl-[acyl-carrier-protein] 9-desaturase 5	Fw: TGCATTTGAAGCCTGTTGAG<br>Rv: AGTAAGGCTTGCCCCTGTCT	2
57	FA8	XM_010906903.1	U02: 21412859-21413024 (166 bp)	stearoyl-[acyl-carrier-protein] 9-desaturase 5	Fw: GGGATGAAACAGGTGCAAAC<br>Rv: CCATTCCCGAACCAATTAGA	2
58	FFB1_CL1016_S1.2	XM_010925967.2	C01: 21685911-21685668 (244 bp)	2-hydroxyacyl-CoA lyase	Fw: CCCCAACTTCTTTTGTTCCA<br>Rv: TGGAATGGAGAAACTGCAAA	3
59	FFB11_C1_S1	XM_010935502.2	C11: 20181476-20181728 (253 bp)	protein maternally expressed gene 5 isoform X2	Fw: GAGCATGACCGAGATTCAGC<br>Rv: CCTCCTGATCTGACCGTGTT	1
60	FFB13_C2168_S1	XM_010938902.2	C13: 22806564-22806378 (187 bp)	peptidyl-prolyl cis-trans isomerase NIMA-interacting 4	Fw: GCGAATGGAAAGCATGTGTT<br>Rv: TGGTGTCTCTTCATTACCCACA	3
61	FFB2_C2_S1	XM_010914588.1	C02: 31455509-31455742 (234 bp)	cytochrome P450 71A9-like	Fw: CTGCTTCCTCGAGAGTCCAT<br>Rv: CCCCAAATTCATTCCAGGGC	3
62	FFB2_C3566_S9	XM_019847934.1	C02: 31308535-31308364 (172 bp)	transport protein Sec61 subunit alpha-like	Fw: GGTGCACAAAAGTTACTTGGC<br>Rv: TCTGCAGAAGTTCATCCAAACA	3
63	FFB2_C4663_S1.2	XM_010913692.2	C02: 13183406-13183579 (174 bp)	tubby-like F-box protein 8 isoform X2	Fw: GCACTGCATCATGTACTCCA<br>Rv: TCAGAGAAGCGAGAACTGCT	1
64	FFB2_C4741_S3	XM_010914549.2	C02: 31047775-31048026 (252 bp)	3-oxoacyl-[acyl-carrier-protein] reductase 4-like	Fw: TAATGTAAGCTCGGGTGGCT<br>Rv: TCCCGCGTAACATAGATCGG	1
65	FFB2_C8_S1.2	XM_010914588.1	C02: 31455556-31455849 (294 bp)	cytochrome P450 71A1-like	Fw: TATCCTGCCAAACACGAGAG<br>Rv: ATTCCTGGTGCCTCGTTCAT	1
66	FFB6_C2082_S1	XM_010925974.2	C06: 33052533-33052692 (160 bp)	3-ketoacyl-CoA synthase 4	Fw: TCCTTCTTCACAGCTGCAGA<br>Rv: ATGAAGCACACATGAGGGCA	3
67	FFB6_C3684_S1	XM_019851300.1	C06:	aspartic proteinase oryzasin-1-like	Fw: ACCTGAGAGTTGGATTTGCAG	3

			33630243-33630033 (211 bp)		Rv: AACGGGCAGAACAACATACA	
68	FFB8_C1455_S3.4.5.6	XM_010929243.2	C08: 4631353-4631609 (257 bp)	E3 ubiquitin-protein ligase RGLG2-like isoform X2	Fw: AGGGCCTTGAGTTATGTCCC<br>Rv: TCCAGATTACCGCAACACCA	3
69	FFB8_C545_S1	XM_010929225.2	C08: 4354803-4354604 (200 bp)	60S ribosomal protein L23	Fw: GGGGCGAAGAACCTCTACAT<br>Rv: TCAAAGTACATATAGACGCCGTC	1
70	GID1	XM_010940559.2	C14: 22469857-22470046 (190 bp)	gibberellin receptor	Fw: CGAGTCGGAGAAGAGATTGG<br>Rv: AAGATCCAGTCCTGCCACTG	1
71	GLO2	XM_019846334.1	U02: 30414183-30414396 (214 bp)	MADS box transcription factor 2	Fw: TTGCATGCCAGATTCCAATA<br>Rv: CAGCCATCTATTAGCCCATCA	3
72	GLUT1	AF261691	C12: 28135291-28135449 (159 bp)	glutelin	Fw: TTCCAATCCTTCCAGCAATC<br>Rv: AGTATCGGCATCGGTGTAGC	1
73	HDAC3	XM_010916856.2	C03: 4083972-4084211 (240 bp)	histone deacetylase 19-like	Fw: TCCTGGAACGGGAGACATAC<br>Rv: ACACACTCTGCATGGCCTTT	3
74	HOLOS	NM_001304427.1	C01: 2788014-2788219 (206 bp)	holocarboxylase synthetase	Fw: GCATCCACATGTCCAAACTG<br>Rv: CATAATATCCGCTCCCCTGA	2
75	HtC10_11102	XM_010934308.2	C10: 26439083-26439382 (300 bp)	uncharacterized LOC105053217	Fw: CCATTGATACCTAAAGCTGGAGA<br>Rv: GGACATGACCAAGCTTGAAA	3
76	HtC2_11412	XM_010929998	C08: 25294193-25293999 (195 bp)	pyrophosphate-energized vacuolar membrane proton pump	Fw: AGGCAGTTCAACACCATTCC<br>Rv: AACAAGAGAGCCTGCAAGGA	1
77	HtC2_1255C2-411	XR_830848.2	C02: 43975808-43976030 (223 bp)	ferredoxin-thioredoxin reductase catalytic chain	Fw: AGGCAAACTTGGAGCACAGA<br>Rv: TCAGAAATGTTTCCCGCATC	1
78	HtC2_7081	XM_010915183.2	C02: 44068212-44067983	peroxidase 63	Fw: GGAGGTGCGACAACTTCAAT<br>Rv: TGTGGTGACAAACTGCAGAA	3

			(229 bp)			
79	HtC4_2106	XM_010919763.2	C04: 4078977-4079257 (281 bp)	3-isopropylmalate dehydratase large subunit	Fw: CCATATACAGCTGCGGCTTC<br>Rv: ACCCTACAGTCCCCACCTCT	1
80	HtC4_240	XM_010920216.1	C04: 24573578-24573296 (283 bp)	membrane steroid-binding protein 2	Fw: AGACCATGGCGCTAAATCAG<br>Rv: CCTCCCCTCATCTGAAAAGA	3
81	HtC4_4489	XM_010919778.2	C04: 4462811-4462530 (282 bp)	probable methyltransferase PMT21	Fw: ATCCTGCGACCAAATGGATA<br>Rv: TGCACTGAATTGCATATTTCC	1
82	HtC7_9200	XM_010913888.1	C06: 41269444-41269579 (136 bp)	PP2A regulatory subunit TAP46	Fw: TGCAACTTTTGCTCAGGATG<br>Rv: TGCCAATCTTTCCCTCTCAC	1
83	HtC8_1026C1-144	XM_010929898.1	C08: 23228408-23228684 (277 bp)	GPI-anchored protein LLG1	Fw: CTCTGCTGTGCTGCTCTCAC<br>Rv: ATGCCAGAACCATTCCAGAT	1
84	HtC8_11217	XM_010929897.2	C08: 23297262-23297520 (259 bp)	uncharacterized WD repeat-containing protein C2A9.03-like isoform X2	Fw: CCTCTCCAAATCCATTGCTG<br>Rv: GCGGCTGTATTGAAGGAGAC	1
85	IA2	XM_010925757.2	C06: 27105094-27104931 (164 bp)	jasmonic acid-amido synthetase JAR1	Fw: GCAGGCGGAAGCTAATACTG<br>Rv: GATCACGTAGTGGCCTGGAT	2
86	JC19	XM_010922207.2	C05: 513397-513194 (204 bp)	Tropinone reductase	Fw:TGCTGGGACAAATATAAGGAAAA<br>Rv: GTCGCTGCATAAATGGTTCC	1
87	JC35	XM_010938902.2	C13: 22806861-22807087 (227 bp)	peptidyl-prolyl cis-trans isomerase nima-interacting 4-like	Fw: CCATGTGGGGTAAATCCTTG<br>Rv: GAATGCCCATCAGGAAAGAA	2
88	JC41	XM_010933280.1	C10: 11707966-11707723 (244 bp)	zerumbone synthase-like	Fw: AGAAAGCACGGTGCAAAGAT<br>Rv: ATTCATTGAAGTCGGCATCC	1
89	JC47	XM_010912944.2	C01: 5753178-5753378 (201 bp)	Vacuolar Processing Enzyme	Fw: CATCAGGGCCACTATCAACC<br>Rv: TTGTGCCCCCTTTTTGTTAG	2

90	JC55	XM_010923296.2	C05: 14759213-14759438 (226 bp)	probable ethylene response sensor 1	Fw: CTTTGTGGAGCAACCCATCT<br>Rv: GTGTCCGTATCAAGCCCATT	1
91	JC59	XM_010920313.2	C04: 27102747-27102946 (200 bp)	serine carboxypeptidase-like	Fw: GACATTAGGAAGCAGTGCGAAG<br>Rv: CCATCCTCAAGAAGAGCAGGA	2
92	JC8	XM_010926273.2	C06: 36747648-36747487 (162 bp)	malonate--CoA ligase-like	Fw: AGGGCACTGATGGAATGAGA<br>Rv: TCATCACATGTAGAAGCTCTGC	2
93	LCY-B	XM_010944289.2	U01: 25121988-25122248 (261 bp)	lycopene-b-cyclase	Fw: TGGAAGAATCTGTGGCCCAT<br>Rv: AACCAAAGGAAGCGTACCCT	3
94	LF_a	XM_010910529.2	C09: 30683275-30683428 (154 bp)	lipase-like PAD4	Fw: GCAGTCCGAATTTGAATGGT<br>Rv: TTTTGGAGTCCTTCCTCGAA	2
95	LIPOIC	XM_010927965.2	C07: 18431940-18432130 (191 bp)	lipoyl synthase	Fw: AATATGCTCCTCCGGGAACT<br>Rv: GTCGAATGCTTCTGGGGTAA	1
96	M14540	XM_010934428.1	C01: 34062235-34062416 (182 bp)	ubiquitin-conjugating enzyme 15-like	Fw: TGAGCTTCACAAGCTTTTCGT<br>Rv: TGTGACACAGGAGTATTAGGTGA	3
97	M2200	XM_010938349.2	C13: 12503327-12503494 (168 bp)	uncharacterized protein	Fw: AGGATCACCAATGCCTACAA<br>Rv: AGCAAGTATGTTGGCACTTCA	2
98	M2252	XR_002165175.1	C07: 12193406-12193211 (196 bp)	two-component response regulator ORR21	Fw: CTTGCATTGAGGCTTGTTGA<br>Rv: AATTCTTTGTCTGGCGTTGG	3
99	M23551	XM_010937402.2	C12: 25645701-25645899 (199 bp)	mannan synthase 1-like isoform X1	Fw: TACGGCATCGTTCTCATCAA<br>Rv: TGGTGGAGTTGGAGTGTCAG	3
100	M3117	XM_010925967.2	C01: 21688440-21688636 (197 bp)	2-hydroxyacyl-CoA lyase-like	Fw: GCCTCGGATAAAACCCTAGC<br>Rv: GATCAGATGGCTGCTGTGAA	3
101	M3256	XR_002165148.1	C07:	T-complex protein 1 subunit delta	Fw: ATCCCAGCACTGATCTCACC	1

			12405669-12405851 (183 bp)		Rv: TGTCAAGGCAACTAGGAGCA	
102	M3609	XM_010938346.2	C13: 12827505-12827646 (142 bp)	glycosyltransferase, putative	Fw: GCAGAAATCAGGGTGACCTC<br>Rv: CACGACGGAGATTGTTGTGA	2
103	M43144	XM_010917491.2	C03: 12945319-12945139 (181 bp)	GTPase Der	Fw: GGGAAGGCACAACTTTCAGA<br>Rv: TTTTGTCTGATCGGGCTCTC	3
104	M43696	XM_010928111.2	C07: 16118939-16118778 (162 bp)	ethylene-responsive transcription factor ERF014-like	Fw: TATGATCGGGAGGGTGGTAG<br>Rv: GGAGTGAGGATGAGGAGCTG	3
105	M43898	XM_010926282.2	C06: 36855343-36855184 (160 bp)	protein tesmin/TSO1-like CXC 5-like	Fw: TACAGGGCATTTCCATGGTC<br>Rv: GATGCCATTGCAAGAAGTCA	2
106	M4585	XR_831108.2	C03: 5078622-5078468 (155 bp)	DEAD-box ATP-dependent RNA helicase 20	Fw: GGAGCACCTCGACCCATT<br>Rv: CGCATTGGTCGAACAGGAAG	2
107	M4883	XM_019850224.1	C04: 4188649-4188886 (238 bp)	protein enchanced disease resistance 2	Fw: TCCTGTTCAGGCTCTGGAAT<br>Rv: CAGGGTTGCGTGAATGGTT	3
108	M6256	XM_019846597.1	U02: 80801398-80801584 (187 bp)	DUO pollen 3 (DUO3) gene	Fw: TCCTCCTGACCTGTCTGTCC<br>Rv: GTATCTGCAAACGCACGAGA	2
109	M6ASA	XM_010930131.2	C08: 27391101-27391282 (182 bp)	Microsome localized omega-6-desaturase	Fw: AAGACGCCCTTCTTCTCTCC<br>Rv: GCGCACCACCTCTTCTCTAC	1
110	M7467	XR_003388267.1 (Date palm)	U07: 44621406-44621548 (143 bp)	pentatricopeptide repeat-containing protein	Fw: TACTTGACATGGTGCGTGGTT<br>Rv: CCTCCCTTGATTGCTTCATC	1
111	M8373	XM_010940580.2	C14: 23021215-23021047 (169 bp)	polyadenylate-binding protein RBP47B	Fw: CGCCCAGTATTTGGATCAGT<br>Rv: ATTGGGCCTCTTTTGGTCTT	2
112	M847	XM_010927799.2	C07: 12154005-12153789	microtubule-associated protein 70-1-like	Fw: TGACCTAGTGCCACCATCAA<br>Rv: TAGCAACCGCACCAATGTAG	1

			(217 bp)			
113	M9861	XM_010910705.2	U05: 84508671-84508856 (186 bp)	endoglucanase 10	Fw: GCAATACCGGGAGTGGTAGA<br>Rv: TGGTGCAAGGACACTTTTCA	2
114	MADS11-1	XM_019851612.1	C07: 16866616-16866465 (152 bp)	MADS-box protein JOINTLESS	Fw: GGCGAGGGAGAAGATTCAG<br>Rv: CGGTGGAGGAGAAGATGATG	3
115	mEg3275	XM_010912620.2	C02: 7612917-7613050 (134 bp)	serine hydroxymethyltransferase 7-like	Fw: GAAGCCTGAGACCGCATAGA<br>Rv: TTCGGTGATGAAGATTGAAG	2
116	MUM4	XM_010917136.2	C03: 8044490-8044684 (195 bp)	trifunctional UDP-glucose 4,6-dehydratase/UDP-4-keto-6-deoxy-D-glucose 3,5-epimerase/UDP-4-keto-L-rhamnose-reductase RHM1	Fw: CACCGTGGAGAAGTTGGACAT<br>Rv: CTCTGACCACCCCAAATTCT	1
117	O3FAD	XM_010922542.2	C05: 4454772-4454530 (243 bp)	omega-3 fatty acid desaturase	Fw: AAGTAGCGGGGAGGAGAGAG<br>Rv: ACCAAACAAAAAGCCCTCCT	2
118	OLEOSIN	XM_010935827.1	C11: 23477579-23477749 (171 bp)	oleosin 16 kDa-like	Fw: AATCTCCCCTCGCTTCACTT<br>Rv: CAACACTCACGCTACGAGGA	3
119	OLEOYL	XM_010926998.2	C07: 1586207-1586053 (155 bp)	palmitoyl-acyl carrier protein thioesterase	Fw: AAGCAGTGGACCCTTCTTGA<br>Rv: TCTATAGAAGCCGTCCGATCA	1
120	PACT	DQ422858.1	C07: 1586069-1586260 (192 bp)	palmitoyl-ACP thioesterase	Fw: GATCAGCCCCGATCTCATAC<br>Rv: CTGACTGGAGCGTGCTTCTT	1
121	PAT_1	XM_010910419.2	U05: 40088262-40088420 (159 bp)	5-methyltetrahydropteroyltriglutamate--homocysteine methyltransferase 1	Fw: GCGAGGGAGTGAAATATGGT<br>Rv: GTCTTGAGCCCACAGTCAGG	1
122	PAT_11	XM_010936324.2	C12: 9940907-9941065 (159 bp)	heat shock protein 83-like	Fw: GGTGGATGCTATCGACGAGT<br>Rv: ACCTTGCATAGGCTCTCGAA	1
123	PAT_12	XM_010940376.2	C14: 11183666-11183860	nascent polypeptide-associated complex subunit alpha-like protein 1	Fw: GCAAAGATCGAGGACCTGAG<br>Rv: CTTGGACCTCGAAACTCCAG	1

			(195 bp)			
124	PAT_13	XM_010940239.2	C14: 14557627-14557473 (155 bp)	coatomer subunit epsilon-1	Fw: CCGACCACCTCTTCAATCTC Rv: GACCTGGTAGGAGCCAAGG	1
125	PAT_14	XM_010943249.2	C07: 36442648-36442806 (159 bp)	60S acidic ribosomal protein P0	Fw: CAAGGTTGGCTCTTCTGAGG Rv: TCCAGCTGCAAACTTCTCAA	2
126	PAT_15	XM_010930830.2	C08: 36345443-36345620 (178 bp)	temperature-induced lipocalin-1	Fw: CCAAGAACGGGGAGAACAC Rv: AAGAAGGGTGGCACGTAGAA	2
127	PAT_2	XM_010932692	C09: 34724992-34725149 (158 bp)	actin-101	Fw: ATTCCGGTGATGGTGTGAGT Rv: CCGTTCTGCAGTGGTAGTGA	2
128	PAT_3	NM_001319906.1	C02: 12398335-12398515 (181 bp)	actin-3-like	Fw: CACTTCCTCATGCCATCCTT Rv: GCAGACTCCAATTCCTGCTC	2
129	PAT_4	XM_010936612.2	C12: 15018134-15017947 (188 bp)	caffeic acid 3-O-methyltransferase	Fw: TGTTCAATGAGGGCATGAAG Rv:AGAGATGACATGAGGAAGATCAA	1
130	PAT_6	XM_010930111.2	C08: 27075380-27075546 (167 bp)	probable plastid-lipid-associated protein 2	Fw: CTCCATCGTTTTACCCGAGA Rv: AGGACTGTGCATTGTCGTTG	2
131	PAT_7	XM_010929278.2	C08: 5260692-5260513 (180 bp)	fructose-bisphosphate aldolase 1	Fw: TCCGTGAGCTCCTCTTTTGT Rv: TAGTGCCAGCAAGTTCGATG	2
132	PAT_8	XM_010919548.2	C03: 57312509-57312669 (161 bp)	5-methyltetrahydropteroyltriglutamate--homocysteine methyltransferase 1	Fw: GATCCCATCCACAGAGGAGA Rv: GGAGGAGCTTAGCAGCAGAA	2
133	PAT_9	XM_010939180.2	C13: 27325349-27325185 (165 bp)	PLAT domain-containing protein 3-like	Fw: ATCAGGACGGGGTCCATCT Rv: GCTGAAGATGTCGAGGTTGC	3
134	PDAT_2	XM_010918834.2	C03: 29958824-29958974 (151 bp)	Phospholipid-Diacylglycerol acyltransferase	Fw: TTCCCTGTAACTGAGAAGCAAA Rv: CAGAATGCAAAATCAGAACAAAA	3

135	PDHB	XM_010942881.2	C01: 51857666-51857866 (201 bp)	pyruvate dehydrogenase E1 component subunit beta	Fw: TCACAGCGGTTGGAATCATA<br>Rv: CTCTGCCTCCTCCAGACAAC	1
136	PDS3_CH13	XM_010938924.2	C13: 22675283-22675467 (185 bp)	pre-mRNA-splicing factor clf1-like	Fw: GAACCAAAGAAGCTCGCCTG<br>Rv: CGCCTTTGCCAGCTCAATTA	1
137	PKP-ALPHA	XM_010937608.1	C01: 40816787-40816570 (218 bp)	pyruvate kinase isozyme A	Fw: ACAAGCCTGTCATTGTAGCT<br>Rv: CTTCTCCTCTCTCCACCACC	1
138	PLT2	XM_010914736.1	C09: 15548387-15548160 (228 bp)	ethylene-responsive transcription factor	Fw: GGGGCATCATGGGGAAATTC<br>Rv: CTGGGTCTGGTTTGCTTCAG	3
139	PO3_5-10	XM_010934991.2	C11: 11856922-11857078 (157 bp)	GEM-like protein 7	Fw: ACGTCGAGTGAGAATCTGGA<br>Rv: TTCCGCAGAAAGGTCATTGT	3
140	PO3_5-13	XM_010941549.1	C15: 19816511-19816313 (199 bp)	proteasome subunit alpha type-5	Fw: TCTGTTTGCCTTCTCCACCA<br>Rv: GCATTGGCGGTAGAACTCTG	3
141	PO3_5-14	XM_010941553.2	C15: 19862793-19862514 (280 bp)	1-phosphatidylinositol-3-phosphate 5-kinase FAB1A-like	Fw: AACACCTAGAGACGTGGGTG<br>Rv: ACCCCAGAAAGATTGGTCGT	3
142	PO3_5-7	XM_010917092.2	C03: 7306161-7306358 (198 bp)	NADP-dependent malic enzyme	Fw: TTGGCTAGTCATCTCCCTCG<br>Rv: CCCAATGATCAAGGGGCTCA	3
143	PO3_5-8	XM_019849509.1	C03: 8074053-8073904 (150 bp)	MADS-box transcription factor 3 isoform X2	Fw: AGATTGCTGAGAATGAGAGAGC<br>Rv: TCGTCTGCTGCTGATGAGAG	3
144	PRT6	XM_010939321.1	C14: 886973-887164 (192 bp)	E3 ubiquitin-protein ligase PRT6-like	Fw: TGCGTTCCATGTTTCCAGAA<br>Rv: ATCCAGAACAGGTCCCACAG	3
145	PSII1	EU016942.1	CT: 75549-75755 (207 bp)	photosystem II phosphoprotein (psbH)	Fw: TGGCTACACAAACCGTTGAG<br>Rv: TTCCATCCAGTAAAACGAAAGAA	2
146	PSII2	EU016919.1	CT:	photosystem II protein N (psbN)	Fw: TGGAAACAACAACCCTAGTCG	2

			75289-75438 (150 bp)		Rv:GGGAGACTCATTACTTCAACTAGTCC	
147	PSII3	EU016895.1	CT: 65546-65717 (172 bp)	photosystem II cytochrome b559 alpha subunit (psbE)	Fw: TTTGTGGAGCTCAGCATGTC Rv: TTGGTCGAGGACTTCCAAAC	2
148	PYRKIN	XM_010942455.2	C01: 50296952-50297101 (150 bp)	pyruvate kinase 1	Fw: GGATACGGTGGGTCCAGAG Rv: GCCTTTGACAATCCACTGAAA	3
149	QM	XM_010939750.2	C14: 5029626-5029785 (160 bp)	60S ribosomal protein L10	Fw: GCTCTTGAGGCTGCTCGTAT Rv: CCCTCATTCCAGTCTGAAGC	2
150	R2r3	XM_019854941	C14: 1636715-1636911 (197 bp)	myb-related protein MYBAS1-like	Fw: TGAGCTTCGAGGGATACAAGA Rv: TGGACAGGGGTAGAAAGAGAA	1
151	RAP2.2_3	XM_019854559.1	C13: 22032414-22032123 (292 bp)	ethylene-responsive transcription factor	Fw: GGCTTCATTCAGAGGACCCT Rv: ACTTGCAAGCTCTCATATCAACT	3
152	RPL10	XM_010939750.2	C14: 5029479-5029721 (243 bp)	60S ribosomal protein L10	Fw: AAGCCATACCCAAAGTCACG Rv: ATGGAAGGGATGCACTCTCA	3
153	RU1	EU016944.1	C02: 62056727-62056886 (160 bp)	ribulose bisophosphate carboxylase	Fw: CAGGGGGTATTCATGTTTGG Rv: TTCACGAGCAAGATCACGTC	2
154	SEQUI	XM_010906840.2	U02: 19591209-19591378 (170 bp)	alpha-humulene synthase-like, transcript variant X2	Fw: TCCATGGAAAGCCATATGAA Rv: TGAAAATCCAACTTTGCAAGC	2
155	SHELL	XM_010909778.2	C02: 3056550-3056256 (295 bp)	MADS-box transcription factor 21	Fw: GGATCGAGAACACCACAAGC Rv: AATTTGGCTTGGCCATAGAA	1
156	SHELL2	XM_010909778.2	C02: 3056550-3056256 (295 bp)	MADS-box transcription factor 21	Fw: TAGCAGAGAATGAGCGAGCA Rv: TCAGACAAGTCTTCTAACACACCTTT	1
157	SQUA3	AF411842.1	C15: 13726940-13726660	MADS box transcription factor (SQUA3)	Fw: AGGCACTAGTTTGCCTGCAT Rv: TTTGAGCTCCAAAGCCAACT	1

			(281 bp)			
158	TO1	JN848783.1	U02: 79752127-79752276 (150 bp)	gamma-tocopherol methyltransferase	Fw: GCACCAGGAGCCACCATTAT Rv: CACATAATCACTGGCTGAGCA	1
159	TO2	XM_019848829.1	C03: 13878917-13878801 (117 bp)	tocopherol cyclase	Fw: GGGAATACAGCACACATCCA Rv: CCATGCATACTTGCCAATGA	2
160	TO3	XR_831277.2	C03: 13885380-13885529 (150 bp)	probable tocopherol cyclase	Fw: AAGGTCTCGATCCCTGAATG Rv: ATCATCCGCACCAAGAATTT	2
161	VVuACT	XM_010939417.2	C14: 1766743-1766542 (202 bp)	actin-3	Fw: CCACAACAGCAGAACGAGAA Rv: CCACAACAGCAGAACGAGAA	3
162	Wild-type_VIR	XM_010932909.2	C01: 29321468-29321727 (260 bp)	virescens R2R3-MYB gene	Fw: TGGTCAGAAGATCAGCAATCA Rv: CAAAGCAAGTCATCCCATCC	1
163	WOS104	XM_010926998.2	C07: 1586323-1586513 (191 bp)	palmitoyl-acyl carrier protein thioesterase	Fw: TTCCCCACACCATCTTTCTC Rv: GATTCAGCTTTCAGGCCAAC	2
164	WOS6942	XM_010924566.2	C05: 40852751-40852568 (184 bp)	NAC protein 1	Fw: GTTCCCGGACTTTGACGATA Rv: AGCCATGCATGTACTGTGGA	2
165	wri1	XM_010928170.2	C07: 15188796-15188619 (178 bp)	DELLA protein SLR1-like	Fw: TGGTGAAGCAGATCTCGATG Rv: TAGGGGCAGCTCTCGTAGAA	3
166	ZCD	XR_002166117.1	C01: 37600361-37600171 (191 bp)	zeta-carotene desaturase	Fw: GGCACCCTGAGAGATTCAAA Rv: TGAACGACATGGGAAAGACA	1
167	EOCHYB	XM_010920813.1	C04: 37534421-37534541 (121 bp)	beta-carotene 3-hydroxylase 2	Fw: CAGAACCGGAGTTCGGAGAT Rv: GCTTCCTCGCGATCTTCTC	2
168	**PAT_12_ML***	XM_010923558.2	C05: 25085015-25085209 (195 bp)	nascent polypeptide-associated complex subunit alpha-like protein 1	Fw: GCAAAGATCGAGGACCTGAG Rv: CTTGGACCTCGAAACTCCAG	1

169	**HtC2_11412_ML***	XM_010915041	C02: 41981898-41982092 (195 bp)	pyrophosphate-energized vacuolar membrane proton pump-like	Fw: AGGCAGTTCAACACCATTCC<br>Rv: AACAAGAGAGCCTGCAAGGA	1
170	**PAT_2_ML***	XM_010914104	C02: 23775797-23775954 (158 bp)	actin-101	Fw: ATTCCGGTGATGGTGTGAGT<br>Rv: CCGTTCTGCAGTGGTAGTGA	2
171	**ATAGB1_ML***	XM_019854409	C13: 103406-103595 (190 bp)	guanine nucleotide-binding protein subunit beta	Fw: ATTCACTGGATTGGGCTCCT<br>Rv: CATGCACTATCAAGACCACCAC	1

_ML*= Multi Locus CG**. Legend: No**: consecutive number of the CG; **CG Name**: internal name of the CG; **GeneID_NCBI**: identifier in the nucleotide data base of NCBI; **CGpos_MPOB (Amplicon_length)**: CG position according to MPOB´s Oil Palm reference genome on Chromosome (**Ci**), unassigned Scaffold (**Ui**) or chloroplast gene (**CT**) and amplicon length; **CG function**: function of the CG indicated in the nucleotide database; **Amplicon primers**: forward and reverse primers used for producing amplicons from each CG; Library No: library number in which a particular CG was included.

Table S4: Phenotypic raw data for each trait and genotype.

Genotypes	BN	BW	BY	OilfM	OildM	OilB	Sat	Mono-Un	Poly-Un	OA	IV	SSS	SUS	SUU	UUU	Tocph	Alpha	Delta	Gamma	Toc3	Alpha3	Delta3	Gamma3	Toc
1_CxL	77.0	11.9	913.3	38.0	-	28.0	29.9	59.0	11.0	57.1	69.4	0.5	16.8	38.2	19.3	125.1	110.0	15.1	-	649.2	155.6	30.0	463.6	774.3
2_CxL	39.0	9.3	363.4	37.1	69.9	22.7	35.0	52.1	12.6	50.2	69.4	1.7	18.7	32.1	19.0	53.9	53.9	-	-	589.5	127.9	34.7	426.9	643.4
3_CxL	53.0	9.6	506.5	-	-	-	29.4	59.5	11.1	58.2	72.5	0.2	14.8	42.6	26.3	229.5	194.6	-	34.9	485.6	84.2	34.4	367.0	715.1
4_CxL	47.0	11.1	523.4	34.4	-	21.2	29.2	59.8	11.0	58.4	76.7	0.3	15.2	41.3	26.3	231.8	166.4	36.8	28.6	852.9	247.0	38.0	567.9	1084.7
5_CxL	64.0	12.0	768.3	37.8	67.1	29.4	34.4	53.6	11.8	50.9	71.0	2.5	19.6	32.0	18.4	237.5	170.0	27.4	40.1	1178.6	293.6	80.5	804.5	1416.1
6_CxL	46.0	9.4	432.7	30.1	56.4	20.7	-	-	-	-	-	-	-	-	-	-	-	-	-	-	-	-	-	-
7_CxL	58.0	10.1	586.0	41.4	71.3	32.2	26.6	60.7	12.4	59.2	70.7	1.1	12.5	32.5	29.1	136.6	94.7	41.9	-	730.1	135.8	48.9	545.4	866.7
8_CxL	25.0	6.4	159.7	38.6	69.9	31.2	23.0	64.8	12.2	64.8	76.7	2.0	17.8	34.3	21.7	87.9	87.9	-	-	658.3	66.9	36.2	555.2	746.2
9_CxL	64.0	9.9	636.4	38.4	74.2	25.6	29.3	58.3	12.1	58.3	64.4	1.1	17.3	33.7	21.6	96.6	81.8	14.8	-	644.2	115.1	23.4	505.7	740.8
10_CxL	60.0	9.0	540.5	29.0	60.4	13.5	-	-	-	-	-	-	-	-	-	-	-	-	-	-	-	-	-	-
11_CxL	54.0	7.6	408.7	31.4	68.8	23.3	40.0	46.4	13.0	44.9	61.1	2.1	25.7	29.3	10.3	97.0	97.0	-	-	1091.4	293.3	101.5	696.6	1188.4
12_CxL	43.0	9.9	427.5	27.3	52.6	19.4	28.0	60.8	10.6	60.8	72.2	0.9	16.1	34.7	23.9	236.9	207.9	29.0	-	891.2	200.4	47.5	643.3	1128.1
13_CxL	76.0	6.6	505.0	40.8	74.9	29.0	32.0	57.5	10.1	56.4	63.5	0.5	15.8	42.6	21.4	95.4	95.4	-	-	772.8	185.4	-	587.4	868.2
14_CxL	47.0	8.1	382.3	32.3	65.2	15.9	35.6	52.4	12.1	50.6	64.4	0.7	21.9	39.0	16.0	184.4	107.1	40.0	37.3	696.9	122.5	91.3	483.1	881.3
15_CxL	39.0	7.5	292.6	39.4	71.7	26.0	-	-	-	-	-	-	-	-	-	-	-	-	-	-	-	-	-	-
16_CxL	63.0	7.2	455.1	29.0	47.6	24.7	25.3	63.5	10.8	62.2	64.7	0.6	11.5	34.7	32.0	169.6	94.0	75.6	-	754.8	264.7	38.0	452.1	924.4
17_CxL	-	-	-	-	-	-	39.7	45.5	14.8	44.0	65.5	1.9	24.4	27.8	10.3	154.2	76.2	36.6	41.4	1320.2	306.7	205.3	808.2	1474.4
18_CxL	19.0	7.8	149.0	37.9	71.4	27.0	28.8	59.8	11.4	59.6	71.0	0.4	15.3	41.0	25.7	109.0	44.7	30.9	33.4	493.3	53.1	56.6	383.6	602.3
19_CxL	53.0	11.3	596.8	23.9	61.7	13.6	34.0	52.0	13.6	49.9	68.1	0.4	21.2	37.3	16.0	272.0	181.3	53.8	36.9	1302.4	364.6	145.5	792.3	1574.4
20_CxL	57.0	10.8	617.7	37.9	-	23.1	35.2	52.4	12.4	49.9	64.7	1.1	21.1	39.0	16.9	226.9	141.7	42.9	42.3	1153.0	297.0	69.4	786.6	1379.9
21_CxL	64.0	9.1	582.7	33.0	65.7	23.7	35.7	50.5	13.8	48.1	64.1	-	-	-	-	-	-	-	-	-	-	-	-	-
22_CxL	65.0	9.2	598.9	25.9	70.4	18.2	39.4	44.3	16.1	42.2	69.1	0.6	24.0	32.0	9.9	241.2	146.5	31.5	63.2	1549.9	460.0	192.8	897.1	1791.1
23_CxL	69.0	10.4	716.7	34.8	68.5	17.1	31.0	57.5	11.2	56.4	70.3	1.0	14.3	34.6	30.1	362.9	264.6	98.3	-	835.1	215.3	57.9	561.9	1198.0
24_CxL	50.0	6.2	310.2	32.6	-	21.0	28.1	58.4	13.6	58.1	72.7	-	-	-	-	-	-	-	-	-	-	-	-	-
25_CxL	51.0	9.8	501.9	29.3	68.5	17.4	39.7	45.8	14.4	45.3	70.7	0.8	26.9	33.8	9.7	226.8	146.6	29.8	50.4	1464.0	378.0	171.0	915.0	1690.8
26_CxL	48.0	9.9	476.8	35.6	65.5	23.3	39.7	45.8	14.4	56.4	58.2	0.6	16.1	39.6	24.3	149.0	108.4	40.6	-	931.0	231.0	60.7	639.3	1080.0
27_CxL	44.0	7.8	343.7	37.7	69.6	25.2	39.7	45.8	14.4	60.9	70.3	0.4	12.3	41.6	29.4	160.4	118.2	42.2	-	732.2	143.8	52.3	536.1	892.6
28_CxL	37.0	9.3	344.9	38.3	71.7	26.9	25.0	62.4	12.3	60.9	68.2	0.1	8.9	45.2	31.4	240.1	162.9	38.3	38.9	974.7	234.6	46.4	693.7	1214.8
29_CxL	28.0	12.6	353.2	36.0	68.3	20.6	30.9	55.7	13.2	55.7	70.1	1.2	18.3	33.3	19.3	67.6	51.8	15.8	-	559.7	68.3	39.1	452.3	627.3
30_CxL	45.0	8.0	360.6	44.8	72.3	26.4	40.7	48.6	9.9	47.4	58.2	4.0	26.8	33.1	11.3	234.2	115.9	73.5	44.8	755.4	156.1	68.4	530.9	989.6

31_CxL	80.0	10.7	858.2	29.5	49.0	23.5	32.9	55.5	11.6	53.0	75.7	1.9	18.8	32.4	19.7	59.4	43.8	15.6	-	720.8	200.2	20.8	499.8	780.2
32_CxL	59.0	11.0	647.4	36.5	62.9	18.4	29.7	85.5	11.2	56.7	66.0	0.7	15.6	39.9	18.5	163.3	100.0	30.7	32.6	758.6	170.7	70.7	517.2	921.9
33_CxL	63.0	12.5	784.9	33.7	56.0	20.0	27.6	61.3	10.6	60.0	81.4	0.4	14.0	35.7	27.2	106.3	65.9	40.4	-	1212.6	336.5	35.6	840.5	1318.9
34_CxL	54.0	9.9	536.1	37.2	62.9	29.2	27.9	60.3	11.8	60.1	72.0	0.7	13.6	38.8	25.0	172.0	122.7	49.3	-	1118.7	245.1	59.6	814.0	1290.7
35_CxL	48.0	6.9	331.8	18.5	39.5	12.5	25.0	61.5	13.3	61.5	75.7	1.1	13.3	31.8	28.0	69.9	42.6	27.3	-	385.9	61.8	19.6	304.5	455.8
36_CxL	54.0	8.5	461.2	45.2	77.7	22.8	34.7	52.3	13.0	50.5	66.0	1.2	17.7	35.0	19.7	96.3	57.8	38.5	-	505.4	91.6	35.1	378.7	601.7
37_CxL	66.0	11.2	740.9	37.2	67.9	23.3	20.1	65.2	14.8	65.2	81.4	1.8	16.4	31.8	23.0	18.3	18.3	-	-	679.8	108.4	18.6	552.8	698.1
38_CxL	35.0	9.9	345.2	39.8	70.9	24.8	28.7	59.9	10.8	58.8	69.3	0.8	13.1	33.2	28.0	181.7	108.1	45.2	28.4	915.8	164.0	59.4	692.4	1097.5
39_CxL	51.0	10.4	528.7	31.8	62.2	14.8	32.3	57.0	10.1	55.3	64.3	0.4	18.9	43.2	20.7	115.2	115.2	-	-	726.3	147.0	41.2	538.1	841.5
40_CxL	52.0	9.2	476.3	36.6	-	25.6	42.6	43.3	13.6	41.1	59.1	2.3	27.0	26.9	8.2	344.2	238.9	66.1	39.2	1504.9	403.8	146.3	954.8	1849.1
1_TxA(O)	79.0	13.6	1076.5	20.9	52.1	8.2	37.4	47.4	15.1	45.9	65.8	0.7	24.5	34.3	10.8	308.5	208.4	60.8	39.3	1418.7	380.8	142.2	895.7	1727.2
2_TxA(O)	58.0	10.4	602.5	27.3	54.1	19.5	31.9	55.0	13.2	54.6	69.7	2.7	25.3	27.3	13.5	110.7	84.1	26.6	-	750.5	167.8	59.0	523.7	861.2
3_TxA(O)	71.0	13.5	957.9	26.2	53.7	18.2	36.2	49.3	14.4	48.8	67.0	1.4	20.1	29.9	14.9	141.7	141.7	-	-	1387.1	377.0	183.6	826.5	1528.8
4_TxA(O)	73.0	15.4	1123.9	28.7	52.1	21.7	40.9	43.2	16.0	41.4	63.4	0.7	27.9	30.3	7.0	249.0	185.2	-	63.8	1506.3	526.5	149.8	830.0	1755.3
5_TxA(O)	68.0	12.6	859.0	29.1	53.2	17.4	43.3	41.2	15.3	39.6	60.6	2.1	27.4	25.3	7.3	183.7	149.0	-	34.7	1238.6	438.8	102.4	697.4	1422.3
6_TxA(O)	67.0	13.6	910.4	29.3	52.2	18.8	40.9	43.0	16.1	41.6	63.8	0.7	27.2	28.9	7.2	202.5	123.7	43.8	35.0	880.6	241.9	110.2	528.5	1083.1
7_TxA(O)	62.0	13.9	859.9	22.9	40.9	16.7	40.8	43.9	15.3	43.6	63.5	0.7	27.4	32.4	7.9	215.2	129.9	42.9	42.4	979.6	269.0	98.4	612.2	1194.8
8_TxA(O)	75.0	12.4	933.7	22.6	-	14.1	38.1	46.6	15.2	45.2	65.3	0.5	24.6	33.2	10.0	148.3	65.7	29.5	53.1	1323.3	507.5	145.0	670.8	1471.6
9_TxA(O)	82.0	12.2	999.2	31.3	57.9	20.2	40.0	45.4	14.6	43.6	63.0	0.9	26.9	31.5	7.8	81.8	51.0	-	30.8	705.0	173.9	52.7	478.4	786.8
10_TxA(O)	54.0	15.2	822.4	27.5	51.6	18.2	-	-	-	-	-	-	-	-	-	-	-	-	-	-	-	-	-	-
11_TxA(O)	71.0	11.7	831.1	28.5	48.4	17.5	38.4	46.9	14.4	44.9	63.7	1.7	23.4	28.4	11.9	231.1	143.1	49.6	38.4	973.1	265.0	123.0	585.1	1204.2
12_TxA(O)	59.0	9.7	572.6	25.2	48.3	16.8	36.0	47.5	15.8	45.4	66.6	1.4	20.0	28.1	14.5	379.6	244.1	73.6	61.9	1648.1	477.7	219.0	951.4	2027.7
13_TxA(O)	80.0	13.2	1055.6	29.8	53.1	24.2	38.6	47.2	14.0	45.1	63.4	1.1	29.3	29.9	7.3	176.3	98.9	41.9	35.5	1055.6	302.8	88.7	664.1	1231.9
14_TxA(O)	51.0	11.0	562.8	26.2	-	19.2	39.3	44.4	16.3	42.9	65.2	0.8	26.0	31.1	8.9	125.4	58.6	34.1	32.7	558.3	118.2	74.2	365.9	683.7
15_TxA(O)	49.0	7.2	350.9	28.8	52.4	16.0	38.9	43.7	17.3	42.7	66.9	0.6	25.1	31.1	8.5	191.1	113.0	44.5	33.6	697.4	185.6	74.1	437.7	888.5
16_TxA(O)	40.0	11.3	452.2	29.1	48.8	17.2	40.5	45.5	13.3	43.8	60.8	0.9	29.7	33.7	7.6	241.4	154.9	39.0	47.5	1756.6	509.7	121.7	1125.2	1998.0
17_TxA(O)	17.0	6.3	107.2	15.3	-	5.7	35.7	48.7	15.4	46.8	67.0	0.6	24.6	34.1	10.6	230.1	146.0	37.8	46.3	1235.3	482.1	-	753.2	1465.4
18_TxA(O)	43.0	7.9	341.4	36.0	-	27.7	34.7	51.6	13.4	50.3	66.6	0.6	21.3	38.1	14.3	323.8	226.3	45.3	52.2	1730.1	511.7	211.5	1006.9	2053.9
19_TxA(O)	67.0	11.2	752.1	24.0	-	17.4	40.2	44.7	15.1	42.5	63.2	0.8	27.9	31.5	8.2	125.6	87.0	-	38.6	962.5	312.7	92.2	557.6	1088.1
20_TxA(O)	70.0	13.9	971.5	26.0	50.8	16.1	34.4	48.3	17.3	48.0	71.2	2.1	21.6	25.1	11.4	123.7	82.4	41.3	-	751.7	178.2	91.0	482.5	875.4
21_TxA(O)	50.0	9.5	476.6	24.5	43.5	13.4	38.8	47.8	13.4	47.4	64.0	0.8	27.5	34.0	9.4	224.9	224.9	-	-	1268.2	421.1	94.4	752.7	1493.1
22_TxA(O)	53.0	13.7	724.5	25.6	50.2	17.6	38.0	45.7	15.8	43.2	64.7	0.5	23.7	30.7	10.8	243.0	174.0	-	69.0	1882.0	651.0	262.9	968.1	2125.0
23_TxA(O)	72.0	14.8	1063.2	33.9	58.4	21.2	38.4	47.3	13.5	45.7	62.8	0.9	30.8	25.6	5.5	208.1	106.6	49.3	52.2	1699.1	498.6	261.2	939.3	1907.2

24_TxA(O)	65.0	15.1	983.9	28.5	49.0	19.5	40.2	44.3	15.0	41.8	62.3	1.9	23.9	26.9	10.3	290.4	168.9	62.3	59.2	1609.5	452.8	250.2	906.5	1899.9
25_TxA(O)	51.0	16.4	835.6	30.1	52.1	22.4	38.5	45.8	15.3	44.2	64.6	0.7	25.6	32.4	10.4	139.1	46.3	46.3	46.5	1503.4	370.0	187.6	945.8	1642.5
26_TxA(O)	69.0	14.4	991.0	34.3	54.9	23.7	36.0	49.6	13.5	47.5	64.5	1.3	22.9	30.0	15.0	122.1	71.6	-	50.5	1586.1	440.7	179.5	965.9	1708.2
27_TxA(O)	72.0	13.0	935.1	29.5	54.3	19.8	37.6	48.1	14.2	46.7	64.8	1.6	23.0	30.0	12.4	142.0	142.0	-	-	1859.8	618.7	123.0	1118.1	2001.8
28_TxA(O)	69.0	14.2	981.5	25.2	46.5	17.2	39.7	46.7	13.7	44.8	62.4	0.8	26.0	33.4	10.4	265.1	211.5	-	53.6	2096.4	743.7	153.0	1199.7	2361.5
29_TxA(O)	76.0	14.0	1067.7	25.7	46.4	20.0	40.7	44.1	14.9	42.4	62.4	1.9	23.8	27.2	10.0	166.2	84.5	44.3	37.4	881.7	265.5	106.7	509.5	1047.9
30_TxA(O)	35.0	11.0	384.8	26.9	54.2	14.0	38.0	50.7	11.3	50.0	62.6	2.6	26.7	36.7	11.1	253.2	146.3	51.6	55.3	1718.2	558.7	148.6	1010.9	1971.4
31_TxA(O)	58.0	14.1	815.4	31.6	53.7	20.0	37.1	49.8	12.2	47.5	62.6	0.5	26.0	37.8	11.3	169.9	94.3	38.9	36.7	1278.9	438.7	92.0	748.2	1448.8
32_TxA(O)	56.0	15.4	859.6	36.0	62.3	23.4	35.7	51.7	12.7	51.1	65.8	0.7	23.4	37.2	13.6	206.5	130.1	27.5	48.9	1331.6	358.0	168.7	804.9	1538.1
33_TxA(O)	26.0	8.8	228.5	29.2	49.0	20.2	38.1	46.3	15.6	44.5	65.5	0.7	23.9	32.6	10.0	205.6	115.8	37.4	52.4	1342.5	246.5	252.5	843.5	1548.1
34_TxA(O)	63.0	15.7	991.2	30.0	57.1	22.6	30.0	40.6	11.9	39.4	54.6	0.4	24.3	36.6	13.5	201.7	110.7	51.5	39.5	1350.8	459.8	112.1	778.9	1552.5
35_TxA(O)	37.0	12.4	459.0	32.2	57.7	20.4	38.2	47.3	14.3	45.6	64.4	0.5	25.4	34.6	10.8	176.4	129.2	-	47.2	900.8	-	117.1	783.7	1077.2
36_TxA(O)	68.0	13.9	943.8	27.9	50.4	22.6	37.9	46.8	15.3	45.1	65.4	0.7	25.1	34.6	10.2	166.9	133.2	-	33.7	1174.2	443.2	69.0	662.0	1341.1
37_TxA(O)	56.0	14.6	819.1	28.3	51.3	18.2	41.2	43.6	15.1	41.8	62.3	0.9	29.6	29.7	5.7	175.6	78.4	40.6	56.6	1612.0	359.9	272.2	979.9	1787.6
38_TxA(O)	62.0	14.3	887.5	29.7	53.7	19.4	37.3	47.2	14.7	45.0	64.6	0.6	24.0	34.1	11.1	153.0	119.3	33.7	-	1021.4	330.9	50.9	639.6	1174.4
39_TxA(O)	60.0	12.9	771.8	26.8	48.6	23.1	41.9	41.3	16.8	39.8	63.8	1.0	29.2	28.1	6.1	263.3	168.3	55.4	39.6	1516.4	520.0	93.9	902.5	1779.7
40_TxA(O)	64.0	17.4	1114.1	41.4	65.2	27.7	37.6	48.9	13.5	48.0	63.6	0.5	24.5	34.7	11.4	131.1	99.7	31.4	-	1349.6	421.1	146.9	781.6	1480.7
41_TxA(O)	76.0	16.1	1226.7	31.2	-	22.1	39.5	47.4	13.1	44.9	61.5	1.3	19.8	37.0	16.5	114.2	95.6	18.6	-	817.5	197.3	57.2	563.0	931.7
42_TxA(O)	64.0	12.8	820.4	33.1	60.3	16.0	41.6	43.4	14.7	42.3	62.0	-	-	-	-	-	-	-	-	-	-	-	-	-
43_TxA(O)	65.0	11.3	736.6	26.5	47.6	14.9	37.5	49.6	12.8	48.3	63.8	1.5	23.2	30.9	13.5	244.2	172.8	33.6	37.8	1495.0	423.0	140.9	931.1	1739.2
44_TxA(O)	67.0	14.5	971.4	31.2	57.0	22.9	41.0	44.0	14.4	42.0	61.8	0.5	26.3	34.1	10.0	233.7	160.0	37.0	36.7	1618.9	529.2	124.7	965.0	1852.6
45_TxA(O)	63.0	17.4	1096.5	15.5	45.9	8.9	37.1	49.8	12.2	47.3	62.0	1.4	23.7	30.9	13.0	282.1	186.1	57.7	38.3	1506.5	471.8	108.9	925.8	1788.6
46_TxA(O)	77.0	10.2	788.0	34.3	60.3	24.7	37.9	47.4	14.5	45.2	64.1	0.6	26.2	35.2	9.4	254.3	155.8	43.6	54.9	1738.7	476.5	208.0	1054.2	1993.0
47_TxA(O)	54.0	11.9	644.2	30.2	55.1	18.8	38.8	45.7	14.8	44.3	64.0	2.5	26.6	25.5	10.1	273.7	273.7	-	-	887.1	230.1	102.8	554.2	1160.8
48_TxA(O)	87.0	14.7	1280.9	28.8	50.5	20.8	37.1	49.8	12.2	40.7	62.0	2.3	25.5	25.6	8.1	154.7	98.5	-	56.2	1499.7	369.8	193.0	936.9	1654.4
49_TxA(O)	70.0	13.4	941.3	35.1	60.3	23.6	-	-	-	-	-	-	-	-	-	-	-	-	-	-	-	-	-	-
50_TxA(O)	68.0	14.0	953.1	32.9	-	24.8	38.4	45.7	15.9	44.0	65.6	0.7	25.6	32.1	8.8	229.9	136.6	56.2	37.1	1109.5	265.0	96.4	748.1	1339.4
51_TxA(O)	49.0	18.3	896.9	32.3	57.2	20.2	39.4	47.4	13.2	46.7	61.6	0.9	25.8	33.8	10.0	183.5	95.3	36.4	51.8	1418.5	346.3	226.1	846.1	1602.0
52_TxA(O)	58.0	16.0	925.5	23.8	43.8	15.5	38.7	48.8	12.5	47.0	62.1	0.8	26.7	36.3	10.5	256.2	182.0	27.0	47.2	1387.8	448.2	164.1	775.5	1644.0
53_TxA(O)	81.0	13.1	1058.5	25.4	48.7	17.9	41.4	42.0	15.5	40.5	62.0	0.7	29.4	29.9	6.2	208.3	123.5	45.3	39.5	1293.8	500.1	76.7	717.0	1502.1
54_TxA(O)	63.0	11.8	742.5	33.2	-	20.6	39.1	44.0	16.9	43.7	66.9	0.9	25.8	29.2	8.5	330.0	198.1	-	131.9	1560.0	520.5	210.4	829.1	1890.0
55_TxA(O)	51.0	12.4	633.1	33.7	54.8	24.3	38.2	46.3	14.6	44.1	63.4	0.8	25.5	33.0	9.9	149.5	78.4	31.7	39.4	1221.5	309.4	138.1	774.0	1371.0
56_TxA(O)	64.0	10.6	675.3	25.6	54.1	18.5	39.1	44.7	16.0	42.9	64.7	1.7	22.4	27.2	10.7	139.7	58.6	36.1	45.0	944.8	191.1	160.4	593.3	1084.5

57_TxA(O)	77.0	16.6	1280.4	23.3	48.1	13.0	39.4	46.7	13.9	44.9	62.8	0.5	26.7	33.2	9.6	199.9	121.3	35.1	43.5	1245.2	380.0	138.6	726.6	1445.1
58_TxA(O)	63.0	13.1	825.9	40.5	65.9	30.2	40.3	45.3	14.3	44.0	62.7	0.6	28.1	32.8	8.1	215.9	146.8	38.9	30.2	967.4	263.3	56.8	647.3	1183.3
59_TxA(O)	-	-	-	-	-	-	39.5	48.3	12.1	46.5	61.1	1.0	25.4	31.5	11.9	105.5	68.0	-	37.5	1476.7	497.2	113.5	866.0	1582.2
60_TxA(O)	62.0	12.6	778.4	31.2	57.8	18.8	39.1	49.1	11.7	47.5	61.2	2.0	24.5	31.4	12.7	177.5	131.4	-	46.1	1889.8	633.4	175.8	1080.6	2067.3
61_TxA(O)	63.0	14.2	891.6	28.9	49.1	17.3	39.7	46.5	13.7	44.0	61.8	1.0	27.0	33.8	9.9	217.2	114.4	32.3	70.5	1496.7	456.1	213.8	826.8	1713.9
62_TxA(O)	68.0	15.6	1057.4	35.5	60.2	26.8	40.3	46.8	12.9	44.5	60.8	0.8	29.1	35.3	7.9	248.7	132.5	40.7	75.5	1852.3	560.9	248.0	1043.4	2101.0
63_TxA(O)	57.0	16.5	937.7	29.2	55.4	18.5	41.9	43.8	13.9	41.8	60.2	2.2	26.0	26.0	8.4	133.6	78.5	-	55.1	1779.1	430.9	264.2	1084.0	2349.7
64_TxA(O)	64.0	13.4	859.7	37.6	63.4	25.0	39.0	45.8	14.8	44.1	63.7	1.0	26.2	32.7	9.6	214.7	140.2	29.1	45.4	1594.0	524.5	170.0	899.5	1808.7
65_TxA(O)	76.0	13.6	1031.6	29.9	54.2	21.6	38.0	46.2	15.8	44.8	65.9	-	-	-	-	124.9	124.9	-	-	808.4	48.5	85.5	674.4	933.3
66_TxA(O)	67.0	17.3	1162.4	28.5	52.9	19.7	38.1	48.5	13.1	46.5	62.9	1.6	21.4	27.9	12.2	201.9	115.7	40.5	45.7	1596.8	421.3	233.7	941.8	1798.7
67_TxA(O)	66.0	14.2	938.3	33.7	56.9	20.8	36.8	50.6	12.5	49.2	63.9	1.5	21.6	31.3	14.7	213.7	140.7	36.2	36.8	1406.3	351.5	146.8	908.0	1620.0
68_TxA(O)	82.0	11.6	948.3	27.8	55.2	22.7	41.1	43.8	15.1	42.8	63.2	1.0	27.5	30.7	9.6	231.6	159.3	35.6	36.7	1596.6	572.9	111.7	912.0	1828.2
69_TxA(O)	73.0	13.4	979.9	33.2	55.5	24.7	43.0	41.3	15.6	39.8	61.3	2.3	26.9	25.0	7.6	99.8	57.4	42.4	-	1119.9	98.9	103.8	917.2	1219.7
70_TxA(O)	64.0	14.2	908.7	29.4	59.0	21.0	37.9	50.8	11.1	48.5	60.9	1.7	23.8	31.8	13.4	147.7	147.7	-	-	1550.1	484.3	120.5	945.3	1697.8
71_TxA(O)	76.0	15.6	1187.0	30.8	55.0	22.7	39.9	45.5	14.0	43.1	61.7	1.1	26.2	29.4	9.2	226.4	151.0	29.9	45.5	1569.3	554.5	116.7	898.1	1795.7
72_TxA(O)	77.0	12.6	971.3	43.1	73.5	30.8	-	-	-	-	-	-	-	-	-	-	-	-	-	-	-	-	-	-
73_TxA(O)	70.0	12.5	876.8	20.9	52.1	8.2	-	-	-	-	-	-	-	-	-	-	-	-	-	-	-	-	-	-
74_TxA(O)	54.0	12.2	660.0	32.2	56.8	23.8	-	-	-	-	-	-	-	-	-	-	-	-	-	-	-	-	-	-
75_TxA(O)	48.0	8.7	415.6	28.3	49.6	18.3	-	-	-	-	-	-	-	-	-	-	-	-	-	-	-	-	-	-
1_TxA(RGS)	43.0	15.7	674.0	34.0	57.3	19.1	35.9	50.7	12.9	48.7	64.3	0.5	23.2	39.0	12.8	195.3	155.1	40.2	-	1304.7	431.4	97.8	775.5	1500.0
2_TxA(RGS)	10.0	7.2	71.5	29.2	52.8	11.5	35.7	49.8	14.3	47.8	66.0	1.1	20.0	29.7	14.5	505.9	382.0	78.1	45.8	941.9	264.1	82.2	595.6	1447.8
3_TxA(RGS)	56.0	11.0	617.8	30.6	53.3	23.6	39.6	45.6	14.7	45.2	64.5	0.8	26.3	32.7	10.0	355.8	254.7	43.1	58.0	1258.1	388.8	84.4	784.9	1613.9
4_TxA(RGS)	47.0	8.8	412.1	30.1	51.8	14.3	38.9	48.0	12.7	46.5	62.0	1.4	23.4	29.8	11.7	279.3	197.1	45.9	36.3	1281.5	317.8	84.6	879.1	1560.8
5_TxA(RGS)	26.0	8.9	231.9	25.9	51.0	19.4	-	-	-	-	-	-	-	-	-	-	-	-	-	-	-	-	-	-
6_TxA(RGS)	50.0	14.5	724.4	28.2	53.4	15.2	32.3	55.4	11.5	53.2	65.9	0.9	18.8	34.1	19.9	278.0	177.5	44.1	56.4	1318.1	415.0	98.0	805.1	1596.1
7_TxA(RGS)	1.0	15.0	15.0	-	-	-	-	-	-	-	-	-	-	-	-	-	-	-	-	-	-	-	-	-
8_TxA(RGS)	22.0	5.9	130.6	-	-	-	-	-	-	-	-	-	-	-	-	-	-	-	-	-	-	-	-	-
9_TxA(RGS)	2.0	9.0	18.0	-	-	-	-	-	-	-	-	-	-	-	-	-	-	-	-	-	-	-	-	-
10_TxA(RGS)	66.0	13.4	884.9	28.5	52.3	11.4	41.4	45.9	12.7	45.1	60.9	1.0	29.9	33.5	6.9	262.2	156.9	50.4	54.9	1854.8	507.0	175.7	1172.1	2117.0
11_TxA(RGS)	53.0	12.4	659.3	32.1	53.6	19.2	35.7	52.3	12.0	50.5	64.2	0.2	23.3	38.7	14.4	237.4	145.0	35.2	57.2	1571.8	468.5	160.4	942.9	1809.2
12_TxA(RGS)	44.0	8.4	370.5	27.9	51.0	13.9	39.7	46.8	13.2	45.2	62.0	2.1	23.8	30.3	11.0	186.0	69.8	78.4	37.8	881.5	321.5	88.5	471.5	1067.5
13_TxA(RGS)	69.0	13.1	904.8	29.2	52.3	16.9	35.7	52.3	12.0	50.3	64.2	3.1	26.5	27.9	11.0	121.7	94.5	27.2	-	831.0	209.1	52.5	569.4	952.7
14_TxA(RGS)	2.0	2.7	5.3	31.4	-	20.8	-	-	-	-	-	-	-	-	-	-	-	-	-	-	-	-	-	-

15_TxA(RGS)	13.0	9.0	117.2	-	-	-	40.6	43.9	13.8	40.7	59.7	2.1	25.8	26.7	8.9	435.7	280.5	85.0	70.2	1209.7	352.1	89.1	768.5	1645.4
16_TxA(RGS)	8.0	3.5	28.0	-	-	-	-	-	-	-	-	-	-	-	-	-	-	-	-	-	-	-	-	-
17_TxA(RGS)	47.0	12.3	580.4	27.7	52.8	17.5	37.7	50.5	11.7	48.7	62.2	1.8	23.4	31.9	14.5	257.8	187.7	32.7	37.4	989.1	267.0	92.3	629.8	1246.9
18_TxA(RGS)	1.0	16.0	16.0	24.5	41.9	16.6	-	-	-	-	-	-	-	-	-	-	-	-	-	-	-	-	-	-
19_TxA(RGS)	43.0	7.1	303.8	26.1	46.4	17.2	35.5	48.1	16.1	46.9	68.2	0.5	21.5	35.3	12.9	86.1	51.9	34.2	-	306.8	55.3	39.8	211.7	392.9
20_TxA(RGS)	40.0	10.3	410.9	24.9	53.5	14.9	35.8	53.8	10.4	52.5	63.2	1.2	23.9	40.9	15.7	151.0	75.4	42.2	33.4	566.1	113.7	91.9	360.5	717.1
21_TxA(RGS)	42.0	13.7	576.9	30.9	49.3	17.6	37.0	46.3	16.7	44.1	67.1	0.7	23.1	31.5	10.8	293.7	224.8	32.4	36.5	1011.7	306.5	116.6	588.6	1305.4
22_TxA(RGS)	43.0	10.2	438.3	18.2	36.6	13.4	38.2	47.9	13.8	47.6	63.8	1.0	24.2	35.1	12.6	222.8	148.7	31.4	42.7	1249.5	268.8	150.2	830.5	1472.3
23_TxA(RGS)	34.0	15.0	510.0	34.7	57.5	22.4	39.3	48.2	11.9	45.9	60.4	2.7	24.6	30.2	11.4	250.4	174.7	37.7	38.0	1089.2	400.9	71.1	617.2	1339.6
24_TxA(RGS)	39.0	13.7	534.5	31.2	52.3	22.0	37.8	48.1	12.3	45.9	61.4	1.4	24.8	30.6	12.0	251.4	209.7	-	41.7	1001.8	323.5	96.1	582.2	1253.2
25_TxA(RGS)	53.0	11.0	580.5	30.4	53.4	18.5	36.7	51.5	11.6	50.2	63.2	1.9	22.1	32.2	15.1	164.4	164.4	-	-	915.6	288.7	70.8	556.1	1080.0
26_TxA(RGS)	65.0	12.4	803.6	22.9	44.1	16.1	40.3	45.8	13.7	44.3	61.9	2.0	25.2	29.3	11.5	263.0	167.6	45.2	50.2	1251.4	473.5	100.8	677.1	1514.4
27_TxA(RGS)	36.0	14.4	518.5	33.2	53.0	22.9	36.9	51.0	12.1	50.7	64.6	0.6	25.3	37.1	12.1	195.0	112.7	30.2	52.1	1264.0	347.3	107.0	809.7	1459.0
28_TxA(RGS)	60.0	13.6	817.0	27.1	49.2	15.8	37.1	51.5	11.3	49.9	62.5	1.5	22.7	32.7	14.5	205.0	205.0	-	-	862.9	250.4	63.2	549.3	1067.9
29_TxA(RGS)	47.0	6.4	301.1	26.2	44.9	12.1	-	-	-	-	-	-	-	-	-	-	-	-	-	-	-	-	-	-
30_TxA(RGS)	70.0	11.4	796.6	28.4	50.7	17.2	36.0	49.5	14.4	47.7	66.0	2.6	27.1	26.3	8.2	272.6	178.1	49.7	44.8	981.1	274.1	53.1	653.9	1253.7
31_TxA(RGS)	65.0	15.8	1026.8	30.2	57.0	23.3	41.6	45.0	13.2	42.7	59.7	2.3	25.9	27.1	10.1	118.7	118.7	-	-	1237.1	426.6	80.6	729.9	1355.8
32_TxA(RGS)	17.0	9.0	153.0	-	-	-	-	-	-	-	-	-	-	-	-	-	-	-	-	-	-	-	-	-
33_TxA(RGS)	66.0	13.6	895.7	29.2	56.2	20.5	39.6	47.0	13.1	46.1	62.5	1.9	25.3	29.5	10.7	142.6	142.6	-	-	1263.9	424.8	91.2	747.9	1406.5
34_TxA(RGS)	49.0	12.5	611.3	30.8	51.2	13.6	37.9	49.2	12.5	47.5	62.7	1.7	23.3	30.8	13.4	266.4	200.0	35.3	31.1	859.4	178.9	67.5	613.0	1125.8
35_TxA(RGS)	62.0	13.3	825.3	31.0	53.5	17.7	39.8	47.4	12.5	45.3	60.7	-	-	-	-	475.3	387.7	42.6	45.0	1115.6	382.2	171.4	562.0	1590.9
36_TxA(RGS)	49.0	11.7	575.6	30.7	52.0	13.8	38.7	48.4	12.5	47.0	62.1	1.7	24.8	29.7	11.8	279.7	235.1	44.6	-	1001.9	191.0	108.4	702.5	1281.6
37_TxA(RGS)	33.0	6.8	223.8	33.4	51.8	20.6	39.4	47.7	12.9	47.3	63.1	0.9	29.5	34.0	8.0	351.9	228.7	59.9	63.3	1345.3	259.4	224.8	861.1	1697.2
1_TxY	53.0	5.9	311.7	25.8	47.0	17.1	38.8	45.8	15.1	45.8	65.4	1.8	25.2	27.9	9.9	62.3	48.0	14.3	-	779.1	189.4	48.6	541.1	841.4
2_TxY	25.0	9.5	236.3	23.1	46.4	9.0	38.5	49.7	11.2	47.8	60.8	1.9	24.7	31.4	12.4	240.2	151.5	50.9	37.8	1167.8	369.5	66.1	732.2	1408.0
3_TxY	60.0	12.7	762.5	25.8	49.3	15.2	39.1	48.8	11.5	46.3	59.9	0.5	26.0	36.7	10.6	249.7	169.2	44.4	36.1	1091.6	340.4	99.0	652.2	1341.3
4_TxY	-	-	-	-	-	-	38.2	49.1	12.1	48.1	60.9	1.7	25.0	31.0	11.4	383.4	332.7	-	50.7	1547.1	535.5	112.3	899.3	1930.5
5_TxY	38.0	14.8	561.2	34.4	57.9	14.0	34.6	52.5	12.9	52.0	67.0	2.5	25.5	27.7	13.2	167.7	146.9	20.8	-	566.9	111.5	66.3	389.1	734.6
6_TxY	7.0	3.4	24.0	-	-	-	-	-	-	-	-	-	-	-	-	-	-	-	-	-	-	-	-	-
7_TxY	27.0	9.4	253.6	41.1	60.5	21.6	41.5	46.4	12.1	44.0	59.1	1.3	25.5	30.5	9.7	95.2	84.7	10.5	-	837.6	177.1	84.3	576.2	932.8
8_TxY	17.0	6.7	114.2	28.4	50.4	11.2	41.0	44.8	14.0	42.6	61.1	0.7	29.2	32.1	7.4	450.4	366.2	84.2	-	1585.8	344.7	96.4	1144.7	2036.2
9_TxY	71.0	12.6	896.8	24.7	48.0	13.6	40.2	44.5	15.1	42.2	62.6	2.0	23.5	25.8	10.1	287.0	211.0	37.9	38.1	1198.7	367.9	103.3	727.5	1485.7
10_TxY	43.0	9.0	386.6	30.1	53.6	20.7	45.1	37.5	17.3	35.0	60.5	1.1	28.2	21.4	4.6	96.8	96.8	-	-	922.0	274.4	52.5	595.1	1018.8

11_TxY	41.0	9.1	373.7	29.3	57.8	19.5	37.9	48.1	13.8	46.3	63.7	1.8	22.3	29.5	13.1	302.4	266.5	35.9	-	1152.0	323.4	84.2	744.4	1454.4
12_TxY	9.0	3.1	27.8	22.8	45.4	12.4	-	-	-	-	-	-	-	-	-	-	-	-	-	-	-	-	-	-
13_TxY	25.0	8.5	211.7	25.5	45.5	16.4	41.0	46.8	12.2	46.5	60.1	1.0	29.3	35.2	8.5	361.6	254.9	54.3	52.4	1063.3	304.4	100.8	658.1	1424.9
14_TxY	63.0	13.6	854.8	24.0	45.4	14.2	40.8	44.4	14.4	42.3	61.6	2.0	24.0	31.2	13.3	303.0	264.8	38.2	-	1010.9	340.3	84.9	585.7	1313.9
15_TxY	23.0	5.1	117.8	31.1	54.2	23.1	39.8	45.2	14.6	43.5	62.8	2.1	23.8	27.0	10.9	254.0	208.9	45.1	-	1048.8	350.4	50.1	648.3	1302.8
16_TxY	73.0	11.4	834.8	33.8	55.6	17.4	41.0	47.3	11.8	44.5	59.2	2.3	26.0	29.3	10.0	179.3	126.9	52.4	-	1241.1	339.1	68.2	833.8	1420.4
17_TxY	40.0	9.1	365.2	30.4	53.2	21.7	45.3	38.5	16.0	36.2	59.0	1.2	33.0	25.3	3.3	190.4	153.6	36.8	-	1205.2	364.3	94.2	746.7	1395.6
18_TxY	58.0	13.1	759.0	30.7	52.7	22.3	40.3	45.9	12.8	42.9	59.5	-	-	-	-	320.5	243.5	77.0	-	1103.1	382.7	116.6	603.8	1423.6
19_TxY	56.0	14.1	791.4	20.8	42.6	15.1	36.1	49.0	15.0	48.7	67.7	3.5	25.1	26.2	10.7	173.9	152.2	21.7	-	611.4	164.0	35.1	412.3	785.3
20_TxY	22.0	12.7	278.7	30.9	51.6	17.7	39.3	45.8	14.4	43.4	62.6	0.6	26.3	33.0	8.7	307.4	228.5	41.1	37.8	1006.3	374.5	84.9	546.9	1313.7
21_TxY	51.0	14.4	733.3	33.0	54.6	21.7	40.7	44.8	14.1	42.5	61.0	1.8	24.2	27.2	10.5	239.8	201.4	38.4	-	823.0	192.3	55.5	575.2	1062.8
1_TxE	-	-	-	-	-	-	-	-	-	-	-	-	-	-	-	-	-	-	-	-	-	-	-	-
2_TxE	1.0	15.0	15.0	-	-	-	-	-	-	-	-	-	-	-	-	-	-	-	-	-	-	-	-	-
3_TxE	1.0	10.5	10.5	-	-	-	44.7	40.0	15.3	38.1	57.4	1.7	33.2	26.3	4.0	142.0	76.1	28.3	37.6	820.2	210.7	70.7	538.8	962.2
4_TxE	58.0	10.8	624.2	19.3	38.7	11.9	35.0	51.9	13.1	51.6	67.1	2.6	24.9	28.6	12.3	172.9	172.9	-	-	1138.5	255.9	61.8	820.8	1311.4
5_TxE	66.0	9.4	617.4	18.5	37.2	13.1	39.1	47.2	13.8	45.2	63.5	1.3	23.7	28.7	11.8	-	-	-	-	-	-	-	-	-
6_TxE	12.0	5.2	62.3	-	-	-	-	-	-	-	-	-	-	-	-	-	-	-	-	-	-	-	-	-
7_TxE	52.0	10.2	532.9	25.3	50.9	16.8	42.8	43.6	13.4	41.1	58.8	1.8	26.9	26.9	8.0	310.2	197.3	66.5	46.4	1464.9	409.2	106.2	949.5	1775.1
8_TxE	2.0	7.0	14.0	-	-	-	-	-	-	-	-	-	-	-	-	-	-	-	-	-	-	-	-	-
9_TxE	56.0	10.0	557.6	22.9	48.5	11.9	37.7	48.9	13.0	47.1	63.5	0.6	25.8	36.6	11.4	203.7	169.8	33.9	-	844.7	243.3	54.1	547.3	1048.4
10_TxE	12.0	4.0	48.0	21.4	44.4	14.0	40.4	44.7	14.8	42.9	62.8	0.8	27.8	32.4	7.5	154.2	123.6	30.6	-	738.4	231.0	53.1	454.3	892.6
11_TxE	1.0	16.0	16.0	34.2	57.5	15.3	-	-	-	-	-	-	-	-	-	-	-	-	-	-	-	-	-	-
12_TxE	34.0	9.6	326.8	27.3	47.8	15.2	37.9	49.6	12.1	43.0	62.0	-	-	-	-	-	-	-	-	-	-	-	-	-
13_TxE	8.0	3.6	28.5	22.1	41.0	11.4	42.1	43.8	14.1	41.4	60.3	1.4	23.2	31.0	13.6	468.1	335.8	89.2	43.1	1556.1	350.3	152.3	1053.5	2024.2
14_TxE	47.0	13.6	640.1	20.9	35.7	10.0	-	-	-	-	-	-	-	-	-	-	-	-	-	-	-	-	-	-
15_TxE	73.0	9.5	693.5	26.4	49.2	14.0	39.3	45.2	15.6	43.0	64.2	0.8	26.5	31.8	8.2	318.4	229.8	50.5	38.1	1099.4	302.7	106.3	690.4	1417.8
16_TxE	50.0	12.4	617.6	28.0	47.5	14.4	42.1	43.8	14.1	48.5	60.3	1.8	21.2	30.5	14.9	624.2	467.4	77.6	79.2	1643.4	545.4	122.9	975.1	2267.6
17_TxE	43.0	8.5	366.6	-	-	-	38.8	45.8	13.8	44.4	62.4	0.7	26.5	34.2	9.9	376.9	278.4	63.7	34.8	1478.2	358.7	150.7	968.8	1855.1
18_TxE	56.0	11.1	619.1	29.4	47.7	16.0	39.7	47.4	12.9	45.9	60.5	0.7	27.8	35.0	8.9	125.6	84.9	40.7	-	684.8	139.4	63.1	482.3	810.4
19_TxE	52.0	11.7	610.7	27.8	50.1	19.8	37.2	49.6	13.2	48.3	61.9	0.6	24.0	36.2	12.2	101.0	63.6	37.4	-	589.6	119.0	77.3	393.3	690.6
20_TxE	2.0	11.2	22.4	26.2	-	18.9	38.4	48.2	13.3	47.5	61.2	0.8	26.4	35.0	10.7	186.5	100.8	47.6	38.1	1288.8	356.4	140.7	791.7	1475.3
21_TxE	48.0	9.0	431.3	20.8	45.7	10.6	36.6	47.4	15.2	45.6	65.8	0.4	23.1	34.1	11.5	432.0	329.3	55.7	47.0	1035.4	349.9	54.9	630.6	1467.4
22_TxE	4.0	9.5	38.0	-	-	-	-	-	-	-	-	-	-	-	-	-	-	-	-	-	-	-	-	-

23_TxE	56.0	9.5	532.4	24.2	44.2	16.0	38.7	49.2	11.6	46.7	60.5	2.0	24.0	26.7	10.4	305.3	237.4	67.9	-	1375.5	457.1	94.3	824.1	1680.8
24_TxE	50.0	11.7	585.2	26.0	44.9	10.3	39.4	47.0	13.3	44.4	61.4	0.8	28.0	34.5	8.6	436.1	303.4	70.8	61.9	1639.1	475.2	126.8	1037.1	2075.2
25_TxE	2.0	6.6	13.2	-	-	-	-	-	-	-	-	-	-	-	-	-	-	-	-	-	-	-	-	-

CxL; Coari x La Mé; TxA(O): Taisha x Avros (Oleoflores); TxA(RGS) Taisha x Avros (RGS); TxY: Taisha x Yangambi; TxE: Taisha x Ekona.

Table S5: Universal adapters and MID sequences used for generating barcoded amplicons of the different Candidate Genes (CG). The CG specific parts of the fusion primers are specified in Table A and replace "**X**" in 1a and 1b primers below. Universal UniA and UniB parts are in *italics*.

No	Name	Primer Sequence
**1a**	**UniA_X(CG)**	Fw: *GCAAGACTCGAGCATCTCCA***X**
**1b**	**UniB_X(CG)**	Rv: *GCGATCGTCACTGTTCTCCA***X**
**2a**	**Barcode_UniA**	Fw: CATCTCATCCCTGCGTGTCTCCGA*CTCAG***[MID1]***GCAAGACTCGAGCATCTCCA*
**2b**	**Barcode_UniB**	Rv: CCTCTCTATGGGCAGTCGGTGAT**[MID2]***GCGATCGTCACTGTTCTCCA*

3 MID Primers and their reverse complements (rev com)

	MID (5′->3′)	rev com		MID (5′->3′)	rev com		MID (5′->3′)	rev com
**A**	ACGCTCAG	CTGAGCGT	**G**	TATGCTAGA	TCTAGCATA	**L**	GCTATGACAG	CTGTCATAGC
**B**	TACATCAT	ATGATGTA	**H**	CGCACTGAG	CTCAGTGCG	**M**	ATACATAGCT	AGCTATGTAT
**C**	CGCGACTA	CTGAGCGT	**I**	CAGACTCTA	TAGAGTCTG	**N**	GACAGCGCGT	ACGCGCTGTC
**D**	AGCTAGTC	GACTAGCT	**J**	TGTGAGCAC	GTGCTCACA	**O**	CATGTCAGTA	TACTGACATG
**E**	CGAGATCA	TGATCTCG	**K**	GCGATAGTAC	GTACTATCGC	**P**	ACGCACTCGC	GCGAGTGCGT
**F**	TCAGTGCTG	CAGCACTGA						
